# The Geography of Mediterranean Benthic Communities Under Climate Change

**DOI:** 10.1111/gcb.70725

**Published:** 2026-01-29

**Authors:** Damiano Baldan, Yohann Chauvier‐Mendes, Diego Panzeri, Gianpiero Cossarini, Cosimo Solidoro, Vinko Bandelj

**Affiliations:** ^1^ National Institute of Oceanography and Applied Geophysics – OGS Trieste Italy; ^2^ National Biodiversity Future Center – NBFC Palermo Italy; ^3^ Department of Aquatic Ecology Eawag, Swiss Federal Institute of Aquatic Science and Technology Dübendorf Switzerland; ^4^ Department of Evolutionary Genetics WSL, Swiss Federal Institute for Forest, Snow and Landscape Birmensdorf Switzerland

**Keywords:** alpha diversity, beta diversity, depth migration, global change, habitat fragmentation, marine fauna, seafloor organisms, species distribution models

## Abstract

Seafloors are crucial to marine ecosystems for the functions and services they provide. Benthic organisms, vital to these ecosystems, are particularly vulnerable to climate change. Rising temperatures, ocean acidification, and shifting currents disrupt benthic species and communities, yet future related impact assessments remain limited. Here, we trained species distribution models with predictors from state of the art physical and biogeochemical marine models and a large database of species records (> 100,000 occurrences) to project the current and future distributions of ~350 benthic species (excluding cephalopods, invasive species, and commercially exploited species) and their related changes per site in diversity (α‐) and community composition (β‐diversity) over the Mediterranean Sea. We predicted most species to shift their distribution northwards for all future scenarios due to changes in water temperature and dissolved oxygen close to the seafloor, with up to 60% of species experiencing range contraction, 77% moving northwards, 20% experiencing range fragmentation (measured as range disjunctions in models' output), and 30% moving toward deeper waters over time. Cold‐adapted species were more likely to face range contraction and shifts towards deeper waters, while warm‐adapted species were more likely to face range expansions and shifts towards shallower waters. α‐diversity increased in the Northern and decreased in the Southern Mediterranean, respectively. Changes in β‐diversity within sites highlighted compositional changes (species turnover) in communities located in the Aegean and Tyrrhenian Seas, in deep parts of the Ionian Sea, and in coastal regions of the Adriatic Sea. Climate‐smart, ecosystem‐based Marine Spatial Planning can capitalize on the identified hotspots of species losses, gains, stability, and turnover. Prioritizing connectivity in regions of strong turnover and extending protected areas in regions with stable α‐diversity and limited turnover is recommended for improved conservation actions.

## Graphical abstract

We employed Species Distribution Models with environmental variables from state of the art physical and biogeochemical marine models and a large database of species records to project the current and future distributions of ~350 benthic species in the Mediterranean Sea. Our results show that, due to changes in water temperature and dissolved oxygen close to the seafloor, most species are projected to shift their distribution northwards, with several species experiencing range contraction, range fragmentation, and moving toward deeper waters over time. Climate‐smart, ecosystem‐based Marine Spatial Planning can capitalize on the identified hotspots of species losses, gains, stability, and turnover.

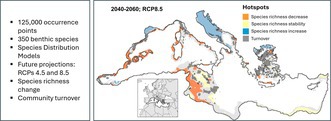

## 1 Introduction

Climate change is strongly impacting marine systems, where profound modifications of physical and biogeochemical properties of sea water are already observed and projected to increase in the future (Burrows et al. 2011; Masson‐Delmotte et al. 2021). Benthic organisms are a key component of marine ecosystems, providing essential ecosystem functions such as bioturbation (Kristensen et al. 2012), linking benthic‐pelagic compartments (Griffiths et al. 2017), increasing substrate heterogeneity (Meadows et al. 2012), and providing a food source for higher trophic levels (Kopp et al. 2015). Benthic communities are impacted by climate change due to their high sensitivity to warming (Birchenough et al. 2015; Hoppit and Schmidt 2022); additionally, their limited dispersal capacity makes them a vulnerable component of marine ecosystems (Hiddink et al. 2015).

Species responses to novel environmental conditions include spatial displacements to track ecological niches, resulting in geographic shifts of the occupied area (Poloczanska et al. 2016). Such redistribution is particularly striking in marine ecosystems, where species generally occupy their full latitudinal ranges (Sunday et al. 2012) and can effectively track climate change (Fredston et al. 2021), supposedly due to the lack of major dispersal barriers (Heino et al. 2015). Climate change is reshaping marine species distributions not only across geographic space, but also through shifts in depth. To keep pace with rising ocean temperatures, many species adjust their position in the water column, moving to cooler, deeper habitat (Chaikin et al. 2022; Hiddink et al. 2015). These shifts, both horizontal and vertical, often lead to changes in range fragmentation, altering how continuous or connected populations are, further influencing the trajectory and extent of future distributional shifts (Curd et al. 2023). Species frequently inhabit fragmented landscapes, where networks of interconnected populations rely on the spatial configuration of habitats and species' dispersal capacity for local coexistence, survival and colonization (Årevall et al. 2018; Crooks et al. 2017; Mestre et al. 2017; Opdam and Wascher 2004; Padmanabha et al. 2024). Changes in species fragmentation can alter this connectivity between inhabited fragments and lead to increased local extinction risks, especially for those species with limited dispersal capacity (Thompson et al. 2020).

In the Mediterranean Sea, the effects of climate change are expected to be stronger than in open oceans both in terms of warming (Cramer et al. 2020) and in terms of biotic responses (Chust et al. 2024; Lejeusne et al. 2010). The colder northern side of the Mediterranean Sea could act as refugia for cold‐adapted species; however, those areas could also become a dead end in case of intensified warming, as the presence of a solid northern boundary prevents species from further moving northward (Ben Rais Lasram et al. 2010). Furthermore, the northern side of the Mediterranean being characterized by semi‐enclosed basins (Northwestern Mediterranean, Adriatic Sea, and Aegean Sea), species migrating northwards might be more prone to range fragmentation. Climate change also impacts the future occurrence of habitat‐forming species such as seagrass, canopy‐forming macroalgae, coralligenous substrates, and deep‐water corals (Descourvières et al. 2024; Georges et al. 2024; Gómez‐Gras et al. 2021; Manca et al. 2024), with cascading effects on habitat connectivity and fragmentation (Baldan et al. 2025). Therefore, a comprehensive assessment of benthic species vulnerability to climate change is urgently needed in the Mediterranean Sea and should account for the full multidimensionality of distributional shifts, including horizontal (geographic), vertical (depth‐related), and fragmentation (connectivity and continuity) changes. These spatial changes at the species level are also mirrored at the community level, where climate‐induced biodiversity redistribution leads to altered species assemblages. Notably, increases in warm‐adapted species (tropicalization) and losses of cold‐adapted species (deborealization) are already well‐documented patterns (Chust et al. 2024). Such compositional shifts can arise through species loss or gain (nestedness), species replacement (turnover), or both (Albouy et al. 2012). Importantly, communities undergoing turnover may retain similar levels of species richness while undergoing major changes in species identity and function (Blowes et al. 2019; García Molinos et al. 2016). Therefore, incorporating turnover‐ and nestedness‐related dynamics into vulnerability assessments is crucial to fully capture how climate change reshapes ecosystems, not just in terms of species counts, but in terms of structure, function, and resilience.

Previous work on species distributional shifts in Mediterranean biodiversity under climate change focused either on fish (Albouy et al. 2013; Ben Rais Lasram et al. 2010) or on commercially exploited species (Moullec et al. 2022), leaving significant knowledge gaps in the future distribution of many benthic organisms, even for species protected by the Barcellona convention (Stranga et al. 2024). Further, the effects on depth and fragmentation are generally overlooked, with significant effort in predicting future range shifts only targeting specific groups such as mollusks (Schultz et al. 2024) or cephalopods (Chaikin et al. 2022).

Here, we investigated how species and biodiversity distribution in the Mediterranean Sea is projected to change under present and future climate change scenarios. For this, we employed species distribution models (SDMs) along physical and biogeochemical projections and a large database of species records (> 100,000 occurrences) to project the current and future habitat suitability of ~350 benthic species and their related changes per site in α‐ and β‐diversity. First, we assessed species‐specific responses to climate change in terms of latitudinal, range, depth, and fragmentation changes and used an ecological indicator value (EIV) approach to relate them to current environmental and distribution metrics. Then, we assessed the response at the community level in terms of species richness, compositional turnover, and nestedness and mapped hotspots of community stability and change. We expect: (i) all species to experience northwards migration, with cold‐adapted species undergoing range contractions and migrating to deeper waters; (ii) species that undergo northwards migration to also experience increases in fragmentation; (iii) areas in the northern Mediterranean to experience increases in species richness, while areas in the southern Mediterranean to experience decreases in species richness; (iv) areas with stable species richness to experience compositional changes (turnover).

## 2 Methods

### 2.1 Species Occurrences

We retrieved occurrence data from three distinct data sources: the Ocean Biodiversity Information System (OBIS), the Global Biodiversity Information Facility (GBIF), and the Bottom Trawl Survey for the Mediterranean (MEDITS, a trans‐national scientific survey using standardized gear with a small mesh size; see Spedicato et al. 2019). We downloaded the OBIS and GBIF data for the Mediterranean region using the R packages “robis” and “rgbif,” respectively. MEDITS data were acquired from the Joint Research Center database (JRC 2025). Data covering the period 2000–2020 were retained. Preprocessing of data consisted in flagging and removing data with incorrect or imprecise coordinates, data falling on the land, and data classified as museum specimens. Next, we performed a taxonomic check by comparing the list of species available in the retrieved data with the taxonomy accepted by the World Register of Marine Species (WORMS) using a fuzzy match algorithm (“worrms” package) to aggregate occurrences according to the WORMS backbone. Finally, to match the occurrence data with the spatial resolution of the physical and biogeochemical predictors used as predictors in the SDMs (see Section 2.4), one occurrence record was retained per each ~4.5 × 4.5‐km grid cell (Figure S1).

**FIGURE 1:**
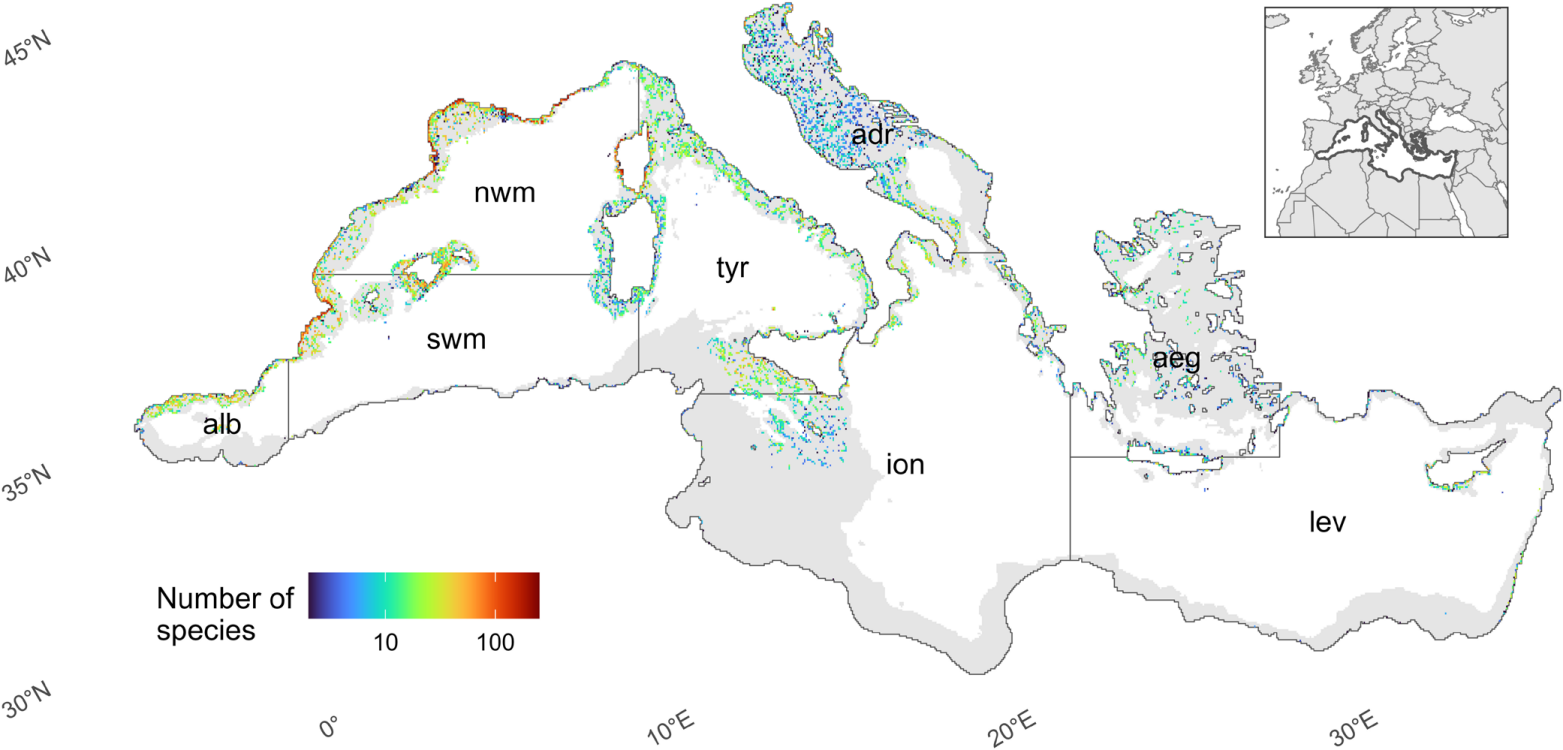
Spatial distribution of points used to fit the models. The gray‐shaded area represents the fraction of the Mediterranean Sea with depth shallower than 800 m, where species distribution models were trained and projected. The insert on the bottom‐left corner shows the geographic position of the studied area (borders are highlighted with a thick line). The labels represent the Mediterranean basins used in the paper; adr, Adriatic sea; aeg, Aegean sea; alb, Alborean Sea; ion, Ionian sea; lev, Levantine sea; nwm, North‐west Mediterranean; swm, South‐west Mediterranean; tyr, Tyrrhenian sea. Map lines delineate study areas and do not necessarily depict accepted national boundaries.

We retained species present in at least *n* = 120 grid cells (i.e., 0.2% of the studied area): this sample size guarantees SDMs to be robust enough to be used for projections (see Section 2.4; Wisz et al. 2008). We removed Cephalopoda as they have a sensibly higher mobility than other classes. We also removed invasive species (*Callinectus sapidus*, *Percnon gibbesi* ) and commercially exploited species ( *Parapenaeus longirostris* , *Aristaeomorpha foliacea* , *Aristeus antennatus* , *Nephrops norvegicus* ), as they might not be in equilibrium with the environmental conditions. The resulting dataset comprising ~125,000 occurrence records for 353 species and 20 taxonomic classes was used to fit the models (Figure 1; see Table S1 in the Supporting Information for the full species list).

### 2.2 Environmental Data

Environmental predictors used to fit SDMs were obtained from the Copernicus Marine Service (CMS) high resolution reanalysis of the Mediterranean Sea physics (Escudier et al. 2021) and biogeochemistry (Cossarini et al. 2021). The reanalysis products have a resolution of ~4.5 km and 125 uneven vertical levels (thickness ranging between 2 and 100 m, increasing with depth). We downloaded the 3D monthly fields of currents speed (Vm), temperature (T), salinity (SAL), ammonium concentration (NH_4_), nitrate concentration (NO_3_), phosphate concentration (PO_4_), oxygen concentration (O_2_), and chlorophyll concentration (CHL) for the period 2000–2020. For all the variables, we selected the vertical level just above the sea bottom. We considered the depth range 0–800 m, as few occurrences were available for deeper regions. Next, we calculated the median, maximum, minimum, and range (maximum–minimum) for the period 2000–2020. Then, all variables were interpolated to fit the resolution of climate projections data (approximately 6.5 × 6.5‐km grid; see Section 2.3). Finally, we included the high‐resolution sediment data based on the seafloor sediment map produced by the European Marine Observation and Data Network (EMODnet; Kaskela et al. 2019). These additional predictors included the fraction of grid cell covered by sand (SAND), mud (MUD), and mixed sediment (MIX).

To retain a parsimonious subset of predictors for fitting the model and avoid overfitting, we performed a hierarchical cluster analysis based on a distance matrix, where we used 1 minus the absolute value of Spearman's correlation coefficient (1 − |*r*|) as a measure of predictors' dissimilarity. We used the threshold of *r* = 0.7 to identify 11 clusters of predictors and retained one predictor for each cluster based on its ecological meaning and interpretability (Figure S1). Finally, we used the Variance Inflation Factor (VIF, function “vifstep” in the package “usdm”) to remove multicollinear predictors, resulting in a list of seven predictors, namely: O2_median_, CHL_median_, T_median_, T_min_, SAL_median_, Vm_median_, and SAND used to fit the models. The selected predictors have a direct effect on species physiology. Temperature and oxygen directly regulate species metabolism and therefore their growth, reproduction, and survival (Pinsky et al. 2020). Chlorophyll (representing primary production) and salinity (representing fluvial influences) are proxies for particulate organic matter, relevant for filter‐feeders (Schultz et al. 2024). Current velocities and sediment texture inform on the hydrodynamics of the seafloor as well as the species' preference for different substrate composition (Beauchard et al. 2022). We did not include depth because it represents an indirect gradient (Guisan and Zimmermann 2000). In fact, depth has no direct physiological relevance for species performances and its scaling with the selected predictors is basin‐dependent. For instance, the Eastern Mediterranean is warmer and more oligotrophic than the Western Mediterranean, while the Adriatic Sea has higher nutrient content due to fluvial influences (Cossarini et al. 2021; Escudier et al. 2021).

### 2.3 Climate Projections Data

Future projections of physical and biogeochemical predictors were obtained from climate simulations (Lovato et al. 2013; Reale et al. 2022; Solidoro et al. 2022) covering the period 2005–2100 under Representative Concentration Pathways (RCP) 4.5 and 8.5 emission scenarios (Harrison et al. 2019; Taylor et al. 2012). The 3D monthly fields of the two simulations have a resolution of ~6.5 km (eddy resolving) and 70 unevenly vertical levels. To separate long‐term potential model drifts from climate signals, the linear trend calculated with a control simulation built by repeating the 2005–2014 time window over the remaining 2015–2100 period was subtracted from each 3D physical and biogeochemical field (see Solidoro et al. 2022 for more details). We considered three time windows: 2005–2025 (present), 2040–2060 (near future), and 2080–2100 (far future). We initially processed the data following the same workflow used for the reanalysis data (see previous section). Then, in each future time window and under a specific emission scenario, and for each predictor, we computed the climatic anomaly as the relative change of the climate signal with respect to the present of the climate simulation (2005–2024). Finally, we calculated the future conditions for each scenario by multiplying the reanalysis value (period 2005–2020) for the future climatic anomaly.

### 2.4 Species Distribution Models

We used the “biomod2” package (Thuiller et al. 2009) to fit, evaluate, and project the species distribution models in the R v4.4.1 computing environment (R Core Team 2022).

For each species, we used the retrieved occurrences and 10,000 pseudo‐absence (P‐Abs) data points to fit the models (Barbet‐Massin et al. 2012; Chauvier et al. 2022). We combined two different sampling strategies to generate our P‐Abs: “target‐group density dependent” and “environmentally stratified.” The first half of the P‐Abs (*n* = 5000) was generated according to the density‐dependent strategy because the clustered distribution of occurrences suggests a strong sampling bias towards coastal regions of the northern side of the Mediterranean. To correct this bias, we sampled the P‐Abs based on the kernel smoothing intensity of all occurrences pooled by class (Figure S2). This method generated a higher density of P‐Abs closer to species observations, mimicking the same sampling effort distribution (Chauvier et al. 2021; Descombes et al. 2022; Righetti et al. 2019). The other half of P‐Abs (*n* = 5000) was sampled based on a stratified subdivision of the environmental range to guarantee that all environmental strata were represented in the training dataset (Da Re et al. 2023; Descombes et al. 2022; Righetti et al. 2019). For this, we classified the median temperature and the median salinity into three bins (splitting data at the 0.3 and 0.6 quantiles) of continuous values, re‐grouped them into unique categories of temperature and salinity (*n* = 9), and sampled an equal number of P‐Abs for each combination. We repeated the P‐Abs generation three times.

We fitted four types of models: a generalized linear model (GLM), a generalized additive model (GAM), a random forest (RF), and an artificial neural network (ANN). We fitted intermediate complexity models to balance the fitting capacity with the ability to extrapolate to unsampled environmental conditions, that is, to prevent overfitting following the guidelines in Brun et al. (2020). We fitted GLMs with linear and quadratic terms; we set the regularization in the GAM smooth term to 3; we set a minimum size of 40 for terminal nodes, a number of 1000 trees, and a number of two covariates used for each tree for RF. We trained ANN with three hidden layers and a decay of 0.1, which we found appropriate to avoid overfitting for the specific datasets. We used the biomod2 default setting equal prevalence of occurrences and P‐Abs (i.e., the sum of the occurrences weights equals the sum of P‐abs weights). We used a spatial block approach to split the data (occurrences and P‐abs) in training (80%) and evaluation (20%) datasets. Spatial clusters were defined using a k‐medoid approach (“pam” function in the “cluster” package, see Brun et al. (2020) for details on the method). We employed a five‐fold cross validation (of the training data, 80% were used for calibration, 20% for validation), that was repeated three times, yielding a total amount of 180 models per species (5 cross validation folds × 3 repetitions × 3 P‐Abs datasets × 4 models). We used the Area under the Receiving Operating Curve (AUC) and the True Skill Statistics (TSS; Allouche et al. 2006) to evaluate the performance metrics of each model for calibration, testing, and evaluation data sets. Additionally, we assessed the model's performance using Boyce's index, a presence‐only metric (Hirzel et al. 2006).

We generated a model ensemble for each species by considering only models with evaluation AUC > 0.7. We assessed the importance of predictors in the final ensemble using a permutation approach. Following this method, each predictor's values were randomly permuted, and model predictions were made accordingly. Each prediction was then compared to the same prediction with and without permutation using Pearson's correlation coefficient (*ρ*). Predictor importance was determined as 1 − |*ρ*|. This process was repeated three times for each predictor, and the average variable importance was considered. The ensemble performance was assessed for both training and evaluation datasets. We considered only ensemble models with evaluation AUC > 0.75, resulting in 339 species modeled out of 353.

### 2.5 Species Habitat Projections

For each species, we projected the models over the two RCP scenarios and the two time windows. Models' projections were cropped by assuming that the future species‐specific upper depth ranges could be 30% shallower than the current 5th depth percentile, and the lower range could be 30% deeper than the current 95th depth percentile. These constraints are consistent with depth shifts observed in the Mediterranean Sea on a time scale of 10 years for species with higher mobility (fish, cephalopods, malacostraca; Chaikin et al. 2022). We computed the Multivariate Environmental Similarity Surface (MESS; Elith et al. 2010) for each scenario to detect areas where model projections are outside the model's training range.

### 2.6 Species‐Level Distribution Metrics

For each species and scenario, we calculated distributional metrics based on the current and future range: the range area (RANGE, units: km^2^), its latitudinal centroid (LAT, units: °), and its average depth (DEPTH, units: m). To quantify fragmentation, based on the model's binary output, we first identified habitat clumps, defined as contiguous sets of suitable habitat cells. Then, we calculated the average edge‐to‐edge distance between clumps (package “gdistance”), and we calculated the average distance (FRAG, units: km). To assess projected changes, we calculated the relative variation of the range area with respect to the baseline scenario (ΔRANGE), and the absolute variation of the latitudinal centroid (ΔLAT), depth (ΔDEPTH), and fragmentation (ΔFRAG).

To identify drivers of changes in distributional metrics, we used the predictors' importance scores. We grouped species based on the most important predictor. Then, we checked to what extent the distribution metrics changed in the different groups. Next, we defined species‐level Ecological Indicator Values (EIVs; Descombes et al. 2020). We extracted the baseline predictors values at species occurrence points and calculated the median values, that is, EIV‐O2_median_, EIV‐CHL_median_, EIV‐T_median_, EIV‐T_min_, EIV‐SAL_median_, EIV‐Vm_median_, and EIV‐SAND. Additionally, we calculated the median depth (EIV‐DEPTH_median_), latitude (EIV‐LAT_median_), and fragmentation (EIV‐FRAG_median_). Then, we used Spearman's coefficient to identify correlations between EIVs and distributional metrics changes.

### 2.7 Community‐Level Diversity Metrics

Based on the single model's habitat suitability output, we calculated alpha diversity per each grid cell by stacking single species predictions and summing the habitat suitability scores (Calabrese et al. 2014). For each scenario, we calculated the geographic distribution of alpha diversity change (Δ*α*) as the difference between the alpha diversities in the future and the baseline for each grid cell. We calculated the geographic distribution of temporal beta diversity (*β*_tot_) by comparing current and future sets of species predicted to occur in each grid cell with the Jaccard index using the “divraster” package. We used the Podani decomposition of beta diversity to assess the turnover component (*β*_tu_), accounting for differences in species identities, and the nestedness component (*β*_ne_), accounting for species losses or gains (Carvalho et al. 2012; Podani and Schmera 2011). To synthesize in a single metric the contribution of species turnover and richness difference to beta diversity, we calculate *β*_ratio_ as the ratio between *β*_ne_ and *β*_tot_ (Albouy et al. 2012). Values of *β*_ratio_ close to one represent sites where the nestedness component prevails, while values close to zero represent sites where the turnover component prevails.

### 2.8 Hotspots Analysis

We used the Getis index (Getis and Ord 1992; “spdep” package) to identify spatial clusters of community change for alpha and beta diversity (hereafter: hotspots and “coldspots” of community change). We identified hotspots of α decrease (increase) as grid cells where the Getis index of Δ*α* is significantly low (high). We identified coldspots of community changes as grid cells where the Getis index of |Δ*α*| is significantly low, that is, sites with limited changes in number of species. Finally, we identified hotspots of turnover as grid cells where the Getis index of *β*_tu_ is significantly high. We did not include *β*_ne_ because of its high correlation with |Δ*α*|. We calculated the Getis index using 24 neighbors and the threshold *p* < 0.001 for significance.

## 3 Results

### 3.1 Models Results and Performances

The ensemble models for 339 species were fitted with generally good performances (Table S2, Figure S2), with an evaluation AUC = 0.86 [0.81, 0.90]; TSS = 0.62 [0.55, 0.70]; and BOYCE = 0.81 [0.68, 0.89] (the interval is the interquartile range: [25th percentile, 75th percentile]). The ensemble models showed limited overfitting, with limited drops of the metrics between calibration and evaluation (Figure S3): ΔAUC = −0.02 [−0.07, 0.01], ΔTSS = −0.01 [−0.09, 0.06], and ΔBOYCE = −0.15 [−0.27, −0.07].

The most important variables (Table S3) were the median temperature T_median_, 1 − *ρ* = 0.17 [0.07, 0.32], ranked as the most important predictor for 151 species; the median oxygen O2_median_, 1 − *ρ* = 0.12 [0.06, 0.26], first‐ranked for 98 species; the median salinity SAL_median_, 1 − *ρ* = 0.07 [0.04, 0.11], first‐ranked for 41 species; and the minimum temperature T_min_, 1 − *ρ* = 0.07 [0.02, 0.15], first‐ranked for 25 species. The MESS analysis showed that the areas where the models' projections are outside the training range are in the southern coast of the Mediterranean Sea only for 2080–2100 of the most extreme scenario RCP8.5 (Figure S5).

### 3.2 Species‐Level Distributional Changes

**FIGURE 2:**
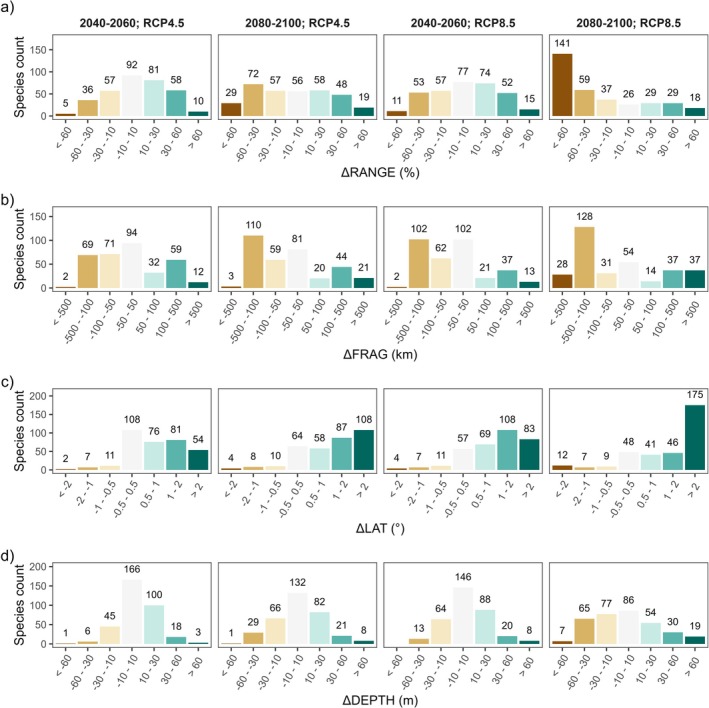
Frequency distribution of (a) ΔRANGE, units: %; (b) ΔFRAG, units: km; (c) ΔLAT, units: °; and (d) ΔDEPTH, units: M for the two future scenarios and the two time windows. The labels on top of the histograms are the number of species that fall within the histogram bin. The count of species does not sum up to the total number of modeled species because some species go extinct in some scenario and time window.

Species range change (Figure 2a) showed different trends for the analyzed scenarios: for RCP4.5, 149 species experienced range expansion greater than 10% in 2040–2060, and 158 species experienced range contraction greater than 10% in 2080–2100. For RCP8.5, 121 species experienced range contractions greater than 10% in 2040–2060, and 237 (i.e., 60% of species) experienced range contractions greater than 10% for 2080–2100, with 141 species experiencing range contractions larger than 60%.

Fragmentation (Figure 2b) showed a decrease in all scenarios, with most average distances between clumps being less than 500 km. Some species experienced increases in fragmentation: for RCP4.5, 71 and 65 species were projected to experience inter‐clump distances increases > 100 km for 2040–2060 and for 2080–2100, respectively. For RCP8.5, 50 and 74 species (i.e., 15% and 20% of species) were projected to experience the same increases in inter‐clump distances for 2040–2060 and for 2080–2100, respectively. Fragmentation was not necessarily related to range change, as species were evenly distributed between fragmentation and range change classes (Table 1). A high number of species (62–81, depending on the scenario and the time window) experienced both range contraction and fragmentation increase.

**TABLE 1:** Number of species that experiences range contraction (ΔRANGE < 0), range expansion (ΔRANGE > 0), extinction (ΔRANGE = −100), increase in fragmentation (ΔFRAG > 0), and decrease in fragmentation (ΔFRAG < 0) for the two scenarios and the two time windows.

Scenario	Time window	Range contraction, fragmentation decrease	Range contraction, fragmentation increase	Range expansion, fragmentation decrease	Range expansion, fragmentation increase	Extinction
RCP4.5	2040–2060	74	70	130	65	0
RCP4.5	2080–2100	118	72	100	47	2
RCP8.5	2040–2060	98	62	130	48	1
RCP8.5	2080–2100	136	81	63	26	33

Latitudinal shifts (Figure 2c) were projected to be positive for most species: for RCP4.5, the range centroid was projected to move northwards by more than 1° (i.e., more than approximately 100 km) for 135 species in 2040–2060 and for 195 species in 2080–2100. For RCP8.5, a northwards shift of more than 1° is projected for 191 species in 2040–2060 and for 221 species (i.e., 77% of species) in 2080–2100. In the last case, 175 species were projected to experience a northwards shift of more than 2°.

Depth shifts (Figure 2d) were projected to be positive for most species (i.e., depth centroid gets deeper): for RCP4.5, the average range depth increased by more than 10 m for 121 species in 2040–2060 and for 111 species in 2080–2100. For RCP8.5, the average range depth increased by more than 10 m for 116 species in 2040–2060, and 103 (i.e., 30% of species) in 2080–2100. In contrast, it is worth noting that in the last time window of RCP8.5, 72 species were projected to decrease their depth centroid by more than 30 m (i.e., they migrate to shallower waters).

### 3.3 Drivers of Distributional Changes

**FIGURE 3:**
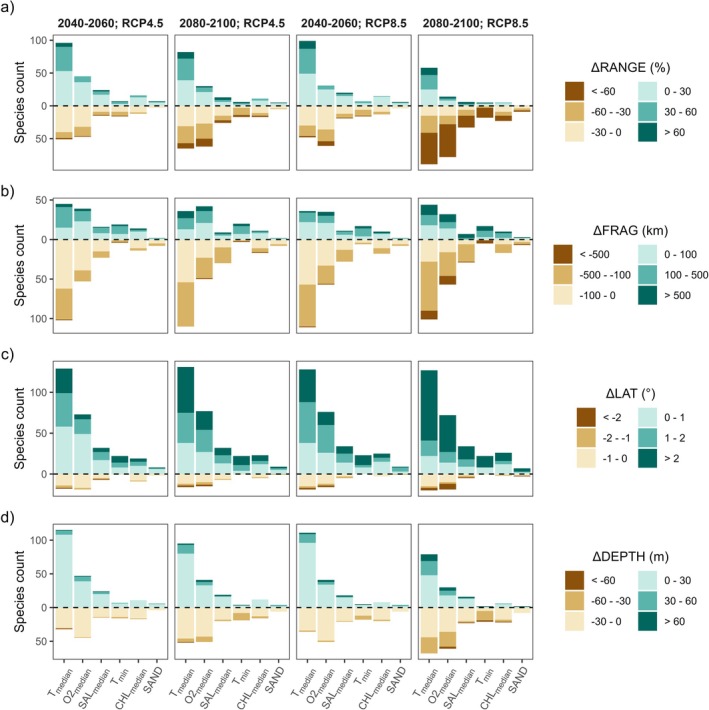
Drivers of (a) ΔRANGE, (b) ΔFRAG, (c) ΔLAT, and (d) ΔDEPTH for the two future scenarios and the two time windows. The color scale differs for the different metrics. Each species was labeled with the most important predictor, with the total height of each bar representing the number of species sharing the same most important predictor. The stacked colors represent the bins of metric changes. Since the variable importance is measured during model's calibration, the total height of the bars does not change across scenarios and time windows. The proportion of metrics changes varies across scenarios.

The range change bins were evenly distributed across first‐ranked predictors (Figure 3a). For 2040–2060 of both scenarios, species with the median temperature as first‐ranked predictor were more likely to experience a range expansion, while species with O2_median_ as first‐ranked predictor were more likely to experience a range contraction. The fragmentation change bins show that species with median temperature as first‐ranked predictor were more likely to experience a decrease in fragmentation, while species with oxygen at the seafloor as the first‐ranked predictor were more likely to experience an increase in fragmentation (Figure 3b). The depth bins show that species with median temperature as first‐ranked predictor were consistently more likely to experience northwards shifts across all scenarios and time windows (Figure 3c). The depth bins show that species with median temperature as first‐ranked predictor were consistently more likely to experience depth increases across all scenarios and time windows except RCP8.5, 2080–2100 (Figure 3d).

**FIGURE 4:**
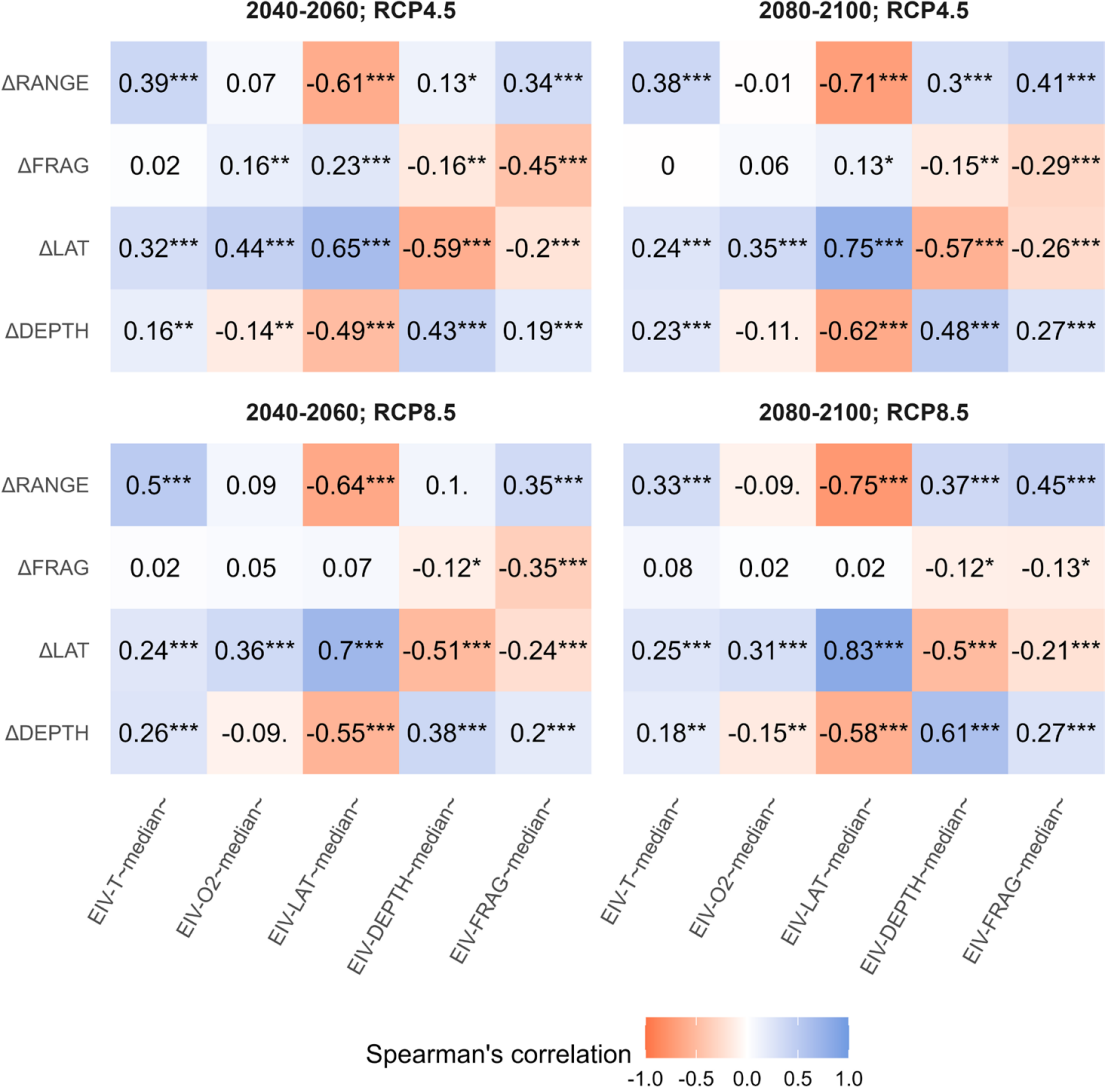
Spearman's correlation between distributional metrics (ΔRANGE, ΔFRAG, ΔLAT, ΔDEPTH) and Ecological Indicator Values (EIV‐T_median_, EIV‐O2_median_, EIV‐LAT_median_, EIV‐DEPTH_median_, EIV‐FRAG_median_). Only EIVs for the two top‐ranked predictors are reported. The stars represent the coefficient's significance, as measured by the *p*‐value; ***p < 0.001; **p < 0.01; *p < 0.05; ^.^ *p* < 0.1; no symbol: *p* > 0.1.

The distributional metrics were consistently correlated with EIVs across all scenarios and time windows (Figure 4). The range change (ΔRANGE) was negatively correlated with the median latitude of the current range (EIV‐LAT_median_) and positively correlated with the median temperature at occurrence points (EIV‐T_median_) and the current median inter‐clump distance (EIV‐FRAG_median_). The future change in fragmentation (ΔFRAG) was negatively correlated with the current median inter‐clump distance (EIV‐FRAG_median_). The latitudinal shift (ΔLAT) was negatively correlated with the current average range depth (EIV‐DEPTH_median_) and the current median inter‐clump distance (EIV‐FRAG_median_) and positively correlated with the current range latitude (EIV‐LAT_median_) and the median temperature (EIV‐T_median_) and oxygen (EIV‐O2_median_) at occurrence points. The depth shift was negatively correlated with range latitude (EIV‐LAT_median_) and positively correlated with current median range depth (EIV‐DEPTH_median_) and temperature (EIV‐T_median_) at occurrence points.

### 3.4 Community‐Level Diversity Metrics Changes

**FIGURE 5:**
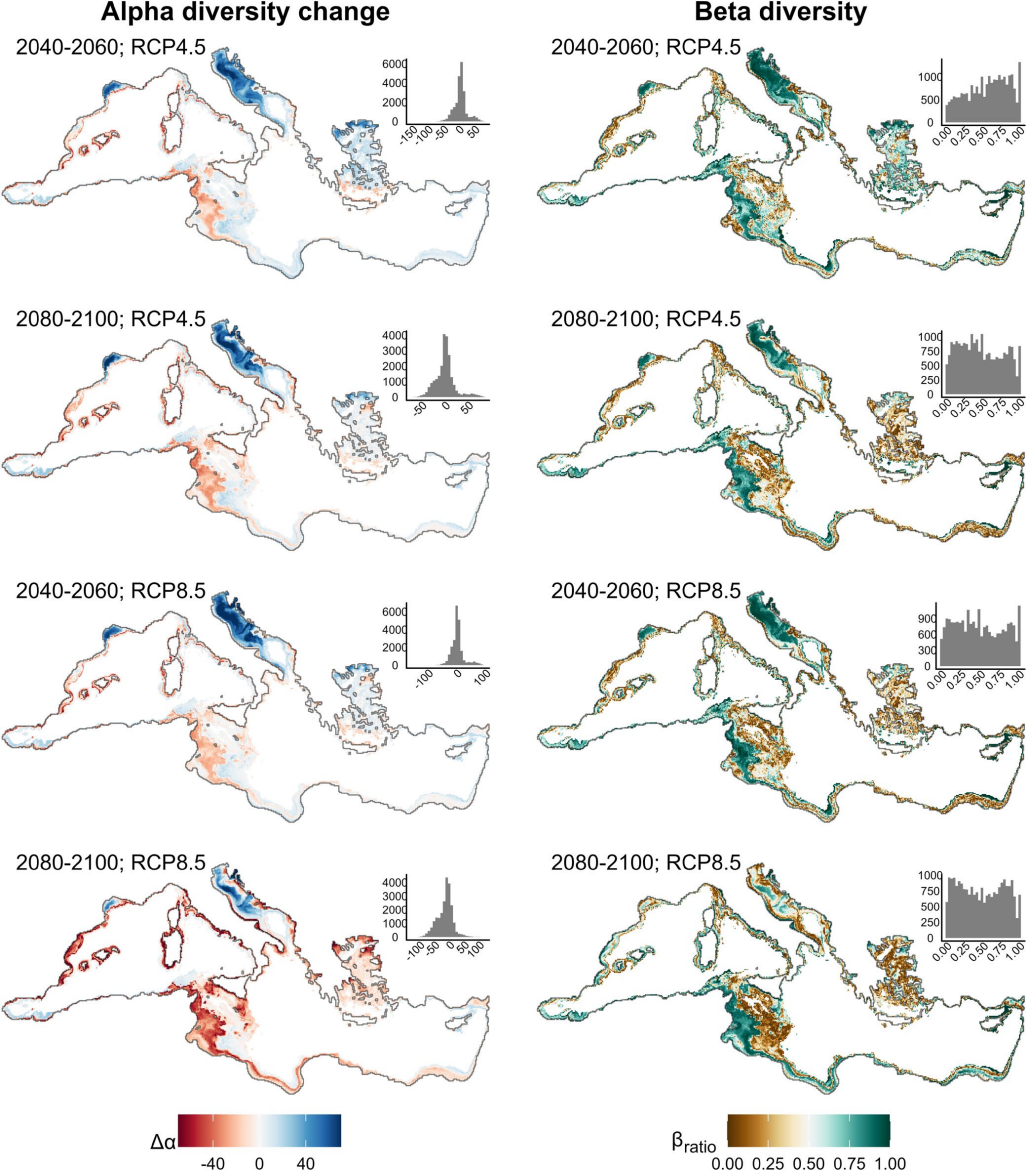
Maps of the change in alpha diversity (Δ*α*, first column) and beta diversity (*β*_ratio_, second column) for the two scenarios and the two time windows. Values of βratio close to one represent sites where the nestedness component prevails, while values close to zero represent sites where the turnover component prevails. The insert in the top‐right corner shows the frequency histogram of the mapped values. See Figure S6 for a detailed zoom on the inserts. See Figure S7 for the values of Δα and βratio averaged across subbasins and depth zones.

The stacked models showed an increase in alpha diversity (Δ*α*) in the northern Mediterranean Sea, particularly in the Adriatic Sea, the Northwestern Mediterranean, and the Northern Aegean Sea (Figure 5, Figure S7, Table S5). However, in the extreme 2080–2100 RCP8.5, the Northern Aegean and the coastal zone of the Adriatic Sea also showed a decrease in alpha diversity. The southern side of the Mediterranean and the Tyrrhenian Sea showed a consistent decrease in alpha diversity in all scenarios and time windows. The deeper parts of the Ionian Sea (depth > 200 m) showed no alpha diversity changes.

Beta diversity (measured by *β*_ratio_) showed a more heterogeneous response (Figure 5, Figure S7, Table S5). Nestedness (*β*_ratio_ values closer to one) prevailed in the shallow sections of the Northwestern Mediterranean and in the intermediate depths (40–200 m) of the Ionian Sea (Figure S7). Turnover (*β*_ratio_ values closer to zero) prevailed in the Tyrrhenian Sea and in the shallow and intermediate depths of the Levantine Sea. The Adriatic Sea showed mixed responses, with prevailing nestedness but high turnover in the coastal zones, which becomes predominant in 2080–2100 RCP8.5. The Aegean Sea also showed mixed responses, with no prevailing component in 2040–2060 RCP4.5 and prevailing turnover in all other scenarios and time windows.

**FIGURE 6:**
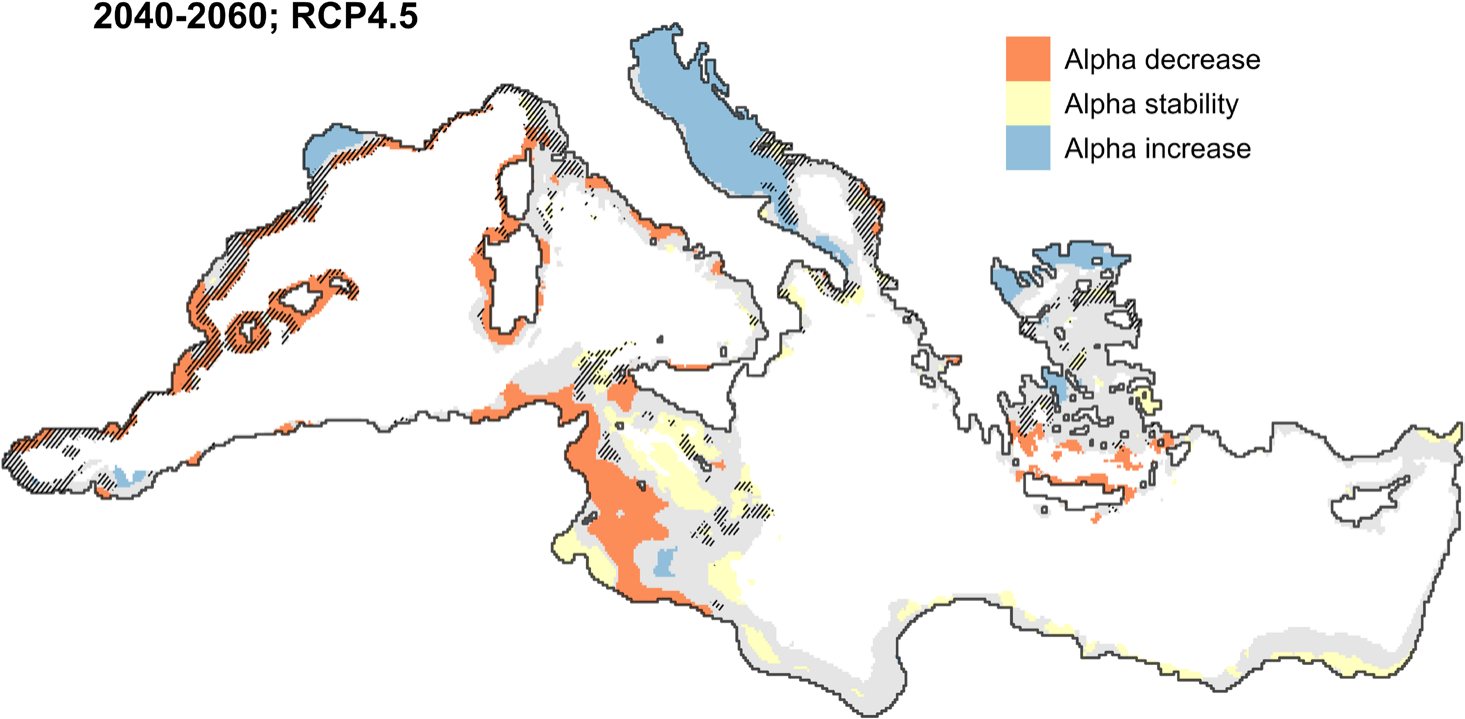
Spatial clusters of community change for the scenario RCP 4.5, time window 2040–2060. Clusters of community change were identified based on the Getis index, see Section 2.8. Blue, orange and yellow represent hot‐, cold‐ and stability‐spots of alpha diversity change, respectively. Dashed lines represent hotspots of species turnover. Gray‐shaded areas represents the fraction of the Mediterranean Sea with depth shallower than 800 m, where species distribution models were trained and projected. See Figures S8–S10 for the other time windows and scenarios.

The Getis analyses revealed significant overlaps between spatial clusters of alpha diversity changes and species turnover (Figure 6, Figures S8–S10). The identified “coldspots” of alpha change, located in the deeper sections of the Ionian Sea and in the Aegean Sea, were overlapping with hotspots of species turnover. On the contrary, the identified hotspots of alpha change, localized in the northern side of the Mediterranean (Adriatic Sea, Northern Aegean Sea, Northwestern Mediterranean Sea), showed limited overlap with hotspots of species turnover.

## 4 Discussion

### 4.1 Patterns of Species Redistribution

Our work highlights how climate change can reshape the geographic distribution of benthic species, with northward shifts in suitable habitats emerging as the most common pattern, followed by range contractions, depth migrations, and increasing habitat fragmentation (Figure 2). Sea water temperature and dissolved oxygen concentration near the seafloor, identified as the main drivers of these changes (Figure 3), are key parameters affecting benthic species physiology, including growth, reproduction, and survival (Diaz and Rosenberg 1995; Hiscock et al. 2004; Pinsky et al. 2020). Seawater temperature is a well‐known driver of distributional shifts in benthic fauna, with temperature‐related species displacements already observed in the North Sea (Hiddink et al. 2015). Rising temperatures can additionally increase the risk of mass mortality events associated with marine heatwaves (Carlot et al. 2025). Dissolved oxygen is also a key driver of species distribution, as benthic organisms are highly sensitive to deoxygenation (Vaquer‐Sunyer and Duarte 2008), especially when combined with rising temperatures (Vaquer‐Sunyer and Duarte 2008). Projected increases in seawater temperature and decreases in dissolved oxygen concentrations in the Mediterranean Sea (Reale et al. 2022) are therefore likely to explain the observed distributional shifts.

Our Ecological Indicator Values (EIVs) approach further highlighted the drivers of species redistribution. Cold‐adapted species (such as *Macropipus tuberculatus* , EIV‐T_median_ = 13.9°C, or *Galeodea rugosa*, EIV‐T_median_ = 13.8°C) were found to track shifting temperatures by moving northward, with continental barriers to dispersal causing substantial range contractions, identifying them as climate‐change losers. In contrast, warm‐adapted species (such as *Neverita josephinia*, EIV‐T_median_ = 17°C, or *Hermodice carunculate*, EIV‐T_median_ = 15.8°C) experience a northward expansion of their climatic suitable area, accompanied by northward range shifts and overall range growth, identifying them as climate‐change winners. This geographic pattern is consistent with similar studies focusing on coastal fish (Albouy et al. 2013; Ben Rais Lasram et al. 2010), but was not assessed yet with benthic organisms. Depth migrations are also explained by this temperature dependence, with cold‐adapted species moving towards deeper waters to escape warmer temperatures. The combination of northward and depth displacement will possibly open vacant ecological niches, increasing the risk of biological invasions in the southern and shallow area of the Mediterranean Sea. This pattern is already striking in the Levantine Mediterranean, where biological invasions of Lessespian species is progressing since several decades (Steger et al. 2024), with raising sea water temperatures being a key factor affecting the spread of such species (D'Amen et al. 2022).

Our analysis also shows that the interaction between fragmentation and range change is not straightforward, as the number of species that experience fragmentation is equally divided between species undergoing range expansion and range contraction. The assessment of future range changes is a key element in the assessment of species vulnerability to climate change (Pacifici et al. 2015), although fragmentation can also be related to extinction risks (Crooks et al. 2017). Species that undergo fragmentation reduction might lose genetic diversity due to increased genetic flow between previously isolated populations (Hellberg 2009). On the other hand, species that undergo fragmentation increase can be more vulnerable to disturbances, as a reduced likelihood of arrival of dispersal propagules in a disturbed site can impair population recovery (Weersing and Toonen 2009). Considering metrics representing the level of fragmentation of the suitable area can further support the assessment of species vulnerability as deemed, for instance, by the IUCN in criterion B for the Red List assessments.

### 4.2 Patterns of Community Change

Our work highlights how climate change affects Mediterranean benthic community composition. We show that the spatial patterns of change in species richness (alpha diversity) and composition (beta diversity) can be spatially disjunct (Figures 5 and 6). Hotspots of alpha diversity increase located in the Northern Mediterranean are also characterized by compositional nestedness, suggesting that future communities will host a larger number of species due to the northwards migration of warm‐adapted species (tropicalization). This pattern is partially hampered in the extreme scenario RCP8.5 for the 2080–2100 time window, when alpha diversity decrease is observed also in the Northern Aegean Sea and in the coastal regions of the Adriatic Sea, suggesting some extinction of cold‐adapted species will occur even in these refugia (deborealization). The “coldspots” of alpha change located in the Southern Aegean Sea and in the Central Mediterranean are characterized by compositional nestedness, with the future community being a subset of the current community due to the depletion of cold‐adapted species (deborelization), and the regional lack of species adapted to even warmer temperatures. On the contrary, communities located along the Spanish, French, and Italian coasts in the Western Mediterranean are hotspots of alpha change and show substantial overlap with hotspots of compositional turnover, suggesting the extinction/emigration of cold‐adapted species will not be compensated by immigration of warm‐adapted species. These sites will be particularly vulnerable because future communities will be composed of a lower number of substantially different species. The identified “coldspots” of alpha diversity change also show some overlap with hotspots of turnover, which becomes more evident in RCP8.5. Areas that might be considered stable just based on alpha diversity changes, such as deeper zones of the Sicilian strait, or the Aegen Sea, could partially overlap with hotspots of species turnover (Figure 6), becoming highly vulnerable because of the substantial community reorganization.

Community reorganization has relevant ecological consequences, such as changes in the carbon sink capacity, in bioturbation potential, and the overall system productivity due to the changes in the traits composition (Godbold and Solan 2013; Solan et al. 2004; Woodin et al. 2016). Biological communities are generally composed of species assemblages with redundant traits composition (Beauchard et al. 2023). Thus, the loss/gain of seafloor functionality may not be directly proportional to the number of species present in a site. However, with strong compositional changes, some traits combinations might be excluded, and cascading effects on seafloor functionality could be higher. Our approach to benthic species diversity supports the identification of vulnerable areas where risks of functionality losses are higher due to the losses of species (hotspots of alpha diversity loss highlighted in Figure 6) or due to community turnover (hotspots of turnover highlighted in Figure 6).

### 4.3 Models Limitations and Perspectives

Despite the contribution of our results to an understanding of benthic species redistribution, our study might be over‐optimistic regarding changes in species and biodiversity distribution as it could not account well for biotic interactions and species dispersal.

While it is known that biotic interactions can further reduce species range due to competitive exclusion or predation (Moullec et al. 2022), current methods that account for interaction mechanisms still face important limitations in their integrations and efficiency (Poggiato et al. 2021; Thuiller et al. 2024). In this regard, Joint Species Distribution Models can uncover residual patterns of co‐occurrence (Tikhonov et al. 2020), but our opportunistic occurrences data do not allow for a direct application of such methods. In our data, the geographic distribution of sampling bias might differ across species and this leads to incorrect estimates of species interactions (Romera‐Romera and Nieto‐Lugilde 2025). Future applications of Joint Species Distribution models for benthic fauna should focus on a subset of the analyzed data, for instance the MEDITS dataset, where sampling is standardized (Spedicato et al. 2019), at the cost of modeling a smaller number of species.

When modelling species distribution, accounting for dispersal shall be integrated when data is available (Chauvier‐Mendes et al. 2024; Shipley et al. 2022), in particular for species relying on passive dispersal for the recolonization of new habitats or slow dispersers not being able to track climate change, such as many benthic organisms (Hiddink et al. 2015). Our projections of future species distributions assume that “everything is everywhere” and species can colonize suitable habitats without dispersal limitations. Thus, the realized future species ranges are likely to be smaller due to dispersal limitation. The habitat suitability layers developed here could be used in a spatially explicit metacommunity model that includes dispersal limitation (Baldan et al. 2025) and connectivity mediated by currents (Andrello et al. 2025). This approach would support the identification of regions prone to species losses due to landscape fragmentation.

Despite the highlighted limitations, our stacked approach where species are modeled one‐at‐a‐time and without considering dispersal is useful as it supports the identification of the main drivers of distributional changes and the species that are at higher risk due to climate change.

### 4.4 Implications for Seafloor Management and Conservation Planning

Ecosystem‐based Marine Spatial Planning (MSP) is the process of allocating marine space to human activities to balance ecological, social and economic outcomes (Ehler and Douvere 2009), while maintaining marine ecosystems healthy, productive and resilient to sustain human uses of the ocean and provide goods and services (Foley et al. 2010). A climate‐smart MSP should also account for the effects of climate change on the structure and function of marine ecosystems, but its application is still limited (Frazão Santos et al. 2020; Queirós et al. 2021). Recent developments on climate‐informed MSP identify three categories of ecosystem response (Queirós et al. 2021): areas where future environmental conditions will remain close to current climatic boundaries (refugia), areas where high species turnover/vulnerability is projected to occur (hotspots), and areas where environmental conditions enhance ecosystem functions or resources availability (bright spots). Our work supports the identification of such responses. The identified “coldspots” of alpha diversity changes (but excluding the area that overlay with hotspots of species turnover) can be considered as refugia. Hotspots of alpha diversity decrease, and hotspots of turnover are particularly vulnerable due to the loss/change in species and the potential losses in functionality and ecosystem services. Hotspots of alpha diversity increase could be considered bright spots where new opportunities for exploiting biological resources might arise (although our results do not include commercially exploited species). Our results can support the implementation of several European policies, such as the Marine Strategy Framework Directive (2008/56/CE) which demands to map and characterize the seafloor integrity (descriptor D6), and the Nature Restoration Regulation (2024/1991) by supporting the identification of the areas suitable for the re‐establishment of specific habitat types.

Our results can also support biodiversity conservation on the regional scale. Marine Protected Areas (MPAs) were historically established focusing on local biodiversity attributes and implemented by regulating human activities to limit the anthropic impacts. Current trends in the MPAs planning process are moving towards the systematic identification of areas in need of protection based on the regional context (Brooks et al. 2006), to identify areas where the potential benefits of protection measures are higher (Zhao et al. 2020), a paradigm shift required by current policies setting targets as specific shares of area. For instance, the third objective outlined in the 2022 Kunming‐Montreal agreement sets the target of 30% of marine areas under protection by 2030. As MPAs planned to protect current biodiversity might not be as efficient in the future, when biodiversity and functionality hotspots might be located elsewhere (Irvine et al. 2025; Queirós et al. 2021), the planning process must account for both current and future conditions (Doxa et al. 2022; Wilson et al. 2020) and propose specific strategies depending on the expected biodiversity change (Queirós et al. 2021). The areas we identified as hotspots of community turnover might not benefit from classic MPA planning based on prioritizing species‐rich sites, or sites characterized by the presence of target species, because optimal sites with favorable environmental conditions in the future might be located outside the designated MPA. Rather, the planning process might focus on connectivity criteria to accommodate the shifting species ranges and to ensure that species do not encounter dispersal barriers while migrating to suitable areas (Roberts et al. 2017). On the other hand, the planning of MPAs in “coldspots” should focus on sites that are characterized by high alpha diversity to maximize the number of species protected per unit of area protected (McLeod et al. 2009). In this case, larger MPAs could be more effective in preserving current and future biodiversity (Edgar et al. 2014). MPAs located in hotspots of alpha diversity increase might be prone to biological invasions because new, empty ecological niches might be available.

## 5 Conclusions

The geography of benthic community change in the Mediterranean will exhibit substantial rearrangement under climate change, due to species experiencing range contraction, moving northward, moving towards deeper waters, and experiencing range fragmentation. The resulting hotspots of species losses, gains, stability, and turnover can inform a climate‐smart, ecosystem‐based Marine Spatial Planning. Prioritizing connectivity in the identified community turnover hotspots and extending protected areas in regions with stable communities and limited turnover is recommended for an improved planning of Marine Protected Areas.

## Author Contributions

**Damiano Baldan:** conceptualization, data curation, methodology, investigation, visualization, writing – original draft, writing – review and editing. **Yohann Chauvier‐Mendes:** methodology, writing – review and editing. **Diego Panzeri:** writing – review and editing. **Gianpiero Cossarini:** funding acquisition, writing – review and editing. **Cosimo Solidoro:** funding acquisition, writing – review and editing. **Vinko Bandelj:** conceptualization, methodology, funding acquisition, writing – review and editing.

## Conflicts of Interest

The authors declare no conflicts of interest.

## Supporting information


**Data S1:** gcb70725‐sup‐0001‐Supinfo.docx.

## Data Availability

The data generated in this paper and the codes used are available in Zenodo at: https://doi.org/10.5281/zenodo.17804289.
