# Supplementary material for: The Geography of Mediterranean Benthic Communities Under Climate Change
Source: Glob Chang Biol. 2026 Jan 29;32(2):e70725. doi: 10.1111/gcb.70725 (PMC12853245; doi:10.1111/gcb.70725)
Supplement: Supplementary file 1 — Data S1: gcb70725‐sup‐0001‐Supinfo.docx. [file GCB-32-e70725-s001.docx]

**Supplementary material for:**

**The geography of Mediterranean benthic communities under climate change**

**Table S1** – List of species used to fit the models. The field AphiaID refers to the Aphia code from the Darwin Core Standard. The field npoints is the number of points used to fit the models

| **ID** | **Phylum** | **Class** | **Order** | **Family** | **Name** | **AphiaID** | **npoints** |
| --- | --- | --- | --- | --- | --- | --- | --- |
| 1 | Annelida | Polychaeta |  | Capitellidae | *Heteromastus filiformis* | 129884 | 153 |
| 2 | Annelida | Polychaeta |  | Capitellidae | *Notomastus latericeus* | 129898 | 180 |
| 3 | Annelida | Polychaeta |  | Oweniidae | *Myriochele heeri* | 130542 | 124 |
| 4 | Annelida | Polychaeta |  | Oweniidae | *Owenia fusiformis* | 130544 | 177 |
| 5 | Annelida | Polychaeta | Amphinomida | Amphinomidae | *Hermodice carunculata* | 129831 | 279 |
| 6 | Annelida | Polychaeta | Echiuroidea | Bonelliidae | *Bonellia viridis* | 110363 | 272 |
| 7 | Annelida | Polychaeta | Eunicida | Eunicidae | *Eunice vittata* | 130067 | 164 |
| 8 | Annelida | Polychaeta | Eunicida | Lumbrineridae | *Lumbrineris latreilli* | 130248 | 283 |
| 9 | Annelida | Polychaeta | Eunicida | Oenonidae | *Drilonereis filum* | 129856 | 137 |
| 10 | Annelida | Polychaeta | Eunicida | Onuphidae | *Aponuphis bilineata* | 130452 | 123 |
| 11 | Annelida | Polychaeta | Eunicida | Onuphidae | *Hyalinoecia tubicola* | 130464 | 231 |
| 12 | Annelida | Polychaeta | Phyllodocida | Aphroditidae | *Aphrodita aculeata* | 129840 | 487 |
| 13 | Annelida | Polychaeta | Phyllodocida | Aphroditidae | *Laetmonice hystrix* | 129845 | 137 |
| 14 | Annelida | Polychaeta | Phyllodocida | Glyceridae | *Glycera alba* | 130116 | 157 |
| 15 | Annelida | Polychaeta | Phyllodocida | Glyceridae | *Glycera tridactyla* | 130130 | 122 |
| 16 | Annelida | Polychaeta | Phyllodocida | Glyceridae | *Glycera unicornis* | 130131 | 195 |
| 17 | Annelida | Polychaeta | Phyllodocida | Goniadidae | *Goniada maculata* | 130140 | 201 |
| 18 | Annelida | Polychaeta | Phyllodocida | Nephtyidae | *Nephtys hombergii* | 130359 | 144 |
| 19 | Annelida | Polychaeta | Phyllodocida | Sigalionidae | *Sigalion mathildae* | 131072 | 122 |
| 20 | Annelida | Polychaeta | Sabellida | Sabellidae | *Sabella pavonina* | 130967 | 221 |
| 21 | Annelida | Polychaeta | Sabellida | Sabellidae | *Sabella spallanzanii* | 130969 | 478 |
| 22 | Annelida | Polychaeta | Sabellida | Serpulidae | *Protula intestinum* | 131032 | 175 |
| 23 | Annelida | Polychaeta | Sabellida | Serpulidae | *Protula tubularia* | 131035 | 364 |
| 24 | Annelida | Polychaeta | Sabellida | Serpulidae | *Serpula vermicularis* | 131051 | 287 |
| 25 | Annelida | Polychaeta | Spionida | Spionidae | *Laonice cirrata* | 131128 | 127 |
| 26 | Annelida | Polychaeta | Spionida | Spionidae | *Prionospio steenstrupi* | 131164 | 135 |
| 27 | Annelida | Polychaeta | Terebellida | Ampharetidae | *Ampharete grubei* | 152272 | 132 |
| 28 | Annelida | Polychaeta | Terebellida | Melinnidae | *Melinna palmata* | 129808 | 148 |
| 29 | Annelida | Polychaeta | Terebellida | Sternaspidae | *Sternaspis scutata* | 131242 | 258 |
| 30 | Annelida | Polychaeta | Terebellida | Terebellidae | *Eupolymnia nebulosa* | 131489 | 173 |
| 31 | Arthropoda | Malacostraca | Amphipoda | Ampeliscidae | *Ampelisca sarsi* | 101923 | 137 |
| 32 | Arthropoda | Malacostraca | Amphipoda | Ampeliscidae | *Ampelisca typica* | 101933 | 156 |
| 33 | Arthropoda | Malacostraca | Decapoda | Alpheidae | *Alpheus glaber* | 107477 | 929 |
| 34 | Arthropoda | Malacostraca | Decapoda | Alpheidae | *Athanas nitescens* | 107486 | 155 |
| 35 | Arthropoda | Malacostraca | Decapoda | Axiidae | *Calocaris macandreae* | 107726 | 276 |
| 36 | Arthropoda | Malacostraca | Decapoda | Calappidae | *Calappa granulata* | 107268 | 504 |
| 37 | Arthropoda | Malacostraca | Decapoda | Cambaridae | *Procambarus clarkii* | 465540 | 148 |
| 38 | Arthropoda | Malacostraca | Decapoda | Carcinidae | *Carcinus aestuarii* | 107380 | 219 |
| 39 | Arthropoda | Malacostraca | Decapoda | Crangonidae | *Aegaeon cataphractus* | 107548 | 442 |
| 40 | Arthropoda | Malacostraca | Decapoda | Crangonidae | *Aegaeon lacazei* | 107549 | 757 |
| 41 | Arthropoda | Malacostraca | Decapoda | Crangonidae | *Philocheras echinulatus* | 107558 | 183 |
| 42 | Arthropoda | Malacostraca | Decapoda | Crangonidae | *Pontophilus spinosus* | 107564 | 600 |
| 43 | Arthropoda | Malacostraca | Decapoda | Diogenidae | *Calcinus tubularis* | 107194 | 206 |
| 44 | Arthropoda | Malacostraca | Decapoda | Diogenidae | *Clibanarius erythropus* | 107196 | 547 |
| 45 | Arthropoda | Malacostraca | Decapoda | Diogenidae | *Dardanus arrosor* | 107197 | 1664 |
| 46 | Arthropoda | Malacostraca | Decapoda | Diogenidae | *Dardanus calidus* | 107198 | 373 |
| 47 | Arthropoda | Malacostraca | Decapoda | Diogenidae | *Diogenes pugilator* | 107199 | 323 |
| 48 | Arthropoda | Malacostraca | Decapoda | Diogenidae | *Paguristes eremita* | 107200 | 170 |
| 49 | Arthropoda | Malacostraca | Decapoda | Dorippidae | *Medorippe lanata* | 107288 | 882 |
| 50 | Arthropoda | Malacostraca | Decapoda | Dromiidae | *Dromia personata* | 107258 | 301 |
| 51 | Arthropoda | Malacostraca | Decapoda | Epialtidae | *Pisa armata* | 107353 | 219 |
| 52 | Arthropoda | Malacostraca | Decapoda | Eriphiidae | *Eriphia verrucosa* | 107409 | 703 |
| 53 | Arthropoda | Malacostraca | Decapoda | Ethusidae | *Ethusa mascarone* | 107283 | 136 |
| 54 | Arthropoda | Malacostraca | Decapoda | Galatheidae | *Galathea intermedia* | 107150 | 171 |
| 55 | Arthropoda | Malacostraca | Decapoda | Galatheidae | *Galathea strigosa* | 107155 | 186 |
| 56 | Arthropoda | Malacostraca | Decapoda | Geryonidae | *Geryon longipes* | 107373 | 789 |
| 57 | Arthropoda | Malacostraca | Decapoda | Goneplacidae | *Goneplax rhomboides* | 107292 | 1325 |
| 58 | Arthropoda | Malacostraca | Decapoda | Grapsidae | *Pachygrapsus marmoratus* | 107455 | 1001 |
| 59 | Arthropoda | Malacostraca | Decapoda | Homolidae | *Homola barbata* | 107262 | 180 |
| 60 | Arthropoda | Malacostraca | Decapoda | Homolidae | *Paromola cuvieri* | 107264 | 689 |
| 61 | Arthropoda | Malacostraca | Decapoda | Inachidae | *Inachus communissimus* | 107326 | 127 |
| 62 | Arthropoda | Malacostraca | Decapoda | Inachidae | *Inachus dorsettensis* | 107327 | 432 |
| 63 | Arthropoda | Malacostraca | Decapoda | Inachidae | *Inachus phalangium* | 107333 | 145 |
| 64 | Arthropoda | Malacostraca | Decapoda | Inachidae | *Inachus thoracicus* | 107334 | 221 |
| 65 | Arthropoda | Malacostraca | Decapoda | Inachidae | *Macropodia linaresi* | 107341 | 121 |
| 66 | Arthropoda | Malacostraca | Decapoda | Inachidae | *Macropodia longirostris* | 107343 | 126 |
| 67 | Arthropoda | Malacostraca | Decapoda | Inachidae | *Macropodia rostrata* | 107345 | 266 |
| 68 | Arthropoda | Malacostraca | Decapoda | Inachidae | *Macropodia tenuirostris* | 107346 | 910 |
| 69 | Arthropoda | Malacostraca | Decapoda | Latreilliidae | *Latreillia elegans* | 107265 | 252 |
| 70 | Arthropoda | Malacostraca | Decapoda | Lysmatidae | *Lysmata seticaudata* | 107528 | 152 |
| 71 | Arthropoda | Malacostraca | Decapoda | Majidae | *Maja crispata* | 107348 | 371 |
| 72 | Arthropoda | Malacostraca | Decapoda | Majidae | *Maja squinado* | 107350 | 321 |
| 73 | Arthropoda | Malacostraca | Decapoda | Munididae | *Munida intermedia* | 107157 | 807 |
| 74 | Arthropoda | Malacostraca | Decapoda | Munididae | *Munida tenuimana* | 107166 | 247 |
| 75 | Arthropoda | Malacostraca | Decapoda | Munididae | *Iridonida speciosa* | 1606714 | 512 |
| 76 | Arthropoda | Malacostraca | Decapoda | Nephropidae | *Homarus gammarus* | 107253 | 189 |
| 77 | Arthropoda | Malacostraca | Decapoda | Paguridae | *Pagurus alatus* | 107230 | 730 |
| 78 | Arthropoda | Malacostraca | Decapoda | Paguridae | *Pagurus anachoretus* | 107231 | 306 |
| 79 | Arthropoda | Malacostraca | Decapoda | Paguridae | *Pagurus cuanensis* | 107235 | 264 |
| 80 | Arthropoda | Malacostraca | Decapoda | Paguridae | *Pagurus excavatus* | 107236 | 750 |
| 81 | Arthropoda | Malacostraca | Decapoda | Paguridae | *Pagurus prideaux* | 107239 | 757 |
| 82 | Arthropoda | Malacostraca | Decapoda | Palaemonidae | *Palaemon elegans* | 107614 | 405 |
| 83 | Arthropoda | Malacostraca | Decapoda | Palaemonidae | *Palaemon serratus* | 107616 | 168 |
| 84 | Arthropoda | Malacostraca | Decapoda | Palinuridae | *Palinurus elephas* | 107703 | 756 |
| 85 | Arthropoda | Malacostraca | Decapoda | Pandalidae | *Chlorotocus crassicornis* | 107642 | 1326 |
| 86 | Arthropoda | Malacostraca | Decapoda | Pandalidae | *Plesionika acanthonotus* | 107654 | 881 |
| 87 | Arthropoda | Malacostraca | Decapoda | Pandalidae | *Plesionika antigai* | 107655 | 604 |
| 88 | Arthropoda | Malacostraca | Decapoda | Pandalidae | *Plesionika edwardsii* | 107656 | 645 |
| 89 | Arthropoda | Malacostraca | Decapoda | Pandalidae | *Plesionika gigliolii* | 107659 | 1099 |
| 90 | Arthropoda | Malacostraca | Decapoda | Pandalidae | *Plesionika heterocarpus* | 107660 | 1598 |
| 91 | Arthropoda | Malacostraca | Decapoda | Pandalidae | *Plesionika martia* | 107661 | 1650 |
| 92 | Arthropoda | Malacostraca | Decapoda | Pandalidae | *Plesionika narval* | 107662 | 272 |
| 93 | Arthropoda | Malacostraca | Decapoda | Parthenopidae | *Spinolambrus macrochelos* | 442337 | 266 |
| 94 | Arthropoda | Malacostraca | Decapoda | Penaeidae | *Penaeus kerathurus* | 246388 | 178 |
| 95 | Arthropoda | Malacostraca | Decapoda | Pilumnidae | *Pilumnus hirtellus* | 107418 | 167 |
| 96 | Arthropoda | Malacostraca | Decapoda | Pilumnidae | *Pilumnus spinifer* | 107420 | 269 |
| 97 | Arthropoda | Malacostraca | Decapoda | Pilumnidae | *Pilumnus villosissimus* | 107421 | 132 |
| 98 | Arthropoda | Malacostraca | Decapoda | Polybiidae | *Bathynectes maravigna* | 107377 | 494 |
| 99 | Arthropoda | Malacostraca | Decapoda | Polybiidae | *Polybius vernalis* | 1750288 | 151 |
| 100 | Arthropoda | Malacostraca | Decapoda | Polybiidae | *Polybius depurator* | 1750291 | 1953 |
| 101 | Arthropoda | Malacostraca | Decapoda | Polybiidae | *Macropipus tuberculatus* | 107397 | 1528 |
| 102 | Arthropoda | Malacostraca | Decapoda | Polychelidae | *Polycheles typhlops* | 107696 | 1371 |
| 103 | Arthropoda | Malacostraca | Decapoda | Porcellanidae | *Pisidia longicornis* | 107188 | 130 |
| 104 | Arthropoda | Malacostraca | Decapoda | Processidae | *Processa canaliculata* | 107682 | 751 |
| 105 | Arthropoda | Malacostraca | Decapoda | Processidae | *Processa nouveli* | 107689 | 247 |
| 106 | Arthropoda | Malacostraca | Decapoda | Scyllaridae | *Scyllarides latus* | 107708 | 179 |
| 107 | Arthropoda | Malacostraca | Decapoda | Scyllaridae | *Scyllarus arctus* | 107709 | 185 |
| 108 | Arthropoda | Malacostraca | Decapoda | Solenoceridae | *Solenocera membranacea* | 107120 | 1614 |
| 109 | Arthropoda | Malacostraca | Decapoda | Stenopodidae | *Stenopus spinosus* | 107721 | 135 |
| 110 | Arthropoda | Malacostraca | Decapoda | Xanthidae | *Monodaeus couchii* | 241154 | 549 |
| 111 | Arthropoda | Malacostraca | Decapoda | Xanthidae | *Xantho poressa* | 107442 | 138 |
| 112 | Arthropoda | Malacostraca | Isopoda | Cirolanidae | *Natatolana borealis* | 118859 | 128 |
| 113 | Arthropoda | Malacostraca | Stomatopoda | Squillidae | *Rissoides pallidus* | 136136 | 201 |
| 114 | Arthropoda | Malacostraca | Stomatopoda | Squillidae | *Squilla mantis* | 136137 | 1074 |
| 115 | Arthropoda | Malacostraca | Tanaidacea | Apseudidae | *Apseudopsis latreillii* | 247077 | 135 |
| 116 | Arthropoda | Thecostraca | Scalpellomorpha | Scalpellidae | *Scalpellum scalpellum* | 106204 | 321 |
| 117 | Brachiopoda | Rhynchonellata | Terebratulida | Terebratulidae | *Gryphus vitreus* | 104068 | 344 |
| 118 | Bryozoa | Gymnolaemata | Cheilostomatida | Adeonidae | *Adeonella calveti* | 111055 | 144 |
| 119 | Bryozoa | Gymnolaemata | Cheilostomatida | Adeonidae | *Reptadeonella violacea* | 111061 | 132 |
| 120 | Bryozoa | Gymnolaemata | Cheilostomatida | Bitectiporidae | *Pentapora fascialis* | 111082 | 253 |
| 121 | Bryozoa | Gymnolaemata | Cheilostomatida | Bitectiporidae | *Schizomavella (Schizomavella) mamillata* | 862797 | 129 |
| 122 | Bryozoa | Gymnolaemata | Cheilostomatida | Electridae | *Electra posidoniae* | 111356 | 155 |
| 123 | Bryozoa | Gymnolaemata | Cheilostomatida | Myriaporidae | *Myriapora truncata* | 111435 | 390 |
| 124 | Bryozoa | Gymnolaemata | Cheilostomatida | Phidoloporidae | *Reteporella grimaldii* | 111453 | 222 |
| 125 | Bryozoa | Gymnolaemata | Cheilostomatida | Phidoloporidae | *Reteporella mediterranea* | 1041728 | 123 |
| 126 | Bryozoa | Gymnolaemata | Cheilostomatida | Schizoporellidae | *Schizobrachiella sanguinea* | 111522 | 170 |
| 127 | Bryozoa | Gymnolaemata | Cheilostomatida | Smittinidae | *Smittina cervicornis* | 111551 | 180 |
| 128 | Chordata | Ascidiacea | Aplousobranchia | Clavelinidae | *Clavelina lepadiformis* | 103552 | 190 |
| 129 | Chordata | Ascidiacea | Aplousobranchia | Diazonidae | *Diazona violacea* | 103733 | 311 |
| 130 | Chordata | Ascidiacea | Aplousobranchia | Polyclinidae | *Aplidium conicum* | 103641 | 227 |
| 131 | Chordata | Ascidiacea | Aplousobranchia | Polyclinidae | *Aplidium elegans* | 103643 | 127 |
| 132 | Chordata | Ascidiacea | Phlebobranchia | Ascidiidae | *Ascidia mentula* | 103710 | 656 |
| 133 | Chordata | Ascidiacea | Phlebobranchia | Ascidiidae | *Ascidia virginea* | 103717 | 267 |
| 134 | Chordata | Ascidiacea | Phlebobranchia | Ascidiidae | *Ascidiella aspersa* | 103718 | 136 |
| 135 | Chordata | Ascidiacea | Phlebobranchia | Ascidiidae | *Ascidiella scabra* | 103719 | 172 |
| 136 | Chordata | Ascidiacea | Phlebobranchia | Ascidiidae | *Phallusia mammillata* | 103724 | 740 |
| 137 | Chordata | Ascidiacea | Phlebobranchia | Cionidae | *Ciona intestinalis* | 103732 | 163 |
| 138 | Chordata | Ascidiacea | Stolidobranchia | Molgulidae | *Molgula appendiculata* | 103771 | 144 |
| 139 | Chordata | Ascidiacea | Stolidobranchia | Pyuridae | *Halocynthia papillosa* | 103827 | 551 |
| 140 | Chordata | Ascidiacea | Stolidobranchia | Pyuridae | *Microcosmus sabatieri* | 103844 | 231 |
| 141 | Chordata | Ascidiacea | Stolidobranchia | Pyuridae | *Microcosmus vulgaris* | 103846 | 351 |
| 142 | Chordata | Ascidiacea | Stolidobranchia | Styelidae | *Botryllus schlosseri* | 103862 | 278 |
| 143 | Cnidaria | Hexacorallia | Actiniaria | Actiniidae | *Actinia mediterranea* | 854459 | 545 |
| 144 | Cnidaria | Hexacorallia | Actiniaria | Actiniidae | *Anemonia viridis* | 100808 | 587 |
| 145 | Cnidaria | Hexacorallia | Actiniaria | Actiniidae | *Cribrinopsis crassa* | 100823 | 167 |
| 146 | Cnidaria | Hexacorallia | Actiniaria | Aiptasiidae | *Aiptasia mutabilis* | 100859 | 389 |
| 147 | Cnidaria | Hexacorallia | Actiniaria | Hormathiidae | *Actinauge richardi* | 100930 | 303 |
| 148 | Cnidaria | Hexacorallia | Actiniaria | Hormathiidae | *Calliactis palliata* | 1658483 | 345 |
| 149 | Cnidaria | Hexacorallia | Actiniaria | Hormathiidae | *Calliactis parasitica* | 100946 | 738 |
| 150 | Cnidaria | Hexacorallia | Actiniaria | Sagartiidae | *Cereus pedunculatus* | 100987 | 189 |
| 151 | Cnidaria | Hexacorallia | Ceriantharia | Cerianthidae | *Cerianthus membranaceus* | 101011 | 218 |
| 152 | Cnidaria | Hexacorallia | Ceriantharia | Cerianthidae | *Cerianthus membranacea* | 856035 | 169 |
| 153 | Cnidaria | Hexacorallia | Scleractinia | Caryophylliidae | *Caryophyllia (Caryophyllia) inornata* | 135141 | 140 |
| 154 | Cnidaria | Hexacorallia | Scleractinia | Caryophylliidae | *Caryophyllia (Caryophyllia) smithii* | 135144 | 141 |
| 155 | Cnidaria | Hexacorallia | Scleractinia | Cladocoridae | *Cladocora caespitosa* | 135146 | 598 |
| 156 | Cnidaria | Hexacorallia | Scleractinia | Dendrophylliidae | *Astroides calycularis* | 135178 | 131 |
| 157 | Cnidaria | Hexacorallia | Scleractinia | Dendrophylliidae | *Balanophyllia (Balanophyllia) europaea* | 135180 | 436 |
| 158 | Cnidaria | Hexacorallia | Scleractinia | Dendrophylliidae | *Leptopsammia pruvoti* | 135193 | 334 |
| 159 | Cnidaria | Hexacorallia | Scleractinia | Oculinidae | *Oculina patagonica* | 135210 | 130 |
| 160 | Cnidaria | Hexacorallia | Zoantharia | Parazoanthidae | *Parazoanthus axinellae* | 101055 | 498 |
| 161 | Cnidaria | Hydrozoa | Leptothecata | Aglaopheniidae | *Lytocarpia myriophyllum* | 117302 | 361 |
| 162 | Cnidaria | Hydrozoa | Leptothecata | Plumulariidae | *Nemertesia antennina* | 117809 | 296 |
| 163 | Cnidaria | Hydrozoa | Leptothecata | Plumulariidae | *Nemertesia ramosa* | 117815 | 248 |
| 164 | Cnidaria | Octocorallia | Malacalcyonacea | Acanthogorgiidae | *Paramuricea clavata* | 125387 | 443 |
| 165 | Cnidaria | Octocorallia | Malacalcyonacea | Alcyoniidae | *Alcyonium acaule* | 125331 | 197 |
| 166 | Cnidaria | Octocorallia | Malacalcyonacea | Alcyoniidae | *Alcyonium coralloides* | 125332 | 148 |
| 167 | Cnidaria | Octocorallia | Malacalcyonacea | Alcyoniidae | *Alcyonium palmatum* | 125334 | 1175 |
| 168 | Cnidaria | Octocorallia | Malacalcyonacea | Eunicellidae | *Eunicella cavolini* | 125361 | 454 |
| 169 | Cnidaria | Octocorallia | Malacalcyonacea | Eunicellidae | *Eunicella singularis* | 125365 | 426 |
| 170 | Cnidaria | Octocorallia | Malacalcyonacea | Eunicellidae | *Eunicella verrucosa* | 125366 | 208 |
| 171 | Cnidaria | Octocorallia | Malacalcyonacea | Gorgoniidae | *Leptogorgia sarmentosa* | 125369 | 223 |
| 172 | Cnidaria | Octocorallia | Scleralcyonacea | Coralliidae | *Corallium rubrum* | 125416 | 479 |
| 173 | Cnidaria | Octocorallia | Scleralcyonacea | Funiculinidae | *Funiculina quadrangularis* | 128506 | 719 |
| 174 | Cnidaria | Octocorallia | Scleralcyonacea | Keratoisididae | *Isidella elongata* | 125373 | 252 |
| 175 | Cnidaria | Octocorallia | Scleralcyonacea | Pennatulidae | *Pennatula phosphorea* | 128517 | 284 |
| 176 | Cnidaria | Octocorallia | Scleralcyonacea | Pennatulidae | *Pennatula rubra* | 128519 | 505 |
| 177 | Cnidaria | Octocorallia | Scleralcyonacea | Pennatulidae | *Pteroeides griseum* | 181504 | 365 |
| 178 | Cnidaria | Octocorallia | Scleralcyonacea | Veretillidae | *Veretillum cynomorium* | 128536 | 244 |
| 179 | Echinodermata | Asteroidea | Forcipulatida | Asteriidae | *Coscinasterias tenuispina* | 123795 | 344 |
| 180 | Echinodermata | Asteroidea | Forcipulatida | Asteriidae | *Marthasterias glacialis* | 123803 | 733 |
| 181 | Echinodermata | Asteroidea | Paxillosida | Astropectinidae | *Astropecten aranciacus* | 123856 | 702 |
| 182 | Echinodermata | Asteroidea | Paxillosida | Astropectinidae | *Astropecten bispinosus* | 123859 | 344 |
| 183 | Echinodermata | Asteroidea | Paxillosida | Astropectinidae | *Astropecten irregularis* | 123867 | 224 |
| 184 | Echinodermata | Asteroidea | Paxillosida | Astropectinidae | *Astropecten jonstoni* | 123868 | 273 |
| 185 | Echinodermata | Asteroidea | Paxillosida | Astropectinidae | *Astropecten platyacanthus* | 123876 | 122 |
| 186 | Echinodermata | Asteroidea | Paxillosida | Astropectinidae | *Astropecten spinulosus* | 123878 | 218 |
| 187 | Echinodermata | Asteroidea | Paxillosida | Astropectinidae | *Tethyaster subinermis* | 123913 | 529 |
| 188 | Echinodermata | Asteroidea | Paxillosida | Luidiidae | *Luidia ciliaris* | 123920 | 309 |
| 189 | Echinodermata | Asteroidea | Paxillosida | Luidiidae | *Luidia sarsii* | 123922 | 155 |
| 190 | Echinodermata | Asteroidea | Spinulosida | Echinasteridae | *Echinaster (Echinaster) sepositus* | 125161 | 1239 |
| 191 | Echinodermata | Asteroidea | Valvatida | Asterinidae | *Anseropoda placenta* | 123985 | 367 |
| 192 | Echinodermata | Asteroidea | Valvatida | Chaetasteridae | *Chaetaster longipes* | 124004 | 344 |
| 193 | Echinodermata | Asteroidea | Valvatida | Goniasteridae | *Peltaster placenta* | 124055 | 150 |
| 194 | Echinodermata | Asteroidea | Valvatida | Ophidiasteridae | *Hacelia attenuata* | 124094 | 351 |
| 195 | Echinodermata | Asteroidea | Valvatida | Ophidiasteridae | *Ophidiaster ophidianus* | 124101 | 279 |
| 196 | Echinodermata | Crinoidea | Comatulida | Antedonidae | *Antedon mediterranea* | 124208 | 679 |
| 197 | Echinodermata | Crinoidea | Comatulida | Antedonidae | *Leptometra phalangium* | 124226 | 268 |
| 198 | Echinodermata | Echinoidea | Arbacioida | Arbaciidae | *Arbacia lixula* | 124249 | 787 |
| 199 | Echinodermata | Echinoidea | Camarodonta | Echinidae | *Echinus melo* | 124294 | 627 |
| 200 | Echinodermata | Echinoidea | Camarodonta | Echinidae | *Gracilechinus acutus* | 532031 | 581 |
| 201 | Echinodermata | Echinoidea | Camarodonta | Parechinidae | *Paracentrotus lividus* | 124316 | 826 |
| 202 | Echinodermata | Echinoidea | Camarodonta | Parechinidae | *Psammechinus microtuberculatus* | 124318 | 204 |
| 203 | Echinodermata | Echinoidea | Camarodonta | Toxopneustidae | *Sphaerechinus granularis* | 124427 | 711 |
| 204 | Echinodermata | Echinoidea | Cidaroida | Cidaridae | *Cidaris cidaris* | 124257 | 636 |
| 205 | Echinodermata | Echinoidea | Cidaroida | Cidaridae | *Stylocidaris affinis* | 124268 | 431 |
| 206 | Echinodermata | Echinoidea | Diadematoida | Diadematidae | *Centrostephanus longispinus* | 124331 | 328 |
| 207 | Echinodermata | Echinoidea | Diadematoida | Diadematidae | *Diadema setosum* | 213372 | 161 |
| 208 | Echinodermata | Echinoidea | Spatangoida | Brissidae | *Brissopsis lyrifera* | 124373 | 125 |
| 209 | Echinodermata | Echinoidea | Spatangoida | Brissidae | *Brissus unicolor* | 124380 | 166 |
| 210 | Echinodermata | Echinoidea | Spatangoida | Loveniidae | *Echinocardium cordatum* | 124392 | 193 |
| 211 | Echinodermata | Echinoidea | Spatangoida | Schizasteridae | *Ova canalifera* | 569394 | 128 |
| 212 | Echinodermata | Echinoidea | Spatangoida | Spatangidae | *Spatangus purpureus* | 124418 | 637 |
| 213 | Echinodermata | Holothuroidea | Dendrochirotida | Cucumariidae | *Paraleptopentacta elongata* | 1474372 | 185 |
| 214 | Echinodermata | Holothuroidea | Dendrochirotida | Cucumariidae | *Paraleptopentacta tergestina* | 1474379 | 167 |
| 215 | Echinodermata | Holothuroidea | Dendrochirotida | Cucumariidae | *Ocnus planci* | 124647 | 358 |
| 216 | Echinodermata | Holothuroidea | Holothuriida | Holothuriidae | *Holothuria (Roweothuria) poli* | 124525 | 286 |
| 217 | Echinodermata | Holothuroidea | Holothuriida | Holothuriidae | *Holothuria (Platyperona) sanctori* | 124528 | 272 |
| 218 | Echinodermata | Holothuroidea | Holothuriida | Holothuriidae | *Holothuria (Holothuria) tubulosa* | 125182 | 645 |
| 219 | Echinodermata | Holothuroidea | Holothuriida | Holothuriidae | *Holothuria forskali* | 1670329 | 427 |
| 220 | Echinodermata | Holothuroidea | Synallactida | Stichopodidae | *Parastichopus regalis* | 149898 | 1554 |
| 221 | Echinodermata | Ophiuroidea | Amphilepidida | Amphiuridae | *Amphiura chiajei* | 125073 | 209 |
| 222 | Echinodermata | Ophiuroidea | Amphilepidida | Amphiuridae | *Amphiura filiformis* | 125080 | 126 |
| 223 | Echinodermata | Ophiuroidea | Amphilepidida | Ophiopsilidae | *Ophiopsila aranea* | 125049 | 177 |
| 224 | Echinodermata | Ophiuroidea | Amphilepidida | Ophiotrichidae | *Ophiothrix fragilis* | 125131 | 472 |
| 225 | Echinodermata | Ophiuroidea | Euryalida | Gorgonocephalidae | *Astrospartus mediterraneus* | 124963 | 144 |
| 226 | Echinodermata | Ophiuroidea | Ophiacanthida | Ophiodermatidae | *Ophioderma longicaudum* | 1420120 | 472 |
| 227 | Echinodermata | Ophiuroidea | Ophiurida | Ophiuridae | *Ophiura ophiura* | 124929 | 641 |
| 228 | Mollusca | Bivalvia | Adapedonta | Pharidae | *Ensis minor* | 140734 | 170 |
| 229 | Mollusca | Bivalvia | Adapedonta | Pharidae | *Phaxas pellucidus* | 140737 | 142 |
| 230 | Mollusca | Bivalvia | Arcida | Arcidae | *Arca noae* | 138788 | 627 |
| 231 | Mollusca | Bivalvia | Arcida | Arcidae | *Barbatia barbata* | 138793 | 238 |
| 232 | Mollusca | Bivalvia | Arcida | Glycymerididae | *Glycymeris nummaria* | 504509 | 164 |
| 233 | Mollusca | Bivalvia | Cardiida | Cardiidae | *Acanthocardia aculeata* | 138990 | 226 |
| 234 | Mollusca | Bivalvia | Cardiida | Cardiidae | *Acanthocardia echinata* | 138992 | 352 |
| 235 | Mollusca | Bivalvia | Cardiida | Cardiidae | *Acanthocardia tuberculata* | 381057 | 662 |
| 236 | Mollusca | Bivalvia | Cardiida | Cardiidae | *Cerastoderma glaucum* | 138999 | 176 |
| 237 | Mollusca | Bivalvia | Cardiida | Donacidae | *Donax semistriatus* | 139601 | 143 |
| 238 | Mollusca | Bivalvia | Cardiida | Donacidae | *Donax trunculus* | 139602 | 328 |
| 239 | Mollusca | Bivalvia | Cardiida | Donacidae | *Donax venustus* | 139603 | 129 |
| 240 | Mollusca | Bivalvia | Cardiida | Tellinidae | *Peronaea planata* | 605934 | 149 |
| 241 | Mollusca | Bivalvia | Limida | Limidae | *Lima lima* | 140233 | 176 |
| 242 | Mollusca | Bivalvia | Limida | Limidae | *Limaria hians* | 140235 | 146 |
| 243 | Mollusca | Bivalvia | Limida | Limidae | *Limaria tuberculata* | 140236 | 138 |
| 244 | Mollusca | Bivalvia | Lucinida | Lucinidae | *Loripes orbiculatus* | 875379 | 148 |
| 245 | Mollusca | Bivalvia | Mytilida | Mytilidae | *Lithophaga lithophaga* | 140459 | 187 |
| 246 | Mollusca | Bivalvia | Mytilida | Mytilidae | *Mytilus galloprovincialis* | 140481 | 419 |
| 247 | Mollusca | Bivalvia | Nuculida | Nuculidae | *Nucula sulcata* | 140592 | 121 |
| 248 | Mollusca | Bivalvia | Ostreida | Gryphaeidae | *Neopycnodonte cochlear* | 140048 | 417 |
| 249 | Mollusca | Bivalvia | Ostreida | Margaritidae | *Pinctada radiata* | 140890 | 132 |
| 250 | Mollusca | Bivalvia | Ostreida | Ostreidae | *Magallana gigas* | 836033 | 131 |
| 251 | Mollusca | Bivalvia | Ostreida | Ostreidae | *Ostrea edulis* | 140658 | 292 |
| 252 | Mollusca | Bivalvia | Ostreida | Pinnidae | *Pinna nobilis* | 140780 | 714 |
| 253 | Mollusca | Bivalvia | Ostreida | Pinnidae | *Pinna rudis* | 140781 | 228 |
| 254 | Mollusca | Bivalvia | Ostreida | Pteriidae | *Pteria hirundo* | 140891 | 425 |
| 255 | Mollusca | Bivalvia | Pectinida | Anomiidae | *Anomia ephippium* | 138748 | 333 |
| 256 | Mollusca | Bivalvia | Pectinida | Pectinidae | *Aequipecten opercularis* | 140687 | 246 |
| 257 | Mollusca | Bivalvia | Pectinida | Pectinidae | *Mimachlamys varia* | 236719 | 455 |
| 258 | Mollusca | Bivalvia | Pectinida | Pectinidae | *Pecten jacobaeus* | 394429 | 353 |
| 259 | Mollusca | Bivalvia | Pectinida | Spondylidae | *Spondylus gaederopus* | 141549 | 268 |
| 260 | Mollusca | Bivalvia | Venerida | Mactridae | *Mactra stultorum* | 140299 | 384 |
| 261 | Mollusca | Bivalvia | Venerida | Mactridae | *Spisula subtruncata* | 140302 | 273 |
| 262 | Mollusca | Bivalvia | Venerida | Mesodesmatidae | *Donacilla cornea* | 140350 | 126 |
| 263 | Mollusca | Bivalvia | Venerida | Veneridae | *Callista chione* | 141906 | 264 |
| 264 | Mollusca | Bivalvia | Venerida | Veneridae | *Chamelea gallina* | 141907 | 475 |
| 265 | Mollusca | Bivalvia | Venerida | Veneridae | *Dosinia lupinus* | 141912 | 191 |
| 266 | Mollusca | Bivalvia | Venerida | Veneridae | *Polititapes aureus* | 246150 | 139 |
| 267 | Mollusca | Bivalvia | Venerida | Veneridae | *Ruditapes decussatus* | 231749 | 148 |
| 268 | Mollusca | Bivalvia | Venerida | Veneridae | *Venus nux* | 141935 | 141 |
| 269 | Mollusca | Bivalvia | Venerida | Veneridae | *Venus verrucosa* | 141936 | 378 |
| 270 | Mollusca | Gastropoda |  | Patellidae | *Patella caerulea* | 140677 | 356 |
| 271 | Mollusca | Gastropoda |  | Patellidae | *Patella rustica* | 140683 | 218 |
| 272 | Mollusca | Gastropoda |  | Patellidae | *Patella ulyssiponensis* | 140684 | 154 |
| 273 | Mollusca | Gastropoda |  | Plakobranchidae | *Elysia timida* | 139684 | 150 |
| 274 | Mollusca | Gastropoda |  | Plakobranchidae | *Thuridilla hopei* | 139687 | 217 |
| 275 | Mollusca | Gastropoda | Aplysiida | Aplysiidae | *Aplysia fasciata* | 138755 | 164 |
| 276 | Mollusca | Gastropoda | Aplysiida | Aplysiidae | *Aplysia punctata* | 138758 | 144 |
| 277 | Mollusca | Gastropoda | Caenogastropoda incertae sedis | Cerithiidae | *Bittium reticulatum* | 139054 | 151 |
| 278 | Mollusca | Gastropoda | Caenogastropoda incertae sedis | Cerithiidae | *Cerithium vulgatum* | 139066 | 530 |
| 279 | Mollusca | Gastropoda | Caenogastropoda incertae sedis | Turritellidae | *Turritellinella tricarinata* | 1381415 | 361 |
| 280 | Mollusca | Gastropoda | Cephalaspidea | Scaphandridae | *Scaphander lignarius* | 139488 | 373 |
| 281 | Mollusca | Gastropoda | Lepetellida | Haliotidae | *Haliotis tuberculata* | 140059 | 390 |
| 282 | Mollusca | Gastropoda | Littorinimorpha | Aporrhaidae | *Aporrhais pespelecani* | 138760 | 535 |
| 283 | Mollusca | Gastropoda | Littorinimorpha | Aporrhaidae | *Aporrhais serresiana* | 138761 | 131 |
| 284 | Mollusca | Gastropoda | Littorinimorpha | Calyptraeidae | *Calyptraea chinensis* | 138961 | 217 |
| 285 | Mollusca | Gastropoda | Littorinimorpha | Cassidae | *Galeodea echinophora* | 139023 | 1180 |
| 286 | Mollusca | Gastropoda | Littorinimorpha | Cassidae | *Galeodea rugosa* | 139024 | 460 |
| 287 | Mollusca | Gastropoda | Littorinimorpha | Cypraeidae | *Luria lurida* | 139499 | 186 |
| 288 | Mollusca | Gastropoda | Littorinimorpha | Littorinidae | *Echinolittorina punctata* | 345757 | 123 |
| 289 | Mollusca | Gastropoda | Littorinimorpha | Littorinidae | *Melarhaphe neritoides* | 140266 | 190 |
| 290 | Mollusca | Gastropoda | Littorinimorpha | Naticidae | *Euspira fusca* | 140529 | 167 |
| 291 | Mollusca | Gastropoda | Littorinimorpha | Naticidae | *Naticarius stercusmuscarum* | 720574 | 197 |
| 292 | Mollusca | Gastropoda | Littorinimorpha | Naticidae | *Neverita josephinia* | 140549 | 210 |
| 293 | Mollusca | Gastropoda | Littorinimorpha | Ranellidae | *Ranella olearium* | 141115 | 139 |
| 294 | Mollusca | Gastropoda | Littorinimorpha | Strombidae | *Conomurex persicus* | 565371 | 129 |
| 295 | Mollusca | Gastropoda | Littorinimorpha | Tonnidae | *Tonna galea* | 141687 | 196 |
| 296 | Mollusca | Gastropoda | Littorinimorpha | Vermetidae | *Thylacodes arenarius* | 709464 | 150 |
| 297 | Mollusca | Gastropoda | Littorinimorpha | Xenophoridae | *Xenophora crispa* | 743862 | 187 |
| 298 | Mollusca | Gastropoda | Neogastropoda | Columbellidae | *Columbella rustica* | 139196 | 385 |
| 299 | Mollusca | Gastropoda | Neogastropoda | Conidae | *Conus ventricosus* | 428401 | 317 |
| 300 | Mollusca | Gastropoda | Neogastropoda | Muricidae | *Bolinus brandaris* | 140389 | 950 |
| 301 | Mollusca | Gastropoda | Neogastropoda | Muricidae | *Hexaplex trunculus* | 140396 | 698 |
| 302 | Mollusca | Gastropoda | Neogastropoda | Muricidae | *Ocenebra erinaceus* | 140405 | 129 |
| 303 | Mollusca | Gastropoda | Neogastropoda | Muricidae | *Stramonita haemastoma* | 140417 | 416 |
| 304 | Mollusca | Gastropoda | Neogastropoda | Nassariidae | *Tritia incrassata* | 876825 | 168 |
| 305 | Mollusca | Gastropoda | Neogastropoda | Nassariidae | *Tritia mutabilis* | 876840 | 234 |
| 306 | Mollusca | Gastropoda | Neogastropoda | Pisaniidae | *Pisania striata* | 138924 | 122 |
| 307 | Mollusca | Gastropoda | Neogastropoda | Tudiclidae | *Euthria cornea* | 181057 | 151 |
| 308 | Mollusca | Gastropoda | Nudibranchia | Chromodorididae | *Felimare picta* | 597522 | 307 |
| 309 | Mollusca | Gastropoda | Nudibranchia | Chromodorididae | *Felimare tricolor* | 597530 | 194 |
| 310 | Mollusca | Gastropoda | Nudibranchia | Chromodorididae | *Felimare orsinii* | 597533 | 138 |
| 311 | Mollusca | Gastropoda | Nudibranchia | Chromodorididae | *Felimare villafranca* | 597536 | 123 |
| 312 | Mollusca | Gastropoda | Nudibranchia | Discodorididae | *Peltodoris atromaculata* | 509315 | 369 |
| 313 | Mollusca | Gastropoda | Nudibranchia | Facelinidae | *Cratena peregrina* | 146862 | 355 |
| 314 | Mollusca | Gastropoda | Nudibranchia | Flabellinidae | *Edmundsella pedata* | 1047602 | 192 |
| 315 | Mollusca | Gastropoda | Nudibranchia | Flabellinidae | *Flabellina affinis* | 139988 | 372 |
| 316 | Mollusca | Gastropoda | Nudibranchia | Flabellinidae | *Paraflabellina ischitana* | 1048137 | 145 |
| 317 | Mollusca | Gastropoda | Nudibranchia | Tethydidae | *Tethys fimbria* | 141643 | 286 |
| 318 | Mollusca | Gastropoda | Pleurobranchida | Pleurobranchaeidae | *Pleurobranchaea meckeli* | 140818 | 402 |
| 319 | Mollusca | Gastropoda | Trochida | Calliostomatidae | *Calliostoma granulatum* | 141753 | 513 |
| 320 | Mollusca | Gastropoda | Trochida | Trochidae | *Phorcus articulatus* | 689174 | 172 |
| 321 | Mollusca | Gastropoda | Trochida | Trochidae | *Phorcus turbinatus* | 689179 | 723 |
| 322 | Mollusca | Gastropoda | Trochida | Turbinidae | *Bolma rugosa* | 141855 | 312 |
| 323 | Mollusca | Gastropoda | Umbraculida | Umbraculidae | *Umbraculum umbraculum* | 141879 | 223 |
| 324 | Mollusca | Polyplacophora | Chitonida | Chitonidae | *Rhyssoplax olivacea* | 1392276 | 196 |
| 325 | Porifera | Calcarea | Clathrinida | Clathrinidae | *Clathrina clathrus* | 132275 | 194 |
| 326 | Porifera | Demospongiae | Agelasida | Agelasidae | *Agelas oroides* | 132454 | 303 |
| 327 | Porifera | Demospongiae | Axinellida | Axinellidae | *Axinella damicornis* | 132472 | 358 |
| 328 | Porifera | Demospongiae | Axinellida | Axinellidae | *Axinella polypoides* | 132487 | 270 |
| 329 | Porifera | Demospongiae | Axinellida | Axinellidae | *Axinella verrucosa* | 132499 | 236 |
| 330 | Porifera | Demospongiae | Bubarida | Dictyonellidae | *Acanthella acuta* | 132455 | 168 |
| 331 | Porifera | Demospongiae | Chondrillida | Chondrillidae | *Chondrilla nucula* | 134110 | 161 |
| 332 | Porifera | Demospongiae | Chondrosiida | Chondrosiidae | *Chondrosia reniformis* | 134112 | 435 |
| 333 | Porifera | Demospongiae | Clionaida | Clionaidae | *Cliona celata* | 134121 | 211 |
| 334 | Porifera | Demospongiae | Clionaida | Clionaidae | *Cliona viridis* | 134146 | 234 |
| 335 | Porifera | Demospongiae | Clionaida | Spirastrellidae | *Spirastrella cunctatrix* | 134235 | 157 |
| 336 | Porifera | Demospongiae | Dictyoceratida | Dysideidae | *Dysidea fragilis* | 132324 | 159 |
| 337 | Porifera | Demospongiae | Dictyoceratida | Dysideidae | *Pleraplysilla spinifera* | 132317 | 138 |
| 338 | Porifera | Demospongiae | Dictyoceratida | Irciniidae | *Ircinia oros* | 132356 | 169 |
| 339 | Porifera | Demospongiae | Dictyoceratida | Irciniidae | *Ircinia variabilis* | 132362 | 238 |
| 340 | Porifera | Demospongiae | Dictyoceratida | Irciniidae | *Sarcotragus fasciculatus* | 165081 | 181 |
| 341 | Porifera | Demospongiae | Dictyoceratida | Irciniidae | *Sarcotragus spinosulus* | 165086 | 370 |
| 342 | Porifera | Demospongiae | Dictyoceratida | Spongiidae | *Spongia (Spongia) officinalis* | 165220 | 278 |
| 343 | Porifera | Demospongiae | Dictyoceratida | Thorectidae | *Scalarispongia scalaris* | 165376 | 156 |
| 344 | Porifera | Demospongiae | Haplosclerida | Petrosiidae | *Petrosia (Petrosia) ficiformis* | 166837 | 378 |
| 345 | Porifera | Demospongiae | Poecilosclerida | Crambeidae | *Crambe crambe* | 133445 | 471 |
| 346 | Porifera | Demospongiae | Poecilosclerida | Hymedesmiidae | *Hemimycale columella* | 133543 | 277 |
| 347 | Porifera | Demospongiae | Poecilosclerida | Hymedesmiidae | *Phorbas tenacior* | 133693 | 280 |
| 348 | Porifera | Demospongiae | Suberitida | Suberitidae | *Suberites domuncula* | 134282 | 550 |
| 349 | Porifera | Demospongiae | Tethyida | Tethyidae | *Tethya aurantium* | 134311 | 210 |
| 350 | Porifera | Demospongiae | Tetractinellida | Theneidae | *Thenea muricata* | 134106 | 145 |
| 351 | Porifera | Demospongiae | Verongiida | Aplysinidae | *Aplysina aerophoba* | 133911 | 444 |
| 352 | Porifera | Demospongiae | Verongiida | Aplysinidae | *Aplysina cavernicola* | 133913 | 153 |
| 353 | Porifera | Homoscleromorpha | Homosclerophorida | Oscarellidae | *Oscarella lobularis* | 133928 | 171 |

**Table S2** – Performances of the fitted models. The field AphiaID refers to the Aphia code in the Darwin Core Standard. AUC = Area under the ROC curve; TSS = True Skill Statistics, BOYCE = Boyce’s index.

| **Name** | **AphiaID** | **Training AUC** | **Evaluation AUC** | **Training TSS** | **Evaluation TSS** | **Training BOYCE** | **Evaluation BOYCE** |
| --- | --- | --- | --- | --- | --- | --- | --- |
| *Heteromastus filiformis* | 129884 | 0.9 | 0.91 | 0.71 | 0.73 | 0.76 | 0.85 |
| *Notomastus latericeus* | 129898 | 0.94 | 0.74 | 0.8 | 0.41 | 0.98 | 0.47 |
| *Myriochele heeri* | 130542 | 0.93 | 0.98 | 0.73 | 0.9 | 0.96 | 0.89 |
| *Owenia fusiformis* | 130544 | 0.92 | 0.92 | 0.71 | 0.69 | 0.99 | 0.38 |
| *Hermodice carunculata* | 129831 | 0.9 | 0.92 | 0.67 | 0.74 | 0.95 | 0.96 |
| *Bonellia viridis* | 110363 | 0.87 | 0.74 | 0.6 | 0.52 | 0.99 | 0.33 |
| *Eunice vittata* | 130067 | 0.94 | 0.88 | 0.76 | 0.68 | 0.95 | 0.4 |
| *Lumbrineris latreilli* | 130248 | 0.93 | 0.92 | 0.73 | 0.73 | 0.96 | 0.71 |
| *Drilonereis filum* | 129856 | 0.9 | 0.9 | 0.7 | 0.74 | 0.92 | 0.87 |
| *Aponuphis bilineata* | 130452 | 0.87 | 0.92 | 0.57 | 0.76 | 0.99 | 0.86 |
| *Hyalinoecia tubicola* | 130464 | 0.81 | 0.89 | 0.48 | 0.63 | 0.98 | 0.88 |
| *Aphrodita aculeata* | 129840 | 0.81 | 0.8 | 0.49 | 0.57 | 0.99 | 0.78 |
| *Laetmonice hystrix* | 129845 | 0.87 | 0.83 | 0.61 | 0.57 | 0.98 | 0.73 |
| *Glycera alba* | 130116 | 0.9 | 0.98 | 0.67 | 0.88 | 0.88 | 0.92 |
| *Glycera tridactyla* | 130130 | 0.96 | 0.92 | 0.83 | 0.8 | 0.94 | 0.24 |
| *Glycera unicornis* | 130131 | 0.93 | 0.93 | 0.76 | 0.7 | 0.97 | 0.76 |
| *Goniada maculata* | 130140 | 0.91 | 0.81 | 0.7 | 0.6 | 0.99 | 0.85 |
| *Nephtys hombergii* | 130359 | 0.95 | 0.98 | 0.81 | 0.89 | 0.97 | 0.88 |
| *Sigalion mathildae* | 131072 | 0.97 | 0.97 | 0.87 | 0.86 | 0.95 | 0.81 |
| *Sabella pavonina* | 130967 | 0.88 | 0.9 | 0.66 | 0.62 | 0.95 | 0.89 |
| *Sabella spallanzanii* | 130969 | 0.85 | 0.84 | 0.59 | 0.56 | 0.98 | 0.9 |
| *Protula intestinum* | 131032 | 0.85 | 0.84 | 0.59 | 0.6 | 0.93 | 0.56 |
| *Protula tubularia* | 131035 | 0.89 | 0.78 | 0.64 | 0.47 | 0.99 | 0.84 |
| *Serpula vermicularis* | 131051 | 0.86 | 0.85 | 0.57 | 0.56 | 0.96 | 0.73 |
| *Laonice cirrata* | 131128 | 0.88 | 0.87 | 0.67 | 0.65 | 0.97 | 0.66 |
| *Prionospio steenstrupi* | 131164 | 0.93 | 0.96 | 0.75 | 0.8 | 0.97 | 0.93 |
| *Ampharete grubei* | 152272 | 0.93 | 0.95 | 0.76 | 0.83 | 0.81 | 0.84 |
| *Melinna palmata* | 129808 | 0.92 | 0.92 | 0.72 | 0.72 | 0.97 | 0.76 |
| *Sternaspis scutata* | 131242 | 0.89 | 0.89 | 0.67 | 0.79 | 0.94 | 0.46 |
| *Ampelisca sarsi* | 101923 | 0.95 | 1 | 0.8 | 0.93 | 0.93 | 0.77 |
| *Ampelisca typica* | 101933 | 0.96 | 0.93 | 0.82 | 0.71 | 0.95 | 0.72 |
| *Athanas nitescens* | 107486 | 0.93 | 0.96 | 0.76 | 0.76 | 0.96 | 0.91 |
| *Calocaris macandreae* | 107726 | 0.91 | 0.83 | 0.68 | 0.7 | 0.86 | 0.68 |
| *Calappa granulata* | 107268 | 0.84 | 0.83 | 0.53 | 0.58 | 0.96 | 0.9 |
| *Procambarus clarkii* | 465540 | 0.98 | 0.91 | 0.85 | 0.83 | 0.93 | 0.66 |
| *Carcinus aestuarii* | 107380 | 0.92 | 0.93 | 0.72 | 0.76 | 0.97 | 0.92 |
| *Aegaeon cataphractus* | 107548 | 0.88 | 0.9 | 0.64 | 0.66 | 0.99 | 0.75 |
| *Pontophilus spinosus* | 107564 | 0.84 | 0.79 | 0.52 | 0.54 | 1 | 0.52 |
| *Clibanarius erythropus* | 107196 | 0.87 | 0.89 | 0.6 | 0.66 | 0.99 | 0.89 |
| *Dardanus arrosor* | 107197 | 0.85 | 0.81 | 0.55 | 0.49 | 1 | 0.89 |
| *Dardanus calidus* | 107198 | 0.89 | 0.83 | 0.66 | 0.54 | 0.99 | 0.88 |
| *Diogenes pugilator* | 107199 | 0.93 | 0.88 | 0.71 | 0.64 | 0.97 | 0.85 |
| *Paguristes eremita* | 107200 | 0.92 | 0.79 | 0.69 | 0.59 | 0.95 | 0.57 |
| *Medorippe lanata* | 107288 | 0.85 | 0.85 | 0.57 | 0.59 | 0.99 | 0.91 |
| *Dromia personata* | 107258 | 0.85 | 0.85 | 0.57 | 0.62 | 0.95 | 0.87 |
| *Pisa armata* | 107353 | 0.92 | 0.83 | 0.69 | 0.6 | 0.98 | 0.56 |
| *Eriphia verrucosa* | 107409 | 0.89 | 0.89 | 0.63 | 0.62 | 0.99 | 0.91 |
| *Ethusa mascarone* | 107283 | 0.93 | 0.85 | 0.72 | 0.64 | 0.89 | 0.8 |
| *Galathea intermedia* | 107150 | 0.92 | 0.91 | 0.72 | 0.8 | 0.96 | 0.89 |
| *Galathea strigosa* | 107155 | 0.91 | 0.87 | 0.7 | 0.64 | 0.94 | 0.86 |
| *Geryon longipes* | 107373 | 0.9 | 0.73 | 0.68 | 0.35 | 0.99 | 0.85 |
| *Goneplax rhomboides* | 107292 | 0.81 | 0.77 | 0.5 | 0.45 | 0.99 | 0.89 |
| *Pachygrapsus marmoratus* | 107455 | 0.89 | 0.88 | 0.65 | 0.62 | 1 | 0.92 |
| *Homola barbata* | 107262 | 0.89 | 0.75 | 0.65 | 0.43 | 0.94 | 0.55 |
| *Paromola cuvieri* | 107264 | 0.9 | 0.85 | 0.73 | 0.78 | 0.97 | 0.65 |
| *Inachus communissimus* | 107326 | 0.92 | 0.91 | 0.78 | 0.76 | 0.95 | 0.48 |
| *Inachus dorsettensis* | 107327 | 0.9 | 0.89 | 0.7 | 0.68 | 0.96 | 0.88 |
| *Inachus phalangium* | 107333 | 0.91 | 0.8 | 0.72 | 0.49 | 0.88 | 0.49 |
| *Inachus thoracicus* | 107334 | 0.93 | 0.74 | 0.73 | 0.52 | 0.98 | 0.75 |
| *Macropodia linaresi* | 107341 | 0.94 | 0.98 | 0.76 | 0.93 | 0.91 | 0.48 |
| *Macropodia longirostris* | 107343 | 0.91 | 0.78 | 0.69 | 0.58 | 0.94 | -0.24 |
| *Macropodia rostrata* | 107345 | 0.93 | 0.81 | 0.73 | 0.54 | 0.97 | 0.87 |
| *Macropodia tenuirostris* | 107346 | 0.86 | 0.86 | 0.58 | 0.57 | 1 | 0.91 |
| *Latreillia elegans* | 107265 | 0.9 | 0.8 | 0.67 | 0.5 | 0.93 | 0.87 |
| *Lysmata seticaudata* | 107528 | 0.88 | 0.88 | 0.65 | 0.72 | 0.97 | 0.88 |
| *Maja crispata* | 107348 | 0.89 | 0.85 | 0.62 | 0.57 | 1 | 0.85 |
| *Maja squinado* | 107350 | 0.84 | 0.79 | 0.54 | 0.53 | 0.99 | 0.9 |
| *Iridonida speciosa* | 1606714 | 0.92 | 0.79 | 0.68 | 0.52 | 1 | 0.77 |
| *Munida intermedia* | 107157 | 0.87 | 0.85 | 0.62 | 0.67 | 0.94 | 0.93 |
| *Munida tenuimana* | 107166 | 0.94 | 0.87 | 0.79 | 0.64 | 0.97 | 0.87 |
| *Homarus gammarus* | 107253 | 0.86 | 0.91 | 0.59 | 0.74 | 0.91 | 0.89 |
| *Pagurus alatus* | 107230 | 0.86 | 0.79 | 0.55 | 0.56 | 0.99 | 0.12 |
| *Pagurus anachoretus* | 107231 | 0.87 | 0.87 | 0.6 | 0.67 | 0.96 | 0.48 |
| *Pagurus cuanensis* | 107235 | 0.92 | 0.89 | 0.73 | 0.75 | 0.98 | 0.92 |
| *Pagurus excavatus* | 107236 | 0.83 | 0.88 | 0.54 | 0.59 | 0.99 | 0.98 |
| *Pagurus prideaux* | 107239 | 0.9 | 0.79 | 0.68 | 0.44 | 0.99 | 0.75 |
| *Palaemon elegans* | 107614 | 0.89 | 0.86 | 0.63 | 0.66 | 0.97 | 0.88 |
| *Palaemon serratus* | 107616 | 0.92 | 0.91 | 0.71 | 0.7 | 0.97 | 0.85 |
| *Palinurus elephas* | 107703 | 0.86 | 0.84 | 0.61 | 0.56 | 1 | 0.93 |
| *Chlorotocus crassicornis* | 107642 | 0.82 | 0.8 | 0.53 | 0.55 | 0.99 | 0.37 |
| *Plesionika acanthonotus* | 107654 | 0.87 | 0.88 | 0.61 | 0.62 | 0.97 | 0.61 |
| *Plesionika antigai* | 107655 | 0.89 | 0.84 | 0.66 | 0.57 | 0.97 | 0.34 |
| *Plesionika edwardsii* | 107656 | 0.89 | 0.8 | 0.67 | 0.54 | 0.98 | 0.73 |
| *Plesionika gigliolii* | 107659 | 0.9 | 0.84 | 0.67 | 0.56 | 0.98 | 0.85 |
| *Plesionika heterocarpus* | 107660 | 0.84 | 0.84 | 0.56 | 0.63 | 1 | 0.86 |
| *Plesionika martia* | 107661 | 0.87 | 0.82 | 0.63 | 0.6 | 0.99 | 0.85 |
| *Plesionika narval* | 107662 | 0.91 | 0.95 | 0.68 | 0.78 | 0.96 | 0.89 |
| *Spinolambrus macrochelos* | 442337 | 0.84 | 0.8 | 0.53 | 0.59 | 0.98 | 0.52 |
| *Penaeus kerathurus* | 246388 | 0.92 | 0.95 | 0.72 | 0.82 | 0.92 | 0.97 |
| *Pilumnus hirtellus* | 107418 | 0.92 | 0.8 | 0.72 | 0.54 | 0.92 | 0.4 |
| *Pilumnus spinifer* | 107420 | 0.92 | 0.86 | 0.69 | 0.56 | 0.99 | 0.85 |
| *Pilumnus villosissimus* | 107421 | 0.93 | 0.96 | 0.69 | 0.84 | 0.95 | 0.95 |
| *Bathynectes maravigna* | 107377 | 0.91 | 0.83 | 0.67 | 0.61 | 0.99 | 0.69 |
| *Polybius depurator* | 1750291 | 0.78 | 0.85 | 0.45 | 0.64 | 1 | 0.88 |
| *Polybius vernalis* | 1750288 | 0.93 | 0.99 | 0.73 | 0.9 | 0.96 | 0.92 |
| *Macropipus tuberculatus* | 107397 | 0.82 | 0.88 | 0.48 | 0.63 | 0.97 | 0.99 |
| *Polycheles typhlops* | 107696 | 0.87 | 0.83 | 0.64 | 0.61 | 0.99 | 0.95 |
| *Processa canaliculata* | 107682 | 0.84 | 0.76 | 0.6 | 0.51 | 0.98 | 0.78 |
| *Processa nouveli* | 107689 | 0.96 | 0.97 | 0.83 | 0.91 | 0.91 | 0.89 |
| *Scyllarides latus* | 107708 | 0.88 | 0.81 | 0.67 | 0.62 | 0.98 | 0.69 |
| *Solenocera membranacea* | 107120 | 0.83 | 0.78 | 0.52 | 0.52 | 0.99 | 0.94 |
| *Stenopus spinosus* | 107721 | 0.9 | 0.73 | 0.65 | 0.52 | 0.86 | -0.27 |
| *Monodaeus couchii* | 241154 | 0.91 | 0.9 | 0.69 | 0.74 | 0.99 | 0.89 |
| *Xantho poressa* | 107442 | 0.89 | 0.88 | 0.64 | 0.62 | 0.98 | 0.74 |
| *Natatolana borealis* | 118859 | 0.93 | 0.92 | 0.72 | 0.78 | 0.88 | 0.81 |
| *Rissoides pallidus* | 136136 | 0.91 | 0.89 | 0.66 | 0.72 | 0.97 | 0.94 |
| *Squilla mantis* | 136137 | 0.89 | 0.82 | 0.65 | 0.62 | 0.98 | 0.55 |
| *Apseudopsis latreillii* | 247077 | 0.97 | 0.71 | 0.87 | 0.56 | 0.88 | 0.73 |
| *Adeonella calveti* | 111055 | 0.85 | 0.85 | 0.58 | 0.64 | 0.97 | 0.88 |
| *Reptadeonella violacea* | 111061 | 0.93 | 0.75 | 0.74 | 0.5 | 0.97 | 0.41 |
| *Pentapora fascialis* | 111082 | 0.85 | 0.88 | 0.62 | 0.69 | 0.96 | 0.77 |
| *Schizomavella (Schizomavella) mamillata* | 862797 | 0.91 | 0.84 | 0.72 | 0.66 | 0.95 | 0.66 |
| *Electra posidoniae* | 111356 | 0.88 | 0.91 | 0.65 | 0.68 | 0.98 | 0.78 |
| *Myriapora truncata* | 111435 | 0.83 | 0.9 | 0.52 | 0.73 | 0.99 | 0.91 |
| *Reteporella grimaldii* | 111453 | 0.87 | 0.91 | 0.63 | 0.74 | 0.95 | 0.86 |
| *Schizobrachiella sanguinea* | 111522 | 0.87 | 0.81 | 0.59 | 0.6 | 0.77 | 0.19 |
| *Smittina cervicornis* | 111551 | 0.88 | 0.8 | 0.66 | 0.49 | 0.99 | 0.79 |
| *Clavelina lepadiformis* | 103552 | 0.88 | 0.95 | 0.61 | 0.83 | 0.98 | 0.98 |
| *Diazona violacea* | 103733 | 0.84 | 0.83 | 0.53 | 0.55 | 0.96 | 0.72 |
| *Aplidium conicum* | 103641 | 0.89 | 0.85 | 0.61 | 0.58 | 0.94 | 0.7 |
| *Aplidium elegans* | 103643 | 0.93 | 0.88 | 0.74 | 0.66 | 0.96 | 0.41 |
| *Ascidia mentula* | 103710 | 0.84 | 0.88 | 0.56 | 0.69 | 0.99 | 0.81 |
| *Ascidia virginea* | 103717 | 0.88 | 0.7 | 0.61 | 0.47 | 0.97 | 0.32 |
| *Ascidiella scabra* | 103719 | 0.89 | 0.84 | 0.7 | 0.61 | 0.86 | 0.47 |
| *Phallusia mammillata* | 103724 | 0.84 | 0.83 | 0.56 | 0.59 | 0.97 | 0.68 |
| *Ciona intestinalis* | 103732 | 0.86 | 0.78 | 0.54 | 0.53 | 0.94 | 0.46 |
| *Molgula appendiculata* | 103771 | 0.93 | 0.92 | 0.73 | 0.8 | 0.93 | 0.57 |
| *Halocynthia papillosa* | 103827 | 0.87 | 0.82 | 0.61 | 0.51 | 1 | 0.9 |
| *Microcosmus sabatieri* | 103844 | 0.85 | 0.91 | 0.62 | 0.73 | 0.96 | 0.96 |
| *Microcosmus vulgaris* | 103846 | 0.86 | 0.77 | 0.57 | 0.49 | 0.99 | 0.62 |
| *Botryllus schlosseri* | 103862 | 0.86 | 0.85 | 0.57 | 0.72 | 0.97 | 0.39 |
| *Actinia mediterranea* | 854459 | 0.88 | 0.79 | 0.63 | 0.47 | 0.98 | 0.9 |
| *Anemonia viridis* | 100808 | 0.86 | 0.81 | 0.56 | 0.49 | 0.97 | 0.96 |
| *Cribrinopsis crassa* | 100823 | 0.94 | 0.92 | 0.78 | 0.8 | 0.98 | 0.86 |
| *Aiptasia mutabilis* | 100859 | 0.84 | 0.88 | 0.52 | 0.67 | 0.99 | 0.97 |
| *Actinauge richardi* | 100930 | 0.88 | 0.81 | 0.61 | 0.55 | 0.98 | 0.81 |
| *Calliactis palliata* | 1658483 | 0.88 | 0.86 | 0.63 | 0.6 | 0.98 | 0.9 |
| *Calliactis parasitica* | 100946 | 0.8 | 0.84 | 0.47 | 0.51 | 0.99 | 0.91 |
| *Cereus pedunculatus* | 100987 | 0.92 | 0.76 | 0.78 | 0.38 | 0.94 | 0.59 |
| *Cerianthus membranacea* | 856035 | 0.92 | 0.9 | 0.7 | 0.71 | 0.98 | 0.78 |
| *Cerianthus membranaceus* | 101011 | 0.9 | 0.88 | 0.64 | 0.66 | 0.95 | 0.85 |
| *Caryophyllia (Caryophyllia) inornata* | 135141 | 0.95 | 0.81 | 0.82 | 0.52 | 0.97 | 0.79 |
| *Caryophyllia (Caryophyllia) smithii* | 135144 | 0.88 | 0.8 | 0.64 | 0.54 | 0.96 | 0.75 |
| *Cladocora caespitosa* | 135146 | 0.81 | 0.84 | 0.47 | 0.56 | 0.99 | 0.94 |
| *Astroides calycularis* | 135178 | 0.88 | 0.82 | 0.61 | 0.58 | 0.96 | 0.82 |
| *Balanophyllia (Balanophyllia) europaea* | 135180 | 0.85 | 0.83 | 0.55 | 0.6 | 0.98 | 0.75 |
| *Leptopsammia pruvoti* | 135193 | 0.88 | 0.76 | 0.61 | 0.41 | 0.98 | 0.69 |
| *Oculina patagonica* | 135210 | 0.93 | 0.95 | 0.75 | 0.86 | 0.95 | 0.77 |
| *Parazoanthus axinellae* | 101055 | 0.85 | 0.85 | 0.56 | 0.64 | 0.99 | 0.86 |
| *Paramuricea clavata* | 125387 | 0.83 | 0.79 | 0.56 | 0.48 | 0.98 | 0.88 |
| *Alcyonium acaule* | 125331 | 0.88 | 0.8 | 0.6 | 0.57 | 0.95 | 0.7 |
| *Alcyonium coralloides* | 125332 | 0.9 | 0.77 | 0.7 | 0.46 | 0.98 | 0.53 |
| *Alcyonium palmatum* | 125334 | 0.73 | 0.85 | 0.38 | 0.58 | 0.99 | 0.92 |
| *Eunicella cavolini* | 125361 | 0.88 | 0.88 | 0.63 | 0.61 | 0.98 | 0.98 |
| *Eunicella singularis* | 125365 | 0.87 | 0.88 | 0.63 | 0.67 | 0.96 | 0.93 |
| *Eunicella verrucosa* | 125366 | 0.9 | 0.79 | 0.68 | 0.52 | 0.97 | 0.66 |
| *Leptogorgia sarmentosa* | 125369 | 0.88 | 0.76 | 0.63 | 0.48 | 0.99 | 0.69 |
| *Corallium rubrum* | 125416 | 0.84 | 0.82 | 0.53 | 0.66 | 0.98 | 0.81 |
| *Funiculina quadrangularis* | 128506 | 0.8 | 0.82 | 0.48 | 0.55 | 0.99 | 0.79 |
| *Isidella elongata* | 125373 | 0.88 | 0.8 | 0.64 | 0.64 | 0.96 | 0.34 |
| *Pennatula phosphorea* | 128517 | 0.83 | 0.74 | 0.52 | 0.58 | 0.98 | -0.64 |
| *Pennatula rubra* | 128519 | 0.84 | 0.86 | 0.59 | 0.61 | 0.99 | 0.95 |
| *Pteroeides griseum* | 181504 | 0.84 | 0.9 | 0.57 | 0.67 | 0.98 | 0.84 |
| *Veretillum cynomorium* | 128536 | 0.9 | 0.97 | 0.66 | 0.88 | 0.99 | 0.93 |
| *Coscinasterias tenuispina* | 123795 | 0.86 | 0.83 | 0.59 | 0.55 | 0.89 | 0.85 |
| *Marthasterias glacialis* | 123803 | 0.82 | 0.84 | 0.52 | 0.54 | 0.99 | 0.77 |
| *Astropecten aranciacus* | 123856 | 0.82 | 0.78 | 0.49 | 0.41 | 1 | 0.96 |
| *Astropecten bispinosus* | 123859 | 0.87 | 0.97 | 0.56 | 0.85 | 0.97 | 0.82 |
| *Astropecten irregularis* | 123867 | 0.88 | 0.88 | 0.64 | 0.63 | 0.98 | 0.74 |
| *Astropecten jonstoni* | 123868 | 0.91 | 0.89 | 0.66 | 0.64 | 0.99 | 0.91 |
| *Astropecten platyacanthus* | 123876 | 0.85 | 0.84 | 0.58 | 0.57 | 0.95 | 0.79 |
| *Astropecten spinulosus* | 123878 | 0.86 | 0.88 | 0.56 | 0.61 | 0.97 | 0.62 |
| *Tethyaster subinermis* | 123913 | 0.85 | 0.82 | 0.58 | 0.48 | 1 | 0.81 |
| *Luidia ciliaris* | 123920 | 0.87 | 0.9 | 0.6 | 0.65 | 0.98 | 0.8 |
| *Luidia sarsii* | 123922 | 0.89 | 0.85 | 0.63 | 0.61 | 0.98 | 0.63 |
| *Echinaster (Echinaster) sepositus* | 125161 | 0.82 | 0.86 | 0.51 | 0.66 | 0.98 | 0.93 |
| *Anseropoda placenta* | 123985 | 0.82 | 0.88 | 0.53 | 0.67 | 0.99 | 0.84 |
| *Chaetaster longipes* | 124004 | 0.88 | 0.8 | 0.62 | 0.45 | 0.96 | 0.69 |
| *Peltaster placenta* | 124055 | 0.83 | 0.72 | 0.53 | 0.42 | 0.94 | 0.34 |
| *Hacelia attenuata* | 124094 | 0.88 | 0.77 | 0.66 | 0.44 | 0.99 | 0.74 |
| *Ophidiaster ophidianus* | 124101 | 0.86 | 0.86 | 0.58 | 0.62 | 0.98 | 0.84 |
| *Arbacia lixula* | 124249 | 0.87 | 0.81 | 0.57 | 0.48 | 0.94 | 0.94 |
| *Echinus melo* | 124294 | 0.78 | 0.83 | 0.43 | 0.56 | 1 | 0.84 |
| *Gracilechinus acutus* | 532031 | 0.82 | 0.73 | 0.51 | 0.43 | 0.95 | 0.52 |
| *Paracentrotus lividus* | 124316 | 0.86 | 0.84 | 0.59 | 0.56 | 0.99 | 0.94 |
| *Psammechinus microtuberculatus* | 124318 | 0.9 | 0.86 | 0.67 | 0.57 | 0.98 | 0.88 |
| *Sphaerechinus granularis* | 124427 | 0.88 | 0.81 | 0.6 | 0.53 | 0.98 | 0.85 |
| *Stylocidaris affinis* | 124268 | 0.89 | 0.76 | 0.65 | 0.44 | 0.99 | 0.83 |
| *Centrostephanus longispinus* | 124331 | 0.88 | 0.74 | 0.65 | 0.46 | 0.94 | 0.28 |
| *Diadema setosum* | 213372 | 0.96 | 0.87 | 0.81 | 0.61 | 0.96 | 0.8 |
| *Brissopsis lyrifera* | 124373 | 0.9 | 0.8 | 0.65 | 0.62 | 0.82 | 0.48 |
| *Brissus unicolor* | 124380 | 0.92 | 0.86 | 0.7 | 0.6 | 0.88 | 0.48 |
| *Echinocardium cordatum* | 124392 | 0.92 | 0.89 | 0.7 | 0.61 | 0.9 | 0.9 |
| *Ova canalifera* | 569394 | 0.92 | 0.87 | 0.72 | 0.63 | 0.89 | 0.87 |
| *Spatangus purpureus* | 124418 | 0.87 | 0.86 | 0.62 | 0.62 | 1 | 0.83 |
| *Paraleptopentacta elongata* | 1474372 | 0.87 | 0.92 | 0.65 | 0.76 | 0.98 | 0.95 |
| *Paraleptopentacta tergestina* | 1474379 | 0.85 | 0.9 | 0.56 | 0.7 | 0.97 | 0.87 |
| *Ocnus planci* | 124647 | 0.86 | 0.96 | 0.58 | 0.8 | 0.98 | 0.89 |
| *Holothuria (Holothuria) tubulosa* | 125182 | 0.84 | 0.89 | 0.53 | 0.63 | 0.99 | 0.9 |
| *Holothuria (Platyperona) sanctori* | 124528 | 0.86 | 0.76 | 0.58 | 0.42 | 0.96 | 0.74 |
| *Holothuria (Roweothuria) poli* | 124525 | 0.89 | 0.87 | 0.63 | 0.62 | 0.97 | 0.92 |
| *Holothuria forskali* | 1670329 | 0.85 | 0.91 | 0.59 | 0.73 | 0.98 | 0.86 |
| *Parastichopus regalis* | 149898 | 0.75 | 0.85 | 0.36 | 0.55 | 0.99 | 0.97 |
| *Amphiura chiajei* | 125073 | 0.91 | 0.92 | 0.7 | 0.71 | 0.84 | 0.77 |
| *Amphiura filiformis* | 125080 | 0.92 | 0.82 | 0.74 | 0.56 | 0.86 | 0.78 |
| *Ophiopsila aranea* | 125049 | 0.91 | 0.92 | 0.64 | 0.72 | 0.9 | 0.87 |
| *Ophiothrix fragilis* | 125131 | 0.77 | 0.87 | 0.44 | 0.66 | 0.98 | 0.88 |
| *Astrospartus mediterraneus* | 124963 | 0.89 | 0.85 | 0.71 | 0.65 | 0.94 | 0.68 |
| *Ophioderma longicaudum* | 1420120 | 0.87 | 0.79 | 0.59 | 0.44 | 0.99 | 0.45 |
| *Ophiura ophiura* | 124929 | 0.82 | 0.8 | 0.51 | 0.53 | 0.98 | 0.95 |
| *Ensis minor* | 140734 | 0.95 | 0.92 | 0.8 | 0.77 | 0.79 | 0.59 |
| *Phaxas pellucidus* | 140737 | 0.95 | 0.98 | 0.77 | 0.89 | 0.95 | 0.81 |
| *Arca noae* | 138788 | 0.85 | 0.89 | 0.54 | 0.69 | 1 | 0.9 |
| *Barbatia barbata* | 138793 | 0.87 | 0.85 | 0.59 | 0.65 | 0.99 | 0.73 |
| *Glycymeris nummaria* | 504509 | 0.94 | 0.81 | 0.74 | 0.6 | 0.88 | 0.37 |
| *Acanthocardia aculeata* | 138990 | 0.92 | 0.8 | 0.68 | 0.49 | 0.85 | 0.78 |
| *Acanthocardia echinata* | 138992 | 0.87 | 0.93 | 0.63 | 0.72 | 0.98 | 0.95 |
| *Acanthocardia tuberculata* | 381057 | 0.89 | 0.89 | 0.62 | 0.67 | 0.98 | 0.92 |
| *Cerastoderma glaucum* | 138999 | 0.92 | 0.92 | 0.73 | 0.75 | 0.97 | 0.74 |
| *Donax semistriatus* | 139601 | 0.94 | 0.92 | 0.77 | 0.69 | 0.95 | 0.82 |
| *Donax trunculus* | 139602 | 0.92 | 0.89 | 0.72 | 0.68 | 0.99 | 0.92 |
| *Donax venustus* | 139603 | 0.95 | 0.83 | 0.79 | 0.54 | 0.9 | 0.18 |
| *Peronaea planata* | 605934 | 0.94 | 0.87 | 0.76 | 0.64 | 0.91 | 0.89 |
| *Lima lima* | 140233 | 0.91 | 0.8 | 0.7 | 0.5 | 0.97 | 0.57 |
| *Limaria hians* | 140235 | 0.9 | 0.83 | 0.66 | 0.62 | 0.91 | 0.63 |
| *Limaria tuberculata* | 140236 | 0.91 | 0.83 | 0.68 | 0.66 | 0.93 | 0.55 |
| *Loripes orbiculatus* | 875379 | 0.9 | 0.9 | 0.67 | 0.72 | 0.95 | 0.22 |
| *Lithophaga lithophaga* | 140459 | 0.87 | 0.84 | 0.6 | 0.57 | 0.98 | 0.93 |
| *Mytilus galloprovincialis* | 140481 | 0.86 | 0.92 | 0.56 | 0.71 | 0.96 | 0.98 |
| *Nucula sulcata* | 140592 | 0.88 | 0.82 | 0.62 | 0.65 | 0.81 | 0.45 |
| *Neopycnodonte cochlear* | 140048 | 0.83 | 0.9 | 0.52 | 0.72 | 1 | 0.88 |
| *Pinctada radiata* | 140890 | 0.92 | 0.9 | 0.71 | 0.74 | 0.98 | 0.85 |
| *Magallana gigas* | 836033 | 0.92 | 0.88 | 0.7 | 0.67 | 0.94 | 0.64 |
| *Ostrea edulis* | 140658 | 0.83 | 0.8 | 0.47 | 0.5 | 0.96 | 0.7 |
| *Pinna nobilis* | 140780 | 0.84 | 0.89 | 0.54 | 0.67 | 0.98 | 0.97 |
| *Pinna rudis* | 140781 | 0.88 | 0.93 | 0.62 | 0.81 | 0.98 | 0.84 |
| *Pteria hirundo* | 140891 | 0.84 | 0.8 | 0.58 | 0.54 | 0.98 | 0.85 |
| *Anomia ephippium* | 138748 | 0.86 | 0.89 | 0.57 | 0.67 | 0.98 | 0.92 |
| *Aequipecten opercularis* | 140687 | 0.9 | 0.85 | 0.66 | 0.56 | 0.96 | 0.77 |
| *Mimachlamys varia* | 236719 | 0.88 | 0.88 | 0.61 | 0.62 | 0.9 | 0.96 |
| *Pecten jacobaeus* | 394429 | 0.86 | 0.88 | 0.6 | 0.6 | 0.92 | 0.92 |
| *Spondylus gaederopus* | 141549 | 0.89 | 0.81 | 0.64 | 0.53 | 0.99 | 0.79 |
| *Mactra stultorum* | 140299 | 0.94 | 0.91 | 0.77 | 0.68 | 0.99 | 0.84 |
| *Spisula subtruncata* | 140302 | 0.93 | 0.97 | 0.76 | 0.88 | 0.97 | 0.96 |
| *Donacilla cornea* | 140350 | 0.96 | 0.89 | 0.82 | 0.65 | 0.96 | 0.76 |
| *Callista chione* | 141906 | 0.92 | 0.89 | 0.72 | 0.67 | 0.88 | 0.95 |
| *Chamelea gallina* | 141907 | 0.91 | 0.85 | 0.67 | 0.56 | 0.99 | 0.74 |
| *Dosinia lupinus* | 141912 | 0.95 | 0.83 | 0.76 | 0.54 | 0.97 | 0.85 |
| *Polititapes aureus* | 246150 | 0.91 | 0.92 | 0.66 | 0.73 | 0.95 | 0.94 |
| *Ruditapes decussatus* | 231749 | 0.92 | 0.93 | 0.7 | 0.81 | 0.81 | 0.83 |
| *Venus nux* | 141935 | 0.94 | 0.94 | 0.78 | 0.78 | 0.91 | 0.91 |
| *Venus verrucosa* | 141936 | 0.87 | 0.93 | 0.59 | 0.76 | 0.99 | 0.97 |
| *Patella caerulea* | 140677 | 0.87 | 0.86 | 0.59 | 0.6 | 0.97 | 0.89 |
| *Patella rustica* | 140683 | 0.87 | 0.86 | 0.6 | 0.54 | 0.98 | 0.75 |
| *Patella ulyssiponensis* | 140684 | 0.9 | 0.83 | 0.66 | 0.54 | 0.94 | 0.87 |
| *Elysia timida* | 139684 | 0.89 | 0.84 | 0.62 | 0.56 | 0.99 | 0.75 |
| *Thuridilla hopei* | 139687 | 0.85 | 0.92 | 0.58 | 0.71 | 0.93 | 0.9 |
| *Aplysia fasciata* | 138755 | 0.87 | 0.87 | 0.62 | 0.64 | 0.87 | 0.78 |
| *Aplysia punctata* | 138758 | 0.89 | 0.82 | 0.65 | 0.6 | 0.83 | 0.58 |
| *Bittium reticulatum* | 139054 | 0.92 | 0.94 | 0.68 | 0.79 | 0.77 | 0.14 |
| *Cerithium vulgatum* | 139066 | 0.88 | 0.91 | 0.62 | 0.69 | 0.98 | 0.91 |
| *Turritellinella tricarinata* | 1381415 | 0.92 | 0.86 | 0.71 | 0.68 | 0.97 | 0.83 |
| *Scaphander lignarius* | 139488 | 0.89 | 0.88 | 0.64 | 0.64 | 0.98 | 0.9 |
| *Haliotis tuberculata* | 140059 | 0.85 | 0.86 | 0.59 | 0.61 | 0.96 | 0.87 |
| *Aporrhais pespelecani* | 138760 | 0.84 | 0.81 | 0.49 | 0.56 | 0.96 | 0.69 |
| *Aporrhais serresiana* | 138761 | 0.9 | 0.95 | 0.67 | 0.8 | 0.97 | 0.94 |
| *Calyptraea chinensis* | 138961 | 0.95 | 0.9 | 0.79 | 0.7 | 0.82 | 0.89 |
| *Luria lurida* | 139499 | 0.88 | 0.8 | 0.65 | 0.56 | 0.97 | 0.38 |
| *Echinolittorina punctata* | 345757 | 0.95 | 0.9 | 0.77 | 0.65 | 0.93 | 0.75 |
| *Melarhaphe neritoides* | 140266 | 0.9 | 0.88 | 0.69 | 0.68 | 0.98 | 0.91 |
| *Euspira fusca* | 140529 | 0.94 | 0.95 | 0.77 | 0.86 | 0.97 | 0.73 |
| *Naticarius stercusmuscarum* | 720574 | 0.92 | 0.82 | 0.69 | 0.54 | 0.97 | 0.49 |
| *Neverita josephinia* | 140549 | 0.94 | 0.9 | 0.79 | 0.66 | 0.95 | 0.8 |
| *Ranella olearium* | 141115 | 0.91 | 0.86 | 0.74 | 0.63 | 0.9 | 0.9 |
| *Conomurex persicus* | 565371 | 0.93 | 0.97 | 0.73 | 0.82 | 0.97 | 0.94 |
| *Tonna galea* | 141687 | 0.9 | 0.79 | 0.71 | 0.46 | 0.96 | 0.83 |
| *Thylacodes arenarius* | 709464 | 0.89 | 0.89 | 0.67 | 0.67 | 0.94 | 0.7 |
| *Xenophora crispa* | 743862 | 0.93 | 0.97 | 0.73 | 0.83 | 0.96 | 0.9 |
| *Columbella rustica* | 139196 | 0.87 | 0.89 | 0.58 | 0.66 | 0.99 | 0.82 |
| *Conus ventricosus* | 428401 | 0.88 | 0.92 | 0.6 | 0.67 | 0.98 | 0.69 |
| *Bolinus brandaris* | 140389 | 0.86 | 0.86 | 0.56 | 0.59 | 0.98 | 0.87 |
| *Hexaplex trunculus* | 140396 | 0.86 | 0.89 | 0.57 | 0.66 | 0.99 | 0.93 |
| *Ocenebra erinaceus* | 140405 | 0.91 | 0.91 | 0.68 | 0.7 | 0.94 | 0.79 |
| *Stramonita haemastoma* | 140417 | 0.92 | 0.74 | 0.7 | 0.37 | 1 | 0.79 |
| *Tritia incrassata* | 876825 | 0.94 | 0.88 | 0.74 | 0.67 | 0.92 | 0.62 |
| *Tritia mutabilis* | 876840 | 0.96 | 0.85 | 0.83 | 0.56 | 0.96 | 0.69 |
| *Pisania striata* | 138924 | 0.86 | 0.84 | 0.59 | 0.6 | 0.94 | 0.81 |
| *Euthria cornea* | 181057 | 0.87 | 0.87 | 0.63 | 0.62 | 0.95 | 0.8 |
| *Felimare orsinii* | 597533 | 0.95 | 0.88 | 0.78 | 0.74 | 0.83 | 0.34 |
| *Felimare picta* | 597522 | 0.83 | 0.81 | 0.51 | 0.58 | 0.99 | 0.69 |
| *Felimare tricolor* | 597530 | 0.91 | 0.75 | 0.73 | 0.44 | 0.96 | 0.49 |
| *Felimare villafranca* | 597536 | 0.91 | 0.9 | 0.66 | 0.81 | 0.96 | 0.47 |
| *Peltodoris atromaculata* | 509315 | 0.86 | 0.77 | 0.59 | 0.52 | 0.97 | 0.41 |
| *Cratena peregrina* | 146862 | 0.86 | 0.82 | 0.57 | 0.64 | 0.91 | 0.58 |
| *Edmundsella pedata* | 1047602 | 0.88 | 0.91 | 0.65 | 0.65 | 0.97 | 0.08 |
| *Flabellina affinis* | 139988 | 0.87 | 0.71 | 0.6 | 0.39 | 0.99 | 0.64 |
| *Paraflabellina ischitana* | 1048137 | 0.92 | 0.8 | 0.76 | 0.65 | 0.87 | 0.63 |
| *Tethys fimbria* | 141643 | 0.84 | 0.94 | 0.58 | 0.78 | 0.96 | 0.9 |
| *Pleurobranchaea meckeli* | 140818 | 0.88 | 0.87 | 0.63 | 0.69 | 0.98 | 0.81 |
| *Calliostoma granulatum* | 141753 | 0.88 | 0.89 | 0.69 | 0.69 | 0.98 | 0.84 |
| *Phorcus articulatus* | 689174 | 0.88 | 0.85 | 0.61 | 0.56 | 0.95 | 0.85 |
| *Phorcus turbinatus* | 689179 | 0.87 | 0.85 | 0.61 | 0.57 | 0.97 | 0.86 |
| *Bolma rugosa* | 141855 | 0.88 | 0.88 | 0.58 | 0.58 | 0.96 | 0.78 |
| *Umbraculum umbraculum* | 141879 | 0.87 | 0.86 | 0.58 | 0.57 | 0.95 | 0.91 |
| *Agelas oroides* | 132454 | 0.86 | 0.77 | 0.59 | 0.44 | 0.96 | 0.45 |
| *Axinella damicornis* | 132472 | 0.88 | 0.87 | 0.63 | 0.66 | 1 | 0.82 |
| *Axinella polypoides* | 132487 | 0.85 | 0.74 | 0.62 | 0.44 | 0.97 | 0.67 |
| *Acanthella acuta* | 132455 | 0.89 | 0.81 | 0.66 | 0.54 | 0.93 | 0.77 |
| *Chondrilla nucula* | 134110 | 0.87 | 0.8 | 0.62 | 0.49 | 0.98 | 0.79 |
| *Chondrosia reniformis* | 134112 | 0.84 | 0.79 | 0.55 | 0.48 | 0.97 | 0.81 |
| *Cliona celata* | 134121 | 0.88 | 0.89 | 0.6 | 0.67 | 0.9 | 0.95 |
| *Cliona viridis* | 134146 | 0.9 | 0.77 | 0.67 | 0.4 | 0.94 | 0.74 |
| *Spirastrella cunctatrix* | 134235 | 0.87 | 0.75 | 0.61 | 0.47 | 0.97 | 0.87 |
| *Dysidea fragilis* | 132324 | 0.85 | 0.83 | 0.56 | 0.52 | 0.97 | 0.54 |
| *Pleraplysilla spinifera* | 132317 | 0.93 | 0.92 | 0.77 | 0.79 | 0.96 | 0.88 |
| *Ircinia oros* | 132356 | 0.9 | 0.91 | 0.69 | 0.76 | 0.93 | 0.94 |
| *Ircinia variabilis* | 132362 | 0.83 | 0.82 | 0.52 | 0.54 | 0.96 | 0.67 |
| *Sarcotragus fasciculatus* | 165081 | 0.88 | 0.81 | 0.63 | 0.56 | 0.98 | 0.84 |
| *Sarcotragus spinosulus* | 165086 | 0.84 | 0.85 | 0.53 | 0.57 | 0.99 | 0.95 |
| *Spongia (Spongia) officinalis* | 165220 | 0.88 | 0.83 | 0.6 | 0.56 | 0.99 | 0.82 |
| *Scalarispongia scalaris* | 165376 | 0.88 | 0.88 | 0.66 | 0.71 | 0.97 | 0.74 |
| *Petrosia (Petrosia) ficiformis* | 166837 | 0.86 | 0.81 | 0.56 | 0.48 | 0.96 | 0.81 |
| *Crambe crambe* | 133445 | 0.86 | 0.81 | 0.61 | 0.48 | 0.96 | 0.78 |
| *Hemimycale columella* | 133543 | 0.9 | 0.87 | 0.67 | 0.63 | 0.99 | 0.67 |
| *Phorbas tenacior* | 133693 | 0.87 | 0.8 | 0.58 | 0.5 | 0.98 | 0.64 |
| *Suberites domuncula* | 134282 | 0.85 | 0.79 | 0.55 | 0.44 | 1 | 0.94 |
| *Tethya aurantium* | 134311 | 0.88 | 0.87 | 0.62 | 0.69 | 0.98 | 0.72 |
| *Aplysina aerophoba* | 133911 | 0.87 | 0.87 | 0.59 | 0.68 | 0.99 | 0.9 |
| *Aplysina cavernicola* | 133913 | 0.88 | 0.88 | 0.64 | 0.71 | 0.99 | 0.81 |

**Table S3** – Variable importance for the fitted species.

| **Name** | **AphiaID** | **SAL_median_** | **T_min_** | **CHL_median_** | **T_median_** | **Vm_median_** | **SAND** | **O2_median_** |
| --- | --- | --- | --- | --- | --- | --- | --- | --- |
| *Heteromastus filiformis* | 129884 | 0.064 | 0.015 | 0.052 | 0.106 | 0 | 0.023 | 0.07 |
| *Notomastus latericeus* | 129898 | 0.012 | 0.177 | 0.003 | 0.037 | 0.004 | 0.01 | 0.353 |
| *Myriochele heeri* | 130542 | 0.035 | 0.441 | 0.162 | 0.323 | 0 | 0.183 | 0.107 |
| *Owenia fusiformis* | 130544 | 0.04 | 0.276 | 0.048 | 0.965 | 0.027 | 0.179 | 0.315 |
| *Hermodice carunculata* | 129831 | 0.027 | 0.026 | 0.032 | 0.749 | 0.034 | 0.032 | 0.025 |
| *Bonellia viridis* | 110363 | 0.082 | 0.053 | 0.083 | 0.075 | 0.012 | 0.087 | 0.067 |
| *Eunice vittata* | 130067 | 0.037 | 0.343 | 0.058 | 0.595 | 0.029 | 0.091 | 0.145 |
| *Lumbrineris latreilli* | 130248 | 0.061 | 0.227 | 0.204 | 0.148 | 0 | 0.035 | 0.229 |
| *Drilonereis filum* | 129856 | 0.293 | 0.143 | 0.098 | 0.689 | 0.052 | 0.179 | 0.011 |
| *Aponuphis bilineata* | 130452 | 0.136 | 0.156 | 0.052 | 0.747 | 0.013 | 0.18 | 0.209 |
| *Hyalinoecia tubicola* | 130464 | 0.09 | 0.251 | 0.011 | 0.139 | 0.004 | 0.079 | 0.127 |
| *Aphrodita aculeata* | 129840 | 0.082 | 0.022 | 0.139 | 0.022 | 0.003 | 0.01 | 0.113 |
| *Laetmonice hystrix* | 129845 | 0.12 | 0.109 | 0.053 | 0.221 | 0.01 | 0.244 | 0.111 |
| *Glycera alba* | 130116 | 0.088 | 0.042 | 0.098 | 0.172 | 0.002 | 0.018 | 0.069 |
| *Glycera tridactyla* | 130130 | 0.189 | 0.081 | 0.003 | 0.491 | 0.012 | 0.008 | 0.079 |
| *Glycera unicornis* | 130131 | 0.123 | 0.244 | 0.025 | 0.336 | 0.001 | 0.057 | 0.061 |
| *Goniada maculata* | 130140 | 0.107 | 0.107 | 0.012 | 0.068 | 0 | 0.012 | 0.157 |
| *Nephtys hombergii* | 130359 | 0.036 | 0.122 | 0.09 | 0.649 | 0.001 | 0.082 | 0.154 |
| *Sigalion mathildae* | 131072 | 0.047 | 0.078 | 0.024 | 0.278 | 0.004 | 0.004 | 0.022 |
| *Sabella pavonina* | 130967 | 0.042 | 0.18 | 0.057 | 0.351 | 0.001 | 0.174 | 0.158 |
| *Sabella spallanzanii* | 130969 | 0.025 | 0.082 | 0.022 | 0.124 | 0.004 | 0.05 | 0.161 |
| *Protula intestinum* | 131032 | 0.035 | 0.019 | 0.11 | 0.078 | 0.004 | 0.05 | 0.086 |
| *Protula tubularia* | 131035 | 0.084 | 0.138 | 0.074 | 0.172 | 0.009 | 0.142 | 0.216 |
| *Serpula vermicularis* | 131051 | 0.078 | 0.017 | 0.073 | 0.08 | 0.023 | 0.076 | 0.118 |
| *Laonice cirrata* | 131128 | 0.369 | 0.009 | 0.097 | 0.577 | 0.019 | 0.178 | 0.065 |
| *Prionospio steenstrupi* | 131164 | 0.309 | 0.326 | 0.046 | 0.061 | 0.03 | 0.064 | 0.11 |
| *Ampharete grubei* | 152272 | 0.048 | 0.464 | 0.036 | 0.127 | 0.001 | 0.084 | 0.487 |
| *Melinna palmata* | 129808 | 0.04 | 0.342 | 0.048 | 0.264 | 0.001 | 0.032 | 0.126 |
| *Sternaspis scutata* | 131242 | 0.057 | 0.169 | 0.128 | 0.033 | 0.002 | 0.012 | 0.105 |
| *Ampelisca sarsi* | 101923 | 0.014 | 0.188 | 0.303 | 0.27 | 0 | 0.236 | 0.149 |
| *Ampelisca typica* | 101933 | 0.02 | 0.406 | 0.296 | 0.159 | 0.003 | 0.373 | 0.156 |
| *Athanas nitescens* | 107486 | 0.652 | 0.016 | 0.118 | 0.058 | 0.011 | 0.054 | 0.061 |
| *Calocaris macandreae* | 107726 | 0.142 | 0.276 | 0.044 | 0.53 | 0 | 0.087 | 0.717 |
| *Calappa granulata* | 107268 | 0.011 | 0.027 | 0.067 | 0.201 | 0.013 | 0.053 | 0.223 |
| *Procambarus clarkii* | 465540 | 0.049 | 0.036 | 0.093 | 0.071 | 0.002 | 0.002 | 0.042 |
| *Carcinus aestuarii* | 107380 | 0.108 | 0.009 | 0.032 | 0.09 | 0.025 | 0.037 | 0.053 |
| *Aegaeon cataphractus* | 107548 | 0.031 | 0.267 | 0.533 | 0.052 | 0.014 | 0.006 | 0.135 |
| *Pontophilus spinosus* | 107564 | 0.094 | 0.368 | 0 | 0.532 | 0 | 0.071 | 0.71 |
| *Clibanarius erythropus* | 107196 | 0.035 | 0.005 | 0.071 | 0.181 | 0.034 | 0.029 | 0.042 |
| *Dardanus arrosor* | 107197 | 0.186 | 0.141 | 0.133 | 0.071 | 0.005 | 0.027 | 0.309 |
| *Dardanus calidus* | 107198 | 0.089 | 0.056 | 0.014 | 0.267 | 0.032 | 0.071 | 0.134 |
| *Diogenes pugilator* | 107199 | 0.047 | 0.012 | 0.05 | 0.191 | 0.022 | 0.009 | 0.111 |
| *Paguristes eremita* | 107200 | 0.115 | 0.023 | 0.034 | 0.185 | 0 | 0.059 | 0.039 |
| *Medorippe lanata* | 107288 | 0.016 | 0.305 | 0.194 | 0.314 | 0.004 | 0.007 | 0.197 |
| *Dromia personata* | 107258 | 0.038 | 0.057 | 0.076 | 0.043 | 0 | 0.081 | 0.172 |
| *Pisa armata* | 107353 | 0.163 | 0.125 | 0.028 | 0.07 | 0.009 | 0.075 | 0.262 |
| *Eriphia verrucosa* | 107409 | 0.094 | 0.007 | 0.018 | 0.344 | 0.048 | 0.057 | 0.06 |
| *Ethusa mascarone* | 107283 | 0.062 | 0.03 | 0.386 | 0.067 | 0.022 | 0.011 | 0.075 |
| *Galathea intermedia* | 107150 | 0.638 | 0.002 | 0.101 | 0.05 | 0.023 | 0.003 | 0.113 |
| *Galathea strigosa* | 107155 | 0.096 | 0.221 | 0.022 | 0.104 | 0.003 | 0.047 | 0.175 |
| *Geryon longipes* | 107373 | 0.09 | 0.362 | 0.004 | 0.749 | 0 | 0.029 | 0.376 |
| *Goneplax rhomboides* | 107292 | 0.068 | 0.163 | 0.173 | 0.375 | 0.003 | 0.095 | 0.504 |
| *Pachygrapsus marmoratus* | 107455 | 0.12 | 0.084 | 0.027 | 0.329 | 0.034 | 0.048 | 0.093 |
| *Homola barbata* | 107262 | 0.069 | 0.048 | 0.13 | 0.166 | 0.004 | 0.037 | 0.316 |
| *Paromola cuvieri* | 107264 | 0.084 | 0.191 | 0.01 | 0.69 | 0 | 0.035 | 0.437 |
| *Inachus communissimus* | 107326 | 0.016 | 0.299 | 0.043 | 0.402 | 0.099 | 0.035 | 0.27 |
| *Inachus dorsettensis* | 107327 | 0.361 | 0.079 | 0.016 | 0.122 | 0.002 | 0.017 | 0.309 |
| *Inachus phalangium* | 107333 | 0.071 | 0.199 | 0.002 | 0.401 | 0 | 0.235 | 0.477 |
| *Inachus thoracicus* | 107334 | 0.314 | 0.438 | 0.066 | 0.172 | 0.005 | 0.07 | 0.401 |
| *Macropodia linaresi* | 107341 | 0.667 | 0.153 | 0.015 | 0.173 | 0.027 | 0.027 | 0.249 |
| *Macropodia longirostris* | 107343 | 0.077 | 0.453 | 0.004 | 0.568 | 0 | 0.046 | 0.531 |
| *Macropodia rostrata* | 107345 | 0.034 | 0.019 | 0.068 | 0.034 | 0.024 | 0.048 | 0.393 |
| *Macropodia tenuirostris* | 107346 | 0.111 | 0.065 | 0.081 | 0.103 | 0.002 | 0.001 | 0.356 |
| *Latreillia elegans* | 107265 | 0.039 | 0.203 | 0.014 | 0.763 | 0 | 0.076 | 0.23 |
| *Lysmata seticaudata* | 107528 | 0.316 | 0.012 | 0.049 | 0.02 | 0.012 | 0.107 | 0.05 |
| *Maja crispata* | 107348 | 0.228 | 0.01 | 0.108 | 0.069 | 0.05 | 0.162 | 0.014 |
| *Maja squinado* | 107350 | 0.027 | 0.031 | 0.005 | 0.169 | 0.01 | 0.056 | 0.446 |
| *Iridonida speciosa* | 1606714 | 0.072 | 0.143 | 0.017 | 0.23 | 0.003 | 0.013 | 0.845 |
| *Munida intermedia* | 107157 | 0.068 | 0.055 | 0.052 | 0.626 | 0 | 0.011 | 0.516 |
| *Munida tenuimana* | 107166 | 0.089 | 0.237 | 0.054 | 0.602 | 0.002 | 0.047 | 0.714 |
| *Homarus gammarus* | 107253 | 0.13 | 0.025 | 0.076 | 0.018 | 0 | 0.01 | 0.029 |
| *Pagurus alatus* | 107230 | 0.131 | 0.395 | 0.003 | 0.292 | 0.002 | 0.011 | 0.624 |
| *Pagurus anachoretus* | 107231 | 0.451 | 0.02 | 0.013 | 0.027 | 0.06 | 0.062 | 0.037 |
| *Pagurus cuanensis* | 107235 | 0.108 | 0.067 | 0.262 | 0.072 | 0.007 | 0.013 | 0.348 |
| *Pagurus excavatus* | 107236 | 0.068 | 0.314 | 0.344 | 0.315 | 0.002 | 0.024 | 0.346 |
| *Pagurus prideaux* | 107239 | 0.193 | 0.066 | 0.046 | 0.059 | 0.015 | 0.088 | 0.388 |
| *Palaemon elegans* | 107614 | 0.079 | 0.021 | 0.008 | 0.186 | 0.086 | 0.055 | 0.129 |
| *Palaemon serratus* | 107616 | 0.146 | 0.006 | 0.089 | 0.052 | 0.018 | 0.044 | 0.039 |
| *Palinurus elephas* | 107703 | 0.344 | 0.109 | 0.028 | 0.211 | 0.034 | 0.07 | 0.175 |
| *Chlorotocus crassicornis* | 107642 | 0.061 | 0.092 | 0.004 | 0.905 | 0 | 0.029 | 0.598 |
| *Plesionika acanthonotus* | 107654 | 0.087 | 0.117 | 0.001 | 0.549 | 0.001 | 0.018 | 0.485 |
| *Plesionika antigai* | 107655 | 0.085 | 0.108 | 0.013 | 0.637 | 0.002 | 0.037 | 0.634 |
| *Plesionika edwardsii* | 107656 | 0.073 | 0.106 | 0.068 | 0.648 | 0.005 | 0.014 | 0.453 |
| *Plesionika gigliolii* | 107659 | 0.062 | 0.077 | 0.021 | 0.464 | 0.009 | 0.018 | 0.447 |
| *Plesionika heterocarpus* | 107660 | 0.026 | 0.09 | 0.046 | 0.364 | 0.013 | 0.019 | 0.208 |
| *Plesionika martia* | 107661 | 0.031 | 0.265 | 0.067 | 0.556 | 0.003 | 0.023 | 0.222 |
| *Plesionika narval* | 107662 | 0.041 | 0.205 | 0.029 | 0.215 | 0.01 | 0.034 | 0.489 |
| *Spinolambrus macrochelos* | 442337 | 0.037 | 0.167 | 0.007 | 0.687 | 0.002 | 0.049 | 0.158 |
| *Penaeus kerathurus* | 246388 | 0.03 | 0.077 | 0.017 | 0.35 | 0.008 | 0.017 | 0.072 |
| *Pilumnus hirtellus* | 107418 | 0.083 | 0.003 | 0.066 | 0.065 | 0 | 0.004 | 0.054 |
| *Pilumnus spinifer* | 107420 | 0.219 | 0.062 | 0.279 | 0.025 | 0.154 | 0.06 | 0.154 |
| *Pilumnus villosissimus* | 107421 | 0.138 | 0.102 | 0.192 | 0.021 | 0.001 | 0.013 | 0.08 |
| *Bathynectes maravigna* | 107377 | 0.077 | 0.143 | 0.006 | 0.477 | 0.001 | 0.014 | 0.695 |
| *Polybius depurator* | 1750291 | 0.063 | 0.075 | 0.08 | 0.272 | 0.006 | 0.028 | 0.638 |
| *Polybius vernalis* | 1750288 | 0.034 | 0.08 | 0.026 | 0.324 | 0.002 | 0.004 | 0.01 |
| *Macropipus tuberculatus* | 107397 | 0.034 | 0.065 | 0.023 | 0.518 | 0.01 | 0.025 | 0.403 |
| *Polycheles typhlops* | 107696 | 0.051 | 0.33 | 0.018 | 0.533 | 0.003 | 0.009 | 0.233 |
| *Processa canaliculata* | 107682 | 0.052 | 0.274 | 0.037 | 0.316 | 0.001 | 0.02 | 0.295 |
| *Processa nouveli* | 107689 | 0.133 | 0.214 | 0 | 0.706 | 0 | 0.024 | 0.342 |
| *Scyllarides latus* | 107708 | 0.104 | 0.116 | 0.234 | 0.048 | 0.007 | 0.06 | 0.123 |
| *Solenocera membranacea* | 107120 | 0.072 | 0.127 | 0.026 | 0.61 | 0 | 0.109 | 0.53 |
| *Stenopus spinosus* | 107721 | 0.042 | 0.02 | 0.331 | 0.015 | 0.004 | 0.04 | 0.055 |
| *Monodaeus couchii* | 241154 | 0.093 | 0.154 | 0.01 | 0.527 | 0 | 0.038 | 0.819 |
| *Xantho poressa* | 107442 | 0.256 | 0.027 | 0.068 | 0.069 | 0.092 | 0.109 | 0.047 |
| *Natatolana borealis* | 118859 | 0.058 | 0.138 | 0.019 | 0.251 | 0.003 | 0.028 | 0.353 |
| *Rissoides pallidus* | 136136 | 0.056 | 0.198 | 0.027 | 0.338 | 0.012 | 0.049 | 0.32 |
| *Squilla mantis* | 136137 | 0.014 | 0.031 | 0.082 | 0.171 | 0 | 0.002 | 0.072 |
| *Apseudopsis latreillii* | 247077 | 0.246 | 0.276 | 0.015 | 0.065 | 0.018 | 0.052 | 0.529 |
| *Adeonella calveti* | 111055 | 0.05 | 0.139 | 0.15 | 0.637 | 0.024 | 0.115 | 0.31 |
| *Reptadeonella violacea* | 111061 | 0.032 | 0.257 | 0.13 | 0.146 | 0 | 0.066 | 0.12 |
| *Pentapora fascialis* | 111082 | 0.14 | 0.04 | 0.073 | 0.537 | 0.026 | 0.032 | 0.168 |
| *Schizomavella (Schizomavella) mamillata* | 862797 | 0.138 | 0.19 | 0.024 | 0.206 | 0.026 | 0.07 | 0.078 |
| *Electra posidoniae* | 111356 | 0.065 | 0.033 | 0.017 | 0.467 | 0.041 | 0.19 | 0.248 |
| *Myriapora truncata* | 111435 | 0.091 | 0.007 | 0.069 | 0.106 | 0.047 | 0.125 | 0.04 |
| *Reteporella grimaldii* | 111453 | 0.064 | 0.187 | 0.012 | 0.603 | 0.005 | 0.057 | 0.114 |
| *Schizobrachiella sanguinea* | 111522 | 0.115 | 0.004 | 0.077 | 0.029 | 0.008 | 0.012 | 0.04 |
| *Smittina cervicornis* | 111551 | 0.129 | 0.15 | 0.013 | 0.186 | 0.026 | 0.122 | 0.201 |
| *Clavelina lepadiformis* | 103552 | 0.081 | 0.025 | 0.15 | 0.185 | 0.01 | 0.078 | 0.027 |
| *Diazona violacea* | 103733 | 0.214 | 0.287 | 0.003 | 0.025 | 0.002 | 0.083 | 0.286 |
| *Aplidium conicum* | 103641 | 0.06 | 0.028 | 0.016 | 0.204 | 0.002 | 0.045 | 0.042 |
| *Aplidium elegans* | 103643 | 0.591 | 0.145 | 0.02 | 0.104 | 0.05 | 0.087 | 0.028 |
| *Ascidia mentula* | 103710 | 0.112 | 0.051 | 0.009 | 0.306 | 0.01 | 0.017 | 0.314 |
| *Ascidia virginea* | 103717 | 0.027 | 0.133 | 0.047 | 0.054 | 0.001 | 0.041 | 0.453 |
| *Ascidiella scabra* | 103719 | 0.047 | 0.251 | 0.019 | 0.132 | 0.001 | 0.05 | 0.724 |
| *Phallusia mammillata* | 103724 | 0.007 | 0.224 | 0.168 | 0.281 | 0 | 0.021 | 0.362 |
| *Ciona intestinalis* | 103732 | 0.137 | 0.049 | 0.282 | 0.043 | 0.013 | 0.068 | 0.047 |
| *Molgula appendiculata* | 103771 | 0.877 | 0.049 | 0.043 | 0.172 | 0.064 | 0 | 0.044 |
| *Halocynthia papillosa* | 103827 | 0.091 | 0.187 | 0.012 | 0.371 | 0.022 | 0.091 | 0.12 |
| *Microcosmus sabatieri* | 103844 | 0.261 | 0.052 | 0.034 | 0.096 | 0.012 | 0.004 | 0.261 |
| *Microcosmus vulgaris* | 103846 | 0.123 | 0.264 | 0.004 | 0.168 | 0.01 | 0.022 | 0.868 |
| *Botryllus schlosseri* | 103862 | 0.059 | 0.013 | 0.029 | 0.071 | 0.006 | 0.02 | 0.115 |
| *Actinia mediterranea* | 854459 | 0.059 | 0.066 | 0.008 | 0.427 | 0.003 | 0.035 | 0.157 |
| *Anemonia viridis* | 100808 | 0.093 | 0.005 | 0.011 | 0.099 | 0.03 | 0.137 | 0.167 |
| *Cribrinopsis crassa* | 100823 | 0.062 | 0.401 | 0.139 | 0.142 | 0.002 | 0.164 | 0.192 |
| *Aiptasia mutabilis* | 100859 | 0.076 | 0.014 | 0.009 | 0.337 | 0.042 | 0.042 | 0.093 |
| *Actinauge richardi* | 100930 | 0.107 | 0.132 | 0.058 | 0.177 | 0.002 | 0.094 | 0.677 |
| *Calliactis palliata* | 1658483 | 0.034 | 0.097 | 0.173 | 0.137 | 0.014 | 0.158 | 0.346 |
| *Calliactis parasitica* | 100946 | 0.06 | 0.007 | 0.026 | 0.125 | 0.023 | 0.039 | 0.307 |
| *Cereus pedunculatus* | 100987 | 0.07 | 0.412 | 0.005 | 0.265 | 0.009 | 0.066 | 0.16 |
| *Cerianthus membranacea* | 856035 | 0.054 | 0.283 | 0.142 | 0.094 | 0 | 0.064 | 0.106 |
| *Cerianthus membranaceus* | 101011 | 0.136 | 0.045 | 0.084 | 0.035 | 0.006 | 0.004 | 0.274 |
| *Caryophyllia (Caryophyllia) inornata* | 135141 | 0.134 | 0.395 | 0.113 | 0.2 | 0.005 | 0.092 | 0.191 |
| *Caryophyllia (Caryophyllia) smithii* | 135144 | 0.089 | 0.049 | 0.164 | 0.064 | 0.013 | 0.029 | 0.081 |
| *Cladocora caespitosa* | 135146 | 0.158 | 0.057 | 0.015 | 0.174 | 0.049 | 0.032 | 0.073 |
| *Astroides calycularis* | 135178 | 0.468 | 0.083 | 0.25 | 0.818 | 0.011 | 0.209 | 0.174 |
| *Balanophyllia (Balanophyllia) europaea* | 135180 | 0.027 | 0.27 | 0.008 | 0.488 | 0.001 | 0.055 | 0.086 |
| *Leptopsammia pruvoti* | 135193 | 0.083 | 0.053 | 0.026 | 0.233 | 0.011 | 0.033 | 0.055 |
| *Oculina patagonica* | 135210 | 0.032 | 0.159 | 0.393 | 0.269 | 0.038 | 0.069 | 0.104 |
| *Parazoanthus axinellae* | 101055 | 0.518 | 0.015 | 0.047 | 0.063 | 0.04 | 0.058 | 0.171 |
| *Paramuricea clavata* | 125387 | 0.025 | 0.107 | 0.023 | 0.276 | 0.012 | 0.124 | 0.244 |
| *Alcyonium acaule* | 125331 | 0.386 | 0.002 | 0.015 | 0.084 | 0.011 | 0.032 | 0.054 |
| *Alcyonium coralloides* | 125332 | 0.042 | 0.162 | 0.034 | 0.354 | 0.003 | 0.208 | 0.26 |
| *Alcyonium palmatum* | 125334 | 0.035 | 0.012 | 0.094 | 0.085 | 0.001 | 0.001 | 0.147 |
| *Eunicella cavolini* | 125361 | 0.083 | 0.212 | 0.005 | 0.411 | 0.008 | 0.116 | 0.192 |
| *Eunicella singularis* | 125365 | 0.516 | 0.049 | 0.004 | 0.088 | 0.004 | 0.143 | 0.009 |
| *Eunicella verrucosa* | 125366 | 0.079 | 0.302 | 0.049 | 0.49 | 0.001 | 0.022 | 0.046 |
| *Leptogorgia sarmentosa* | 125369 | 0.072 | 0.322 | 0.627 | 0.341 | 0 | 0.148 | 0.185 |
| *Corallium rubrum* | 125416 | 0.016 | 0.26 | 0.09 | 0.634 | 0.001 | 0.209 | 0.351 |
| *Funiculina quadrangularis* | 128506 | 0.062 | 0.232 | 0.001 | 0.366 | 0.001 | 0.005 | 0.544 |
| *Isidella elongata* | 125373 | 0.045 | 0.467 | 0.008 | 0.185 | 0.001 | 0.092 | 0.035 |
| *Pennatula phosphorea* | 128517 | 0.041 | 0.138 | 0.004 | 0.062 | 0.001 | 0.007 | 0.68 |
| *Pennatula rubra* | 128519 | 0.04 | 0.199 | 0.159 | 0.123 | 0 | 0.028 | 0.51 |
| *Pteroeides griseum* | 181504 | 0.085 | 0.055 | 0.13 | 0.098 | 0.001 | 0.066 | 0.331 |
| *Veretillum cynomorium* | 128536 | 0.114 | 0.388 | 0.322 | 0.202 | 0.005 | 0.035 | 0.56 |
| *Coscinasterias tenuispina* | 123795 | 0.01 | 0.004 | 0.1 | 0.113 | 0.021 | 0.054 | 0.086 |
| *Marthasterias glacialis* | 123803 | 0.045 | 0.147 | 0.089 | 0.091 | 0.002 | 0.055 | 0.248 |
| *Astropecten aranciacus* | 123856 | 0.022 | 0.037 | 0.02 | 0.121 | 0.053 | 0.078 | 0.05 |
| *Astropecten bispinosus* | 123859 | 0.023 | 0.039 | 0.012 | 0.314 | 0.032 | 0.007 | 0.164 |
| *Astropecten irregularis* | 123867 | 0.059 | 0.093 | 0.027 | 0.205 | 0.003 | 0.025 | 0.528 |
| *Astropecten jonstoni* | 123868 | 0.01 | 0.038 | 0.001 | 0.253 | 0.043 | 0.084 | 0.435 |
| *Astropecten platyacanthus* | 123876 | 0.063 | 0.021 | 0.017 | 0.142 | 0.058 | 0.013 | 0.271 |
| *Astropecten spinulosus* | 123878 | 0.023 | 0.009 | 0.017 | 0.041 | 0.017 | 0.013 | 0.309 |
| *Tethyaster subinermis* | 123913 | 0.164 | 0.022 | 0.021 | 0.122 | 0.061 | 0.034 | 0.282 |
| *Luidia ciliaris* | 123920 | 0.082 | 0.111 | 0.032 | 0.101 | 0.014 | 0.063 | 0.29 |
| *Luidia sarsii* | 123922 | 0.109 | 0.342 | 0.302 | 0.021 | 0.001 | 0.004 | 0.052 |
| *Echinaster (Echinaster) sepositus* | 125161 | 0.112 | 0.002 | 0.06 | 0.055 | 0.021 | 0.08 | 0.071 |
| *Anseropoda placenta* | 123985 | 0.254 | 0.007 | 0.025 | 0.06 | 0.001 | 0.052 | 0.074 |
| *Chaetaster longipes* | 124004 | 0.186 | 0.215 | 0.056 | 0.028 | 0.004 | 0.105 | 0.108 |
| *Peltaster placenta* | 124055 | 0.007 | 0.018 | 0.092 | 0.095 | 0.001 | 0.026 | 0.127 |
| *Hacelia attenuata* | 124094 | 0.048 | 0.049 | 0.005 | 0.108 | 0.005 | 0.115 | 0.489 |
| *Ophidiaster ophidianus* | 124101 | 0.027 | 0.081 | 0.028 | 0.4 | 0.027 | 0.036 | 0.026 |
| *Arbacia lixula* | 124249 | 0.005 | 0.015 | 0.061 | 0.257 | 0.01 | 0.05 | 0.043 |
| *Echinus melo* | 124294 | 0.058 | 0.188 | 0.116 | 0.397 | 0.035 | 0.101 | 0.036 |
| *Gracilechinus acutus* | 532031 | 0.072 | 0.107 | 0.031 | 0.423 | 0 | 0.103 | 0.489 |
| *Paracentrotus lividus* | 124316 | 0.065 | 0.008 | 0.027 | 0.167 | 0.015 | 0.041 | 0.055 |
| *Psammechinus microtuberculatus* | 124318 | 0.282 | 0.138 | 0.252 | 0.188 | 0.033 | 0.059 | 0.208 |
| *Sphaerechinus granularis* | 124427 | 0.119 | 0.001 | 0.021 | 0.051 | 0.044 | 0.056 | 0.192 |
| *Stylocidaris affinis* | 124268 | 0.014 | 0.21 | 0.026 | 0.227 | 0.005 | 0.179 | 0.194 |
| *Centrostephanus longispinus* | 124331 | 0.04 | 0.309 | 0.001 | 0.332 | 0 | 0.06 | 0.41 |
| *Diadema setosum* | 213372 | 0.438 | 0.019 | 0.063 | 0.342 | 0.025 | 0.006 | 0.056 |
| *Brissopsis lyrifera* | 124373 | 0.022 | 0.117 | 0.173 | 0.004 | 0.013 | 0.005 | 0.068 |
| *Brissus unicolor* | 124380 | 0.035 | 0.042 | 0.055 | 0.204 | 0.014 | 0.006 | 0.138 |
| *Echinocardium cordatum* | 124392 | 0.022 | 0.05 | 0.04 | 0.328 | 0.001 | 0.006 | 0.038 |
| *Ova canalifera* | 569394 | 0.062 | 0.071 | 0.015 | 0.119 | 0.002 | 0.038 | 0.526 |
| *Spatangus purpureus* | 124418 | 0.2 | 0.202 | 0.022 | 0.273 | 0.002 | 0.092 | 0.129 |
| *Paraleptopentacta elongata* | 1474372 | 0.053 | 0.151 | 0.117 | 0.026 | 0.002 | 0.031 | 0.18 |
| *Paraleptopentacta tergestina* | 1474379 | 0.226 | 0.011 | 0.06 | 0.115 | 0.004 | 0.014 | 0.043 |
| *Ocnus planci* | 124647 | 0.105 | 0.111 | 0.003 | 0.053 | 0.003 | 0.001 | 0.451 |
| *Holothuria (Holothuria) tubulosa* | 125182 | 0.214 | 0.02 | 0.023 | 0.285 | 0.073 | 0.039 | 0.046 |
| *Holothuria (Platyperona) sanctori* | 124528 | 0.016 | 0.005 | 0.049 | 0.066 | 0.019 | 0.085 | 0.146 |
| *Holothuria (Roweothuria) poli* | 124525 | 0.136 | 0.011 | 0.005 | 0.23 | 0.067 | 0.025 | 0.128 |
| *Holothuria forskali* | 1670329 | 0.102 | 0.106 | 0.144 | 0.089 | 0.006 | 0.102 | 0.23 |
| *Parastichopus regalis* | 149898 | 0.039 | 0.1 | 0.053 | 0.226 | 0.004 | 0.017 | 0.115 |
| *Amphiura chiajei* | 125073 | 0.035 | 0.046 | 0.093 | 0.161 | 0.001 | 0.014 | 0.043 |
| *Amphiura filiformis* | 125080 | 0.051 | 0.073 | 0.099 | 0.046 | 0 | 0.012 | 0.028 |
| *Ophiopsila aranea* | 125049 | 0.088 | 0.247 | 0.131 | 0.437 | 0.015 | 0.069 | 0.177 |
| *Ophiothrix fragilis* | 125131 | 0.204 | 0.007 | 0.031 | 0.06 | 0.033 | 0.02 | 0.061 |
| *Astrospartus mediterraneus* | 124963 | 0.434 | 0.103 | 0.056 | 0.233 | 0.004 | 0.028 | 0.083 |
| *Ophioderma longicaudum* | 1420120 | 0.118 | 0.058 | 0.022 | 0.119 | 0.053 | 0.087 | 0.136 |
| *Ophiura ophiura* | 124929 | 0.085 | 0.007 | 0.009 | 0.087 | 0.009 | 0.048 | 0.216 |
| *Ensis minor* | 140734 | 0.041 | 0.005 | 0.048 | 0.296 | 0.005 | 0.015 | 0.008 |
| *Phaxas pellucidus* | 140737 | 0.042 | 0.213 | 0.366 | 0.237 | 0.005 | 0.112 | 0.212 |
| *Arca noae* | 138788 | 0.147 | 0.038 | 0.022 | 0.268 | 0.04 | 0.13 | 0.067 |
| *Barbatia barbata* | 138793 | 0.058 | 0.016 | 0.045 | 0.143 | 0.088 | 0.126 | 0.074 |
| *Glycymeris nummaria* | 504509 | 0.29 | 0.059 | 0.005 | 0.39 | 0.045 | 0.022 | 0.054 |
| *Acanthocardia aculeata* | 138990 | 0.068 | 0.001 | 0.046 | 0.098 | 0.001 | 0.001 | 0.071 |
| *Acanthocardia echinata* | 138992 | 0.055 | 0.034 | 0.15 | 0.063 | 0.017 | 0.007 | 0.391 |
| *Acanthocardia tuberculata* | 381057 | 0.15 | 0.019 | 0.015 | 0.27 | 0.025 | 0.026 | 0.058 |
| *Cerastoderma glaucum* | 138999 | 0.088 | 0.034 | 0.031 | 0.378 | 0.079 | 0.03 | 0.073 |
| *Donax semistriatus* | 139601 | 0.141 | 0.15 | 0.023 | 0.6 | 0.04 | 0.039 | 0.12 |
| *Donax trunculus* | 139602 | 0.039 | 0.036 | 0.085 | 0.636 | 0.016 | 0.018 | 0.058 |
| *Donax venustus* | 139603 | 0.08 | 0.01 | 0.079 | 0.36 | 0.005 | 0.001 | 0.037 |
| *Peronaea planata* | 605934 | 0.095 | 0.01 | 0.005 | 0.453 | 0.017 | 0.065 | 0.027 |
| *Lima lima* | 140233 | 0.039 | 0.138 | 0.065 | 0.226 | 0.006 | 0.314 | 0.192 |
| *Limaria hians* | 140235 | 0.059 | 0.006 | 0.126 | 0.023 | 0.011 | 0.035 | 0.125 |
| *Limaria tuberculata* | 140236 | 0.033 | 0.009 | 0.017 | 0.219 | 0.014 | 0.004 | 0.144 |
| *Loripes orbiculatus* | 875379 | 0.038 | 0.014 | 0.108 | 0.211 | 0.018 | 0.068 | 0.005 |
| *Lithophaga lithophaga* | 140459 | 0.025 | 0.015 | 0.038 | 0.034 | 0.051 | 0.075 | 0.231 |
| *Mytilus galloprovincialis* | 140481 | 0.026 | 0.067 | 0.01 | 0.172 | 0.015 | 0.008 | 0.073 |
| *Nucula sulcata* | 140592 | 0.154 | 0.053 | 0.077 | 0.011 | 0.002 | 0.004 | 0.027 |
| *Neopycnodonte cochlear* | 140048 | 0.022 | 0.186 | 0.2 | 0.049 | 0.001 | 0.033 | 0.082 |
| *Pinctada radiata* | 140890 | 0.099 | 0.053 | 0.03 | 0.358 | 0.139 | 0.045 | 0.026 |
| *Magallana gigas* | 836033 | 0.095 | 0.057 | 0.074 | 0.143 | 0.004 | 0.006 | 0.11 |
| *Ostrea edulis* | 140658 | 0.032 | 0.051 | 0.029 | 0.073 | 0.003 | 0.008 | 0.103 |
| *Pinna nobilis* | 140780 | 0.036 | 0.137 | 0.016 | 0.235 | 0 | 0.06 | 0.299 |
| *Pinna rudis* | 140781 | 0.171 | 0.08 | 0.278 | 0.111 | 0.007 | 0.166 | 0.13 |
| *Pteria hirundo* | 140891 | 0.078 | 0.078 | 0.015 | 0.081 | 0.012 | 0.004 | 0.621 |
| *Anomia ephippium* | 138748 | 0.155 | 0.024 | 0.014 | 0.178 | 0.006 | 0.033 | 0.053 |
| *Aequipecten opercularis* | 140687 | 0.06 | 0.101 | 0.024 | 0.112 | 0.001 | 0.028 | 0.34 |
| *Mimachlamys varia* | 236719 | 0.059 | 0.001 | 0.087 | 0.034 | 0.003 | 0.019 | 0.046 |
| *Pecten jacobaeus* | 394429 | 0.048 | 0.001 | 0.049 | 0.037 | 0.002 | 0.011 | 0.131 |
| *Spondylus gaederopus* | 141549 | 0.041 | 0.038 | 0.036 | 0.227 | 0.063 | 0.138 | 0.138 |
| *Mactra stultorum* | 140299 | 0.075 | 0.057 | 0.029 | 0.933 | 0.034 | 0.01 | 0.115 |
| *Spisula subtruncata* | 140302 | 0.111 | 0.028 | 0.039 | 0.203 | 0.007 | 0.01 | 0.035 |
| *Donacilla cornea* | 140350 | 0.037 | 0.025 | 0.048 | 0.558 | 0.024 | 0.015 | 0.034 |
| *Callista chione* | 141906 | 0.072 | 0.009 | 0.015 | 0.092 | 0.013 | 0.006 | 0.229 |
| *Chamelea gallina* | 141907 | 0.204 | 0.013 | 0.007 | 0.555 | 0.037 | 0.039 | 0.003 |
| *Dosinia lupinus* | 141912 | 0.029 | 0.114 | 0.01 | 0.258 | 0.03 | 0.047 | 0.022 |
| *Polititapes aureus* | 246150 | 0.024 | 0.063 | 0.002 | 0.331 | 0.045 | 0.052 | 0.105 |
| *Ruditapes decussatus* | 231749 | 0.107 | 0.002 | 0.053 | 0.108 | 0.011 | 0.007 | 0.031 |
| *Venus nux* | 141935 | 0.163 | 0.734 | 0.044 | 0.117 | 0.003 | 0.022 | 0.33 |
| *Venus verrucosa* | 141936 | 0.051 | 0.009 | 0.023 | 0.303 | 0.054 | 0.014 | 0.069 |
| *Patella caerulea* | 140677 | 0.157 | 0.022 | 0.018 | 0.246 | 0.048 | 0.043 | 0.185 |
| *Patella rustica* | 140683 | 0.225 | 0.1 | 0.032 | 0.032 | 0.071 | 0.131 | 0.235 |
| *Patella ulyssiponensis* | 140684 | 0.013 | 0.001 | 0.088 | 0.105 | 0.005 | 0.06 | 0.065 |
| *Elysia timida* | 139684 | 0.095 | 0.011 | 0.023 | 0.154 | 0.1 | 0.082 | 0.155 |
| *Thuridilla hopei* | 139687 | 0.145 | 0.04 | 0.049 | 0.026 | 0.032 | 0.09 | 0.04 |
| *Aplysia fasciata* | 138755 | 0.038 | 0.085 | 0.141 | 0.247 | 0.016 | 0.046 | 0.036 |
| *Aplysia punctata* | 138758 | 0.132 | 0.002 | 0.051 | 0.013 | 0.026 | 0.007 | 0.083 |
| *Bittium reticulatum* | 139054 | 0.059 | 0.008 | 0.094 | 0.058 | 0.001 | 0.007 | 0.065 |
| *Cerithium vulgatum* | 139066 | 0.038 | 0.004 | 0.032 | 0.129 | 0.025 | 0.077 | 0.124 |
| *Turritellinella tricarinata* | 1381415 | 0.074 | 0.027 | 0.277 | 0.022 | 0.001 | 0.025 | 0.15 |
| *Scaphander lignarius* | 139488 | 0.062 | 0.117 | 0.006 | 0.436 | 0 | 0.175 | 0.71 |
| *Haliotis tuberculata* | 140059 | 0.062 | 0.022 | 0.018 | 0.141 | 0.05 | 0.024 | 0.25 |
| *Aporrhais pespelecani* | 138760 | 0.056 | 0.032 | 0.13 | 0.531 | 0.001 | 0.026 | 0.048 |
| *Aporrhais serresiana* | 138761 | 0.091 | 0.405 | 0.017 | 0.427 | 0 | 0.015 | 0.212 |
| *Calyptraea chinensis* | 138961 | 0.116 | 0.042 | 0.098 | 0.044 | 0.002 | 0.026 | 0.028 |
| *Luria lurida* | 139499 | 0.165 | 0.126 | 0.051 | 0.113 | 0.072 | 0.263 | 0.213 |
| *Echinolittorina punctata* | 345757 | 0.042 | 0.055 | 0.121 | 0.324 | 0.046 | 0.116 | 0.167 |
| *Melarhaphe neritoides* | 140266 | 0.071 | 0.006 | 0.056 | 0.114 | 0.034 | 0.036 | 0.081 |
| *Euspira fusca* | 140529 | 0.067 | 0.072 | 0.046 | 0.422 | 0.001 | 0.01 | 0.409 |
| *Naticarius stercusmuscarum* | 720574 | 0.035 | 0.03 | 0.006 | 0.582 | 0.001 | 0.047 | 0.033 |
| *Neverita josephinia* | 140549 | 0.018 | 0.174 | 0.052 | 0.557 | 0.003 | 0.128 | 0.069 |
| *Ranella olearium* | 141115 | 0.005 | 0.52 | 0.035 | 0.099 | 0.013 | 0.008 | 0.035 |
| *Conomurex persicus* | 565371 | 0.496 | 0.003 | 0.063 | 0.03 | 0.03 | 0.038 | 0.011 |
| *Tonna galea* | 141687 | 0.041 | 0.005 | 0.013 | 0.41 | 0.076 | 0.006 | 0.116 |
| *Thylacodes arenarius* | 709464 | 0.024 | 0.063 | 0.098 | 0.173 | 0.003 | 0.195 | 0.068 |
| *Xenophora crispa* | 743862 | 0.045 | 0.551 | 0.031 | 0.135 | 0.006 | 0.013 | 0.13 |
| *Columbella rustica* | 139196 | 0.064 | 0 | 0.079 | 0.067 | 0.08 | 0.114 | 0.119 |
| *Conus ventricosus* | 428401 | 0.013 | 0.002 | 0.11 | 0.235 | 0.063 | 0.061 | 0.04 |
| *Bolinus brandaris* | 140389 | 0.019 | 0.028 | 0.022 | 0.325 | 0.006 | 0.022 | 0.246 |
| *Hexaplex trunculus* | 140396 | 0.016 | 0.017 | 0.005 | 0.227 | 0.043 | 0.04 | 0.107 |
| *Ocenebra erinaceus* | 140405 | 0.225 | 0.046 | 0.091 | 0.138 | 0.008 | 0.035 | 0.074 |
| *Stramonita haemastoma* | 140417 | 0.101 | 0.019 | 0.032 | 0.183 | 0.057 | 0.062 | 0.05 |
| *Tritia incrassata* | 876825 | 0.042 | 0.042 | 0.051 | 0.038 | 0.017 | 0.024 | 0.148 |
| *Tritia mutabilis* | 876840 | 0.077 | 0.298 | 0.22 | 0.805 | 0.017 | 0.069 | 0.216 |
| *Pisania striata* | 138924 | 0.002 | 0 | 0.037 | 0.165 | 0.051 | 0.12 | 0.11 |
| *Euthria cornea* | 181057 | 0.029 | 0.1 | 0.033 | 0.318 | 0.009 | 0.037 | 0.213 |
| *Felimare orsinii* | 597533 | 0.114 | 0.632 | 0.026 | 0.482 | 0 | 0.245 | 0.078 |
| *Felimare picta* | 597522 | 0.145 | 0.013 | 0.019 | 0.169 | 0.051 | 0.152 | 0.042 |
| *Felimare tricolor* | 597530 | 0.037 | 0.087 | 0.1 | 0.425 | 0.001 | 0.272 | 0.169 |
| *Felimare villafranca* | 597536 | 0.374 | 0.004 | 0.034 | 0.029 | 0.001 | 0.087 | 0.012 |
| *Peltodoris atromaculata* | 509315 | 0.034 | 0.095 | 0.043 | 0.172 | 0.022 | 0.206 | 0.068 |
| *Cratena peregrina* | 146862 | 0.051 | 0.02 | 0.125 | 0.007 | 0.003 | 0.062 | 0.069 |
| *Edmundsella pedata* | 1047602 | 0.064 | 0.006 | 0.11 | 0.029 | 0.002 | 0.174 | 0.067 |
| *Flabellina affinis* | 139988 | 0.037 | 0.005 | 0.062 | 0.085 | 0.002 | 0.026 | 0.104 |
| *Paraflabellina ischitana* | 1048137 | 0.084 | 0.22 | 0.038 | 0.053 | 0.004 | 0.175 | 0.041 |
| *Tethys fimbria* | 141643 | 0.049 | 0.086 | 0.089 | 0.112 | 0.01 | 0.021 | 0.328 |
| *Pleurobranchaea meckeli* | 140818 | 0.107 | 0.036 | 0.061 | 0.23 | 0.001 | 0.046 | 0.191 |
| *Calliostoma granulatum* | 141753 | 0.212 | 0.042 | 0.115 | 0.068 | 0.005 | 0.001 | 0.189 |
| *Phorcus articulatus* | 689174 | 0.032 | 0.01 | 0.029 | 0.189 | 0.02 | 0.094 | 0.175 |
| *Phorcus turbinatus* | 689179 | 0.039 | 0.019 | 0.03 | 0.212 | 0.028 | 0.051 | 0.082 |
| *Bolma rugosa* | 141855 | 0.096 | 0.002 | 0.087 | 0.043 | 0.015 | 0.023 | 0.043 |
| *Umbraculum umbraculum* | 141879 | 0.161 | 0.151 | 0.034 | 0.109 | 0.016 | 0.23 | 0.038 |
| *Agelas oroides* | 132454 | 0.042 | 0.033 | 0.163 | 0.044 | 0.006 | 0.034 | 0.098 |
| *Axinella damicornis* | 132472 | 0.086 | 0.08 | 0.032 | 0.233 | 0.013 | 0.109 | 0.086 |
| *Axinella polypoides* | 132487 | 0.071 | 0.176 | 0.072 | 0.167 | 0.007 | 0.204 | 0.153 |
| *Acanthella acuta* | 132455 | 0.057 | 0.129 | 0.03 | 0.333 | 0 | 0.125 | 0.349 |
| *Chondrilla nucula* | 134110 | 0.067 | 0.055 | 0.035 | 0.272 | 0.016 | 0.125 | 0.109 |
| *Chondrosia reniformis* | 134112 | 0.051 | 0.012 | 0.095 | 0.135 | 0.011 | 0.063 | 0.104 |
| *Cliona celata* | 134121 | 0.1 | 0.023 | 0.06 | 0.06 | 0.02 | 0.02 | 0.031 |
| *Cliona viridis* | 134146 | 0.067 | 0.193 | 0.288 | 0.032 | 0.007 | 0.122 | 0.232 |
| *Spirastrella cunctatrix* | 134235 | 0.034 | 0.025 | 0.075 | 0.005 | 0.017 | 0.08 | 0.121 |
| *Dysidea fragilis* | 132324 | 0.015 | 0.013 | 0.032 | 0.306 | 0.015 | 0.064 | 0.077 |
| *Pleraplysilla spinifera* | 132317 | 0.091 | 0.452 | 0.121 | 0.393 | 0.001 | 0.242 | 0.237 |
| *Ircinia oros* | 132356 | 0.126 | 0.054 | 0.038 | 0.048 | 0.009 | 0.042 | 0.098 |
| *Ircinia variabilis* | 132362 | 0.092 | 0.005 | 0.109 | 0.01 | 0.023 | 0.032 | 0.061 |
| *Sarcotragus fasciculatus* | 165081 | 0.213 | 0.048 | 0.157 | 0.051 | 0.055 | 0.075 | 0.028 |
| *Sarcotragus spinosulus* | 165086 | 0.058 | 0.019 | 0.083 | 0.192 | 0.088 | 0.1 | 0.026 |
| *Spongia (Spongia) officinalis* | 165220 | 0.025 | 0.067 | 0.098 | 0.17 | 0.007 | 0.137 | 0.104 |
| *Scalarispongia scalaris* | 165376 | 0.042 | 0.085 | 0.028 | 0.227 | 0.005 | 0.094 | 0.204 |
| *Petrosia (Petrosia) ficiformis* | 166837 | 0.031 | 0.118 | 0.028 | 0.227 | 0.021 | 0.048 | 0.052 |
| *Crambe crambe* | 133445 | 0.041 | 0.109 | 0.032 | 0.188 | 0.006 | 0.098 | 0.091 |
| *Hemimycale columella* | 133543 | 0.075 | 0.11 | 0.044 | 0.227 | 0.02 | 0.066 | 0.02 |
| *Phorbas tenacior* | 133693 | 0.039 | 0.069 | 0.039 | 0.172 | 0.004 | 0.048 | 0.05 |
| *Suberites domuncula* | 134282 | 0.336 | 0.111 | 0.017 | 0.056 | 0.018 | 0.027 | 0.416 |
| *Tethya aurantium* | 134311 | 0.068 | 0.006 | 0.111 | 0.063 | 0.045 | 0.088 | 0.209 |
| *Aplysina aerophoba* | 133911 | 0.018 | 0.083 | 0.036 | 0.098 | 0.031 | 0.031 | 0.362 |
| *Aplysina cavernicola* | 133913 | 0.145 | 0.05 | 0.235 | 0.045 | 0.006 | 0.198 | 0.055 |

**Table S4** – Ecological Indicator Value (EIV) for the modeled species.

| **id** | **Name** | **AphiaID** | **EIV-T_median_** | **EIV-O2_median_** | **EIV-SAL_median_** | **EIV-T_min_** | **EIV-CHL_median_** | **EIV-Vm_median_** |
| --- | --- | --- | --- | --- | --- | --- | --- | --- |
| 1 | *Heteromastus filiformis* | 129884 | 15.47 | 246.04 | 37.96 | 10.69 | 0.25 | 0.01 |
| 2 | *Notomastus latericeus* | 129898 | 15.27 | 246.44 | 37.98 | 10.56 | 0.27 | 0.01 |
| 3 | *Myriochele heeri* | 130542 | 14.9 | 246.7 | 37.99 | 10.4 | 0.27 | 0.01 |
| 4 | *Owenia fusiformis* | 130544 | 15.77 | 244.81 | 37.93 | 11.94 | 0.21 | 0.01 |
| 5 | *Hermodice carunculata* | 129831 | 15.81 | 235.05 | 38.84 | 13.91 | 0.1 | 0.01 |
| 6 | *Bonellia viridis* | 110363 | 14.64 | 240.47 | 38.14 | 12.8 | 0.15 | 0.01 |
| 7 | *Eunice vittata* | 130067 | 15.76 | 243 | 37.97 | 12.22 | 0.19 | 0.01 |
| 8 | *Lumbrineris latreilli* | 130248 | 15.64 | 245.4 | 37.97 | 11.42 | 0.21 | 0.01 |
| 9 | *Drilonereis filum* | 129856 | 15.34 | 245.76 | 37.99 | 11.21 | 0.21 | 0.01 |
| 10 | *Aponuphis bilineata* | 130452 | 15.6 | 244.69 | 37.95 | 12.25 | 0.21 | 0.01 |
| 11 | *Hyalinoecia tubicola* | 130464 | 14.22 | 236.58 | 38.11 | 13.02 | 0.14 | 0.01 |
| 12 | *Aphrodita aculeata* | 129840 | 14.5 | 240.39 | 38.12 | 12.94 | 0.16 | 0.01 |
| 13 | *Laetmonice hystrix* | 129845 | 14.87 | 239.61 | 38.05 | 12.98 | 0.17 | 0.01 |
| 14 | *Glycera alba* | 130116 | 15.34 | 246.11 | 37.97 | 10.71 | 0.25 | 0.01 |
| 15 | *Glycera tridactyla* | 130130 | 16.7 | 242.86 | 37.94 | 12.02 | 0.2 | 0.01 |
| 16 | *Glycera unicornis* | 130131 | 15.7 | 245.49 | 37.95 | 11.41 | 0.22 | 0.01 |
| 17 | *Goniada maculata* | 130140 | 15.26 | 246.11 | 37.98 | 10.61 | 0.26 | 0.01 |
| 18 | *Nephtys hombergii* | 130359 | 16.11 | 243.89 | 37.92 | 12.04 | 0.21 | 0.01 |
| 19 | *Sigalion mathildae* | 131072 | 16.09 | 243.81 | 37.9 | 11.43 | 0.21 | 0.01 |
| 20 | *Sabella pavonina* | 130967 | 14.98 | 242 | 38.09 | 12.76 | 0.17 | 0.01 |
| 21 | *Sabella spallanzanii* | 130969 | 15.25 | 240.87 | 38.1 | 12.86 | 0.15 | 0.01 |
| 22 | *Protula intestinum* | 131032 | 14.79 | 239.26 | 38.12 | 12.88 | 0.15 | 0.01 |
| 23 | *Protula tubularia* | 131035 | 15.21 | 240.42 | 38.15 | 12.9 | 0.13 | 0.01 |
| 24 | *Serpula vermicularis* | 131051 | 15.19 | 241.24 | 38.1 | 12.79 | 0.16 | 0.01 |
| 25 | *Laonice cirrata* | 131128 | 15.22 | 245.76 | 38 | 11.03 | 0.24 | 0.01 |
| 26 | *Prionospio steenstrupi* | 131164 | 15.44 | 242.79 | 37.97 | 11.72 | 0.21 | 0.01 |
| 27 | *Ampharete grubei* | 152272 | 14.69 | 246.28 | 38.01 | 10.51 | 0.26 | 0.01 |
| 28 | *Melinna palmata* | 129808 | 15.64 | 245.52 | 37.97 | 11.15 | 0.21 | 0.01 |
| 29 | *Sternaspis scutata* | 131242 | 14.65 | 244.09 | 38.04 | 10.81 | 0.22 | 0.01 |
| 30 | *Eupolymnia nebulosa* | 131489 | 14.43 | 240.63 | 38.12 | 12.8 | 0.16 | 0.01 |
| 31 | *Ampelisca sarsi* | 101923 | 14.92 | 248.07 | 37.97 | 10.29 | 0.29 | 0.01 |
| 32 | *Ampelisca typica* | 101933 | 14.9 | 246.39 | 38 | 10.53 | 0.27 | 0.01 |
| 33 | *Alpheus glaber* | 107477 | 13.87 | 225.96 | 38.41 | 12.96 | 0.03 | 0.01 |
| 34 | *Athanas nitescens* | 107486 | 15.68 | 241.95 | 37.95 | 12.72 | 0.19 | 0.01 |
| 35 | *Calocaris macandreae* | 107726 | 13.2 | 214.34 | 38.52 | 12.98 | 0 | 0.01 |
| 36 | *Calappa granulata* | 107268 | 14.83 | 234.54 | 38.5 | 13.29 | 0.11 | 0.01 |
| 37 | *Procambarus clarkii* | 465540 | 16.9 | 242.51 | 37.91 | 11.88 | 0.21 | 0.01 |
| 38 | *Carcinus aestuarii* | 107380 | 16.26 | 246.29 | 37.87 | 11.41 | 0.22 | 0.01 |
| 39 | *Aegaeon cataphractus* | 107548 | 14.79 | 239.24 | 38.12 | 12.95 | 0.16 | 0.01 |
| 40 | *Aegaeon lacazei* | 107549 | 13.79 | 219.34 | 38.52 | 13.05 | 0 | 0.01 |
| 41 | *Philocheras echinulatus* | 107558 | 13.25 | 214.44 | 38.51 | 13.05 | 0 | 0.01 |
| 42 | *Pontophilus spinosus* | 107564 | 13.67 | 219.7 | 38.53 | 13.02 | 0 | 0.01 |
| 43 | *Calcinus tubularis* | 107194 | 15.25 | 240.13 | 38.09 | 12.95 | 0.16 | 0.01 |
| 44 | *Clibanarius erythropus* | 107196 | 15.88 | 241.49 | 38.09 | 12.86 | 0.14 | 0.01 |
| 45 | *Dardanus arrosor* | 107197 | 14.21 | 230.1 | 38.22 | 13.04 | 0.09 | 0.01 |
| 46 | *Dardanus calidus* | 107198 | 15.43 | 241.04 | 38.09 | 12.97 | 0.16 | 0.01 |
| 47 | *Diogenes pugilator* | 107199 | 16.08 | 242.48 | 37.91 | 12.37 | 0.19 | 0.01 |
| 48 | *Paguristes eremita* | 107200 | 15.51 | 241.9 | 37.98 | 13 | 0.19 | 0.01 |
| 49 | *Medorippe lanata* | 107288 | 14.69 | 241.02 | 38.17 | 12.59 | 0.15 | 0.01 |
| 50 | *Dromia personata* | 107258 | 14.81 | 240.92 | 38.09 | 12.91 | 0.17 | 0.01 |
| 51 | *Pisa armata* | 107353 | 14.8 | 237.99 | 37.99 | 13.09 | 0.19 | 0.01 |
| 52 | *Eriphia verrucosa* | 107409 | 16.12 | 241.24 | 38.08 | 12.83 | 0.14 | 0.01 |
| 53 | *Ethusa mascarone* | 107283 | 15.5 | 240.18 | 37.94 | 13 | 0.2 | 0.01 |
| 54 | *Galathea intermedia* | 107150 | 14.66 | 238.95 | 38.06 | 12.99 | 0.19 | 0.01 |
| 55 | *Galathea strigosa* | 107155 | 14.62 | 242.93 | 38.11 | 12.52 | 0.17 | 0.01 |
| 56 | *Geryon longipes* | 107373 | 13.73 | 212.41 | 38.67 | 13.39 | 0 | 0 |
| 57 | *Goneplax rhomboides* | 107292 | 14.13 | 231.06 | 38.42 | 12.95 | 0.04 | 0.01 |
| 58 | *Pachygrapsus marmoratus* | 107455 | 16.14 | 241.38 | 38.08 | 12.87 | 0.14 | 0.01 |
| 59 | *Homola barbata* | 107262 | 14.7 | 227.04 | 38.68 | 13.69 | 0.04 | 0.01 |
| 60 | *Paromola cuvieri* | 107264 | 13.8 | 212.66 | 38.68 | 13.49 | 0 | 0 |
| 61 | *Inachus communissimus* | 107326 | 15.26 | 239.35 | 38.1 | 13.08 | 0.17 | 0.01 |
| 62 | *Inachus dorsettensis* | 107327 | 14.27 | 237.3 | 38.12 | 12.98 | 0.17 | 0.01 |
| 63 | *Inachus phalangium* | 107333 | 14.79 | 241.78 | 38.09 | 12.78 | 0.19 | 0.01 |
| 64 | *Inachus thoracicus* | 107334 | 14.87 | 238.79 | 37.88 | 13.09 | 0.21 | 0.01 |
| 65 | *Macropodia linaresi* | 107341 | 14.69 | 237.75 | 37.88 | 13.03 | 0.2 | 0.01 |
| 66 | *Macropodia longirostris* | 107343 | 14.68 | 243.38 | 38.59 | 12.68 | 0.1 | 0.01 |
| 67 | *Macropodia rostrata* | 107345 | 15.16 | 240.04 | 37.95 | 13.03 | 0.19 | 0.01 |
| 68 | *Macropodia tenuirostris* | 107346 | 13.96 | 231.34 | 38.22 | 12.99 | 0.09 | 0.01 |
| 69 | *Latreillia elegans* | 107265 | 14.61 | 227.24 | 38.63 | 13.69 | 0.03 | 0.01 |
| 70 | *Lysmata seticaudata* | 107528 | 14.62 | 242.19 | 38.11 | 12.62 | 0.16 | 0.01 |
| 71 | *Maja crispata* | 107348 | 15.72 | 241.36 | 38.09 | 12.86 | 0.15 | 0.01 |
| 72 | *Maja squinado* | 107350 | 14.54 | 243.18 | 38.27 | 12.7 | 0.14 | 0.01 |
| 73 | *Munida intermedia* | 107157 | 13.72 | 214.64 | 38.59 | 13.15 | 0 | 0 |
| 74 | *Munida tenuimana* | 107166 | 13.18 | 216.21 | 38.52 | 12.96 | 0 | 0.01 |
| 75 | *Iridonida speciosa* | 1606714 | 13.73 | 219.68 | 38.52 | 13.09 | 0 | 0.01 |
| 76 | *Homarus gammarus* | 107253 | 15.07 | 245.55 | 38.08 | 11.63 | 0.19 | 0.01 |
| 77 | *Pagurus alatus* | 107230 | 13.78 | 214.95 | 38.53 | 13.04 | 0 | 0.01 |
| 78 | *Pagurus anachoretus* | 107231 | 15.42 | 241.62 | 38.09 | 12.87 | 0.16 | 0.01 |
| 79 | *Pagurus cuanensis* | 107235 | 14.67 | 240.13 | 38.09 | 12.84 | 0.19 | 0.01 |
| 80 | *Pagurus excavatus* | 107236 | 14 | 234.86 | 38.18 | 12.93 | 0.12 | 0.01 |
| 81 | *Pagurus prideaux* | 107239 | 14.46 | 236.72 | 38.09 | 13.03 | 0.16 | 0.01 |
| 82 | *Palaemon elegans* | 107614 | 15.9 | 241.96 | 38.07 | 12.84 | 0.14 | 0.01 |
| 83 | *Palaemon serratus* | 107616 | 15.76 | 242.07 | 37.97 | 12.74 | 0.19 | 0.01 |
| 84 | *Palinurus elephas* | 107703 | 14.39 | 239.16 | 38.15 | 12.86 | 0.14 | 0.01 |
| 85 | *Chlorotocus crassicornis* | 107642 | 13.96 | 218.99 | 38.66 | 13.28 | 0 | 0.01 |
| 86 | *Plesionika acanthonotus* | 107654 | 13.73 | 212.11 | 38.66 | 13.39 | 0 | 0 |
| 87 | *Plesionika antigai* | 107655 | 13.92 | 214.63 | 38.68 | 13.51 | 0 | 0 |
| 88 | *Plesionika edwardsii* | 107656 | 13.87 | 212.64 | 38.68 | 13.51 | 0 | 0 |
| 89 | *Plesionika gigliolii* | 107659 | 13.81 | 212.52 | 38.67 | 13.47 | 0 | 0 |
| 90 | *Plesionika heterocarpus* | 107660 | 13.88 | 217.18 | 38.67 | 13.27 | 0 | 0.01 |
| 91 | *Plesionika martia* | 107661 | 13.83 | 212.67 | 38.69 | 13.5 | 0 | 0.01 |
| 92 | *Plesionika narval* | 107662 | 13.36 | 219.75 | 38.5 | 13.03 | 0 | 0.01 |
| 93 | *Spinolambrus macrochelos* | 442337 | 14.22 | 221.94 | 38.71 | 13.59 | 0 | 0.01 |
| 94 | *Penaeus kerathurus* | 246388 | 15.88 | 248.54 | 37.88 | 10.21 | 0.26 | 0.01 |
| 95 | *Pilumnus hirtellus* | 107418 | 15.82 | 241.63 | 37.92 | 12.98 | 0.19 | 0.01 |
| 96 | *Pilumnus spinifer* | 107420 | 14.86 | 238.16 | 38 | 13.09 | 0.2 | 0.01 |
| 97 | *Pilumnus villosissimus* | 107421 | 15.61 | 240.46 | 37.9 | 13.03 | 0.2 | 0.01 |
| 98 | *Bathynectes maravigna* | 107377 | 13.92 | 212.77 | 38.77 | 13.54 | 0 | 0.01 |
| 99 | *Polybius vernalis* | 1750288 | 16.42 | 244.6 | 37.87 | 11.9 | 0.2 | 0.01 |
| 100 | *Polybius depurator* | 1750291 | 14.37 | 239.32 | 38.36 | 12.89 | 0.12 | 0.01 |
| 101 | *Macropipus tuberculatus* | 107397 | 13.91 | 219.01 | 38.53 | 13.11 | 0 | 0.01 |
| 102 | *Polycheles typhlops* | 107696 | 13.83 | 212.63 | 38.69 | 13.49 | 0 | 0.01 |
| 103 | *Pisidia longicornis* | 107188 | 15.79 | 241.3 | 37.87 | 12.88 | 0.22 | 0.01 |
| 104 | *Processa canaliculata* | 107682 | 13.35 | 215.71 | 38.53 | 13.05 | 0 | 0.01 |
| 105 | *Processa nouveli* | 107689 | 13.22 | 215.82 | 38.51 | 13 | 0 | 0.01 |
| 106 | *Scyllarides latus* | 107708 | 15 | 238.25 | 38.15 | 12.95 | 0.14 | 0.01 |
| 107 | *Scyllarus arctus* | 107709 | 14.96 | 242.33 | 38.09 | 12.76 | 0.19 | 0.01 |
| 108 | *Solenocera membranacea* | 107120 | 13.9 | 218.45 | 38.54 | 13.06 | 0 | 0.01 |
| 109 | *Stenopus spinosus* | 107721 | 14.86 | 241.44 | 38.14 | 12.87 | 0.16 | 0.01 |
| 110 | *Monodaeus couchii* | 241154 | 13.25 | 216.35 | 38.52 | 13.02 | 0 | 0.01 |
| 111 | *Xantho poressa* | 107442 | 15.81 | 243.16 | 38.03 | 12.24 | 0.17 | 0.01 |
| 112 | *Natatolana borealis* | 118859 | 13.2 | 216.9 | 38.52 | 12.96 | 0 | 0.01 |
| 113 | *Rissoides pallidus* | 136136 | 13.49 | 219.68 | 38.51 | 13.05 | 0 | 0.01 |
| 114 | *Squilla mantis* | 136137 | 15.06 | 246.33 | 38.14 | 11.74 | 0.19 | 0.01 |
| 115 | *Apseudopsis latreillii* | 247077 | 15.22 | 247.48 | 37.97 | 10.29 | 0.28 | 0.01 |
| 116 | *Scalpellum scalpellum* | 106204 | 14.18 | 233.54 | 38.21 | 13.11 | 0.1 | 0.01 |
| 117 | *Gryphus vitreus* | 104068 | 13.78 | 213.68 | 38.62 | 13.27 | 0 | 0 |
| 118 | *Adeonella calveti* | 111055 | 14.16 | 237.93 | 38.14 | 12.87 | 0.16 | 0.01 |
| 119 | *Reptadeonella violacea* | 111061 | 14.35 | 240.84 | 38.12 | 12.79 | 0.16 | 0.01 |
| 120 | *Pentapora fascialis* | 111082 | 15 | 241.06 | 38.1 | 12.8 | 0.16 | 0.01 |
| 122 | *Electra posidoniae* | 111356 | 15.17 | 240.39 | 38.07 | 12.89 | 0.17 | 0.01 |
| 123 | *Myriapora truncata* | 111435 | 14.88 | 239.87 | 38.1 | 12.9 | 0.15 | 0.01 |
| 124 | *Reteporella grimaldii* | 111453 | 14.58 | 240.76 | 38.11 | 12.81 | 0.16 | 0.01 |
| 125 | *Reteporella mediterranea* | 1041728 | 14.18 | 237.62 | 38.14 | 12.85 | 0.13 | 0.01 |
| 126 | *Schizobrachiella sanguinea* | 111522 | 15.2 | 240.95 | 38.08 | 12.77 | 0.17 | 0.01 |
| 127 | *Smittina cervicornis* | 111551 | 14.31 | 240.16 | 38.13 | 12.87 | 0.16 | 0.01 |
| 128 | *Clavelina lepadiformis* | 103552 | 15.3 | 241.12 | 38.07 | 12.79 | 0.19 | 0.01 |
| 129 | *Diazona violacea* | 103733 | 14.38 | 237.57 | 38.11 | 12.96 | 0.17 | 0.01 |
| 130 | *Aplidium conicum* | 103641 | 15.66 | 241.59 | 37.88 | 12.93 | 0.2 | 0.01 |
| 131 | *Aplidium elegans* | 103643 | 15.09 | 242.68 | 37.89 | 12.91 | 0.21 | 0.01 |
| 132 | *Ascidia mentula* | 103710 | 14.76 | 239 | 38.11 | 13.01 | 0.15 | 0.01 |
| 133 | *Ascidia virginea* | 103717 | 14.66 | 240.44 | 38.38 | 13.1 | 0.13 | 0.01 |
| 134 | *Ascidiella aspersa* | 103718 | 14.61 | 236.52 | 38.17 | 13.04 | 0.14 | 0.01 |
| 135 | *Ascidiella scabra* | 103719 | 14.23 | 236.1 | 38.15 | 12.98 | 0.17 | 0.01 |
| 136 | *Phallusia mammillata* | 103724 | 14.87 | 243.22 | 38.09 | 12.66 | 0.17 | 0.01 |
| 137 | *Ciona intestinalis* | 103732 | 14.71 | 240.55 | 38.05 | 12.97 | 0.2 | 0.01 |
| 138 | *Molgula appendiculata* | 103771 | 14.77 | 238.75 | 37.88 | 13.07 | 0.22 | 0.01 |
| 139 | *Halocynthia papillosa* | 103827 | 15.2 | 241.33 | 38.09 | 12.9 | 0.16 | 0.01 |
| 140 | *Microcosmus sabatieri* | 103844 | 14.79 | 239.93 | 38.01 | 12.87 | 0.19 | 0.01 |
| 141 | *Microcosmus vulgaris* | 103846 | 14.47 | 238.51 | 38.1 | 13.02 | 0.15 | 0.01 |
| 142 | *Botryllus schlosseri* | 103862 | 15.27 | 242.18 | 38.01 | 12.83 | 0.2 | 0.01 |
| 143 | *Actinia mediterranea* | 854459 | 15.58 | 241.65 | 38.1 | 12.87 | 0.14 | 0.01 |
| 144 | *Anemonia viridis* | 100808 | 15.56 | 241.78 | 38.08 | 12.86 | 0.14 | 0.01 |
| 145 | *Cribrinopsis crassa* | 100823 | 14.25 | 240.97 | 38.12 | 12.76 | 0.17 | 0.01 |
| 146 | *Aiptasia mutabilis* | 100859 | 15.3 | 241.86 | 38.07 | 12.75 | 0.16 | 0.01 |
| 147 | *Actinauge richardi* | 100930 | 14.07 | 223.39 | 38.72 | 13.46 | 0 | 0.01 |
| 148 | *Calliactis palliata* | 1658483 | 14.75 | 238.91 | 38.01 | 13.01 | 0.17 | 0.01 |
| 149 | *Calliactis parasitica* | 100946 | 14.57 | 237.01 | 38.15 | 13.05 | 0.13 | 0.01 |
| 150 | *Cereus pedunculatus* | 100987 | 14.9 | 241.58 | 38.1 | 12.37 | 0.17 | 0.01 |
| 151 | *Cerianthus membranaceus* | 101011 | 15.64 | 240.05 | 37.99 | 12.9 | 0.16 | 0.01 |
| 152 | *Cerianthus membranacea* | 856035 | 14.18 | 240.74 | 38.13 | 12.78 | 0.17 | 0.01 |
| 155 | *Cladocora caespitosa* | 135146 | 14.77 | 241.5 | 38.12 | 12.79 | 0.14 | 0.01 |
| 156 | *Astroides calycularis* | 135178 | 14.45 | 231.09 | 38.22 | 13.46 | 0.08 | 0.01 |
| 158 | *Leptopsammia pruvoti* | 135193 | 14.49 | 240.61 | 38.15 | 12.83 | 0.13 | 0.01 |
| 159 | *Oculina patagonica* | 135210 | 16.17 | 239.77 | 37.98 | 13.16 | 0.17 | 0.01 |
| 160 | *Parazoanthus axinellae* | 101055 | 14.88 | 242.13 | 38.09 | 12.76 | 0.14 | 0.01 |
| 161 | *Lytocarpia myriophyllum* | 117302 | 14.37 | 234.09 | 38.55 | 13.47 | 0.06 | 0.01 |
| 162 | *Nemertesia antennina* | 117809 | 14.3 | 238.01 | 38.26 | 13.16 | 0.11 | 0.01 |
| 163 | *Nemertesia ramosa* | 117815 | 14.11 | 233.61 | 38.2 | 13.17 | 0.1 | 0.01 |
| 164 | *Paramuricea clavata* | 125387 | 14.45 | 240.53 | 38.15 | 12.84 | 0.13 | 0.01 |
| 165 | *Alcyonium acaule* | 125331 | 14.6 | 240.97 | 38.1 | 12.89 | 0.19 | 0.01 |
| 166 | *Alcyonium coralloides* | 125332 | 14.43 | 240.77 | 38.11 | 12.81 | 0.19 | 0.01 |
| 167 | *Alcyonium palmatum* | 125334 | 14.28 | 235.89 | 38.3 | 12.99 | 0.1 | 0.01 |
| 168 | *Eunicella cavolini* | 125361 | 14.59 | 241.63 | 38.14 | 12.83 | 0.13 | 0.01 |
| 169 | *Eunicella singularis* | 125365 | 14.92 | 241.92 | 38.08 | 12.76 | 0.16 | 0.01 |
| 170 | *Eunicella verrucosa* | 125366 | 14.47 | 239.22 | 38.12 | 12.85 | 0.15 | 0.01 |
| 171 | *Leptogorgia sarmentosa* | 125369 | 14.6 | 238.54 | 38.12 | 12.91 | 0.17 | 0.01 |
| 172 | *Corallium rubrum* | 125416 | 14.18 | 235.52 | 38.17 | 12.83 | 0.12 | 0.01 |
| 173 | *Funiculina quadrangularis* | 128506 | 14 | 221.06 | 38.5 | 13.1 | 0.01 | 0.01 |
| 174 | *Isidella elongata* | 125373 | 14 | 212.97 | 38.77 | 13.69 | 0 | 0.01 |
| 175 | *Pennatula phosphorea* | 128517 | 14.45 | 236.61 | 38.19 | 13.06 | 0.12 | 0.01 |
| 176 | *Pennatula rubra* | 128519 | 14.42 | 235.63 | 38.18 | 13.1 | 0.12 | 0.01 |
| 177 | *Pteroeides griseum* | 181504 | 14.47 | 237.6 | 38.14 | 13.01 | 0.14 | 0.01 |
| 178 | *Veretillum cynomorium* | 128536 | 14.09 | 240.59 | 38.1 | 11.96 | 0.19 | 0.01 |
| 179 | *Coscinasterias tenuispina* | 123795 | 15.3 | 241.5 | 38.14 | 12.87 | 0.14 | 0.01 |
| 180 | *Marthasterias glacialis* | 123803 | 14.78 | 241.55 | 38.19 | 12.9 | 0.14 | 0.01 |
| 181 | *Astropecten aranciacus* | 123856 | 14.71 | 237.93 | 38.09 | 13.03 | 0.15 | 0.01 |
| 182 | *Astropecten bispinosus* | 123859 | 15.67 | 239.6 | 38.09 | 13.23 | 0.13 | 0.01 |
| 183 | *Astropecten irregularis* | 123867 | 14.42 | 238 | 38.1 | 12.67 | 0.19 | 0.01 |
| 184 | *Astropecten jonstoni* | 123868 | 15.98 | 238.66 | 38.1 | 13 | 0.14 | 0.01 |
| 185 | *Astropecten platyacanthus* | 123876 | 15.76 | 242.05 | 38.27 | 12.89 | 0.13 | 0.01 |
| 186 | *Astropecten spinulosus* | 123878 | 15.21 | 239.85 | 38.03 | 12.94 | 0.17 | 0.01 |
| 187 | *Tethyaster subinermis* | 123913 | 14.12 | 220.97 | 38.44 | 13.23 | 0.01 | 0.01 |
| 188 | *Luidia ciliaris* | 123920 | 14.79 | 236.72 | 38.07 | 13.13 | 0.17 | 0.01 |
| 189 | *Luidia sarsii* | 123922 | 13.56 | 217.49 | 38.48 | 13.06 | 0 | 0.01 |
| 191 | *Anseropoda placenta* | 123985 | 14.48 | 236.42 | 38.18 | 13.05 | 0.14 | 0.01 |
| 192 | *Chaetaster longipes* | 124004 | 14.49 | 236.33 | 38.06 | 13.04 | 0.16 | 0.01 |
| 193 | *Peltaster placenta* | 124055 | 14.68 | 233.13 | 38.56 | 13.16 | 0.09 | 0.01 |
| 194 | *Hacelia attenuata* | 124094 | 14.6 | 237.98 | 38.13 | 12.96 | 0.15 | 0.01 |
| 195 | *Ophidiaster ophidianus* | 124101 | 15.16 | 237.73 | 38.24 | 13.24 | 0.12 | 0.01 |
| 196 | *Antedon mediterranea* | 124208 | 14.62 | 236.77 | 38.22 | 13.1 | 0.13 | 0.01 |
| 197 | *Leptometra phalangium* | 124226 | 14.05 | 226.91 | 38.42 | 13.01 | 0.03 | 0.01 |
| 198 | *Arbacia lixula* | 124249 | 15.6 | 240.37 | 38.14 | 12.94 | 0.13 | 0.01 |
| 199 | *Echinus melo* | 124294 | 13.94 | 220.18 | 38.52 | 13.14 | 0 | 0.01 |
| 200 | *Gracilechinus acutus* | 532031 | 13.92 | 222.11 | 38.51 | 13.13 | 0 | 0.01 |
| 201 | *Paracentrotus lividus* | 124316 | 15.65 | 240.85 | 38.09 | 12.85 | 0.14 | 0.01 |
| 202 | *Psammechinus microtuberculatus* | 124318 | 14.88 | 239.95 | 37.93 | 13.14 | 0.18 | 0.01 |
| 203 | *Sphaerechinus granularis* | 124427 | 15.44 | 241.55 | 38.07 | 12.92 | 0.15 | 0.01 |
| 204 | *Cidaris cidaris* | 124257 | 14.2 | 225.12 | 38.5 | 13.23 | 0.01 | 0.01 |
| 205 | *Stylocidaris affinis* | 124268 | 14.58 | 228.82 | 38.54 | 13.51 | 0.03 | 0.01 |
| 206 | *Centrostephanus longispinus* | 124331 | 14.68 | 235.53 | 38.19 | 13.12 | 0.14 | 0.01 |
| 207 | *Diadema setosum* | 213372 | 16.29 | 228.51 | 39.06 | 14.8 | 0.07 | 0.01 |
| 208 | *Brissopsis lyrifera* | 124373 | 13.96 | 240.83 | 38.13 | 11.25 | 0.16 | 0.01 |
| 209 | *Brissus unicolor* | 124380 | 16.14 | 240.46 | 38 | 13 | 0.15 | 0.01 |
| 210 | *Echinocardium cordatum* | 124392 | 16.35 | 241.71 | 37.94 | 12.37 | 0.18 | 0.01 |
| 211 | *Ova canalifera* | 569394 | 15.95 | 247.74 | 38 | 10.29 | 0.22 | 0.01 |
| 212 | *Spatangus purpureus* | 124418 | 14.77 | 239.59 | 38.06 | 12.98 | 0.15 | 0.01 |
| 213 | *Paraleptopentacta elongata* | 1474372 | 14.48 | 242.63 | 38.09 | 11.68 | 0.2 | 0.01 |
| 214 | *Paraleptopentacta tergestina* | 1474379 | 14.49 | 240.78 | 38.09 | 12.49 | 0.18 | 0.01 |
| 215 | *Ocnus planci* | 124647 | 14.52 | 248.68 | 38.13 | 10.28 | 0.19 | 0.01 |
| 219 | *Holothuria forskali* | 1670329 | 14.6 | 239.96 | 38.09 | 12.92 | 0.17 | 0.01 |
| 220 | *Parastichopus regalis* | 149898 | 14.14 | 228.99 | 38.36 | 13.05 | 0.05 | 0.01 |
| 221 | *Amphiura chiajei* | 125073 | 15.56 | 246.21 | 37.95 | 11.1 | 0.23 | 0.01 |
| 222 | *Amphiura filiformis* | 125080 | 14.76 | 246.29 | 38.01 | 10.55 | 0.26 | 0.01 |
| 223 | *Ophiopsila aranea* | 125049 | 15.54 | 241.33 | 37.98 | 12.77 | 0.19 | 0.01 |
| 224 | *Ophiothrix fragilis* | 125131 | 14.61 | 239.23 | 38.11 | 12.94 | 0.15 | 0.01 |
| 225 | *Astrospartus mediterraneus* | 124963 | 14.1 | 231.97 | 38.2 | 12.91 | 0.1 | 0.01 |
| 226 | *Ophioderma longicaudum* | 1420120 | 15.22 | 240.65 | 38.07 | 12.94 | 0.16 | 0.01 |
| 227 | *Ophiura ophiura* | 124929 | 14.7 | 239.61 | 38.08 | 12.94 | 0.17 | 0.01 |
| 228 | *Ensis minor* | 140734 | 16.44 | 242.55 | 37.87 | 12.19 | 0.21 | 0.01 |
| 229 | *Phaxas pellucidus* | 140737 | 15.19 | 248.21 | 37.95 | 10.24 | 0.29 | 0.01 |
| 230 | *Arca noae* | 138788 | 15.85 | 241.04 | 38.07 | 12.83 | 0.15 | 0.01 |
| 231 | *Barbatia barbata* | 138793 | 16.09 | 241.22 | 38.08 | 12.9 | 0.14 | 0.01 |
| 232 | *Glycymeris nummaria* | 504509 | 16.67 | 241.96 | 37.83 | 12.49 | 0.19 | 0.01 |
| 233 | *Acanthocardia aculeata* | 138990 | 15.71 | 241.46 | 37.93 | 12.86 | 0.2 | 0.01 |
| 234 | *Acanthocardia echinata* | 138992 | 14.83 | 241.75 | 38.06 | 12.36 | 0.19 | 0.01 |
| 235 | *Acanthocardia tuberculata* | 381057 | 16.28 | 241.6 | 37.97 | 12.78 | 0.16 | 0.01 |
| 236 | *Cerastoderma glaucum* | 138999 | 16.81 | 243.02 | 37.91 | 12.25 | 0.17 | 0.01 |
| 237 | *Donax semistriatus* | 139601 | 16.87 | 241.95 | 37.87 | 12.05 | 0.2 | 0.01 |
| 238 | *Donax trunculus* | 139602 | 16.79 | 241.65 | 37.88 | 12.56 | 0.17 | 0.01 |
| 239 | *Donax venustus* | 139603 | 16.61 | 241.33 | 37.92 | 12.49 | 0.18 | 0.01 |
| 240 | *Peronaea planata* | 605934 | 17.21 | 241.58 | 37.87 | 12.5 | 0.16 | 0.01 |
| 241 | *Lima lima* | 140233 | 14.98 | 240.96 | 38.11 | 12.87 | 0.15 | 0.01 |
| 242 | *Limaria hians* | 140235 | 15.5 | 241.95 | 38.02 | 12.77 | 0.19 | 0.01 |
| 243 | *Limaria tuberculata* | 140236 | 16.23 | 241.17 | 37.95 | 12.85 | 0.16 | 0.01 |
| 244 | *Loripes orbiculatus* | 875379 | 15.99 | 242.16 | 37.92 | 12.44 | 0.16 | 0.01 |
| 245 | *Lithophaga lithophaga* | 140459 | 15.43 | 243.93 | 38.11 | 12.44 | 0.14 | 0.01 |
| 246 | *Mytilus galloprovincialis* | 140481 | 15.85 | 245.24 | 37.94 | 11.41 | 0.21 | 0.01 |
| 247 | *Nucula sulcata* | 140592 | 14.81 | 243.85 | 38.02 | 11.74 | 0.21 | 0.01 |
| 248 | *Neopycnodonte cochlear* | 140048 | 14.25 | 232.26 | 38.31 | 13.12 | 0.07 | 0.01 |
| 249 | *Pinctada radiata* | 140890 | 16.64 | 236.74 | 38.94 | 13.55 | 0.12 | 0.01 |
| 250 | *Magallana gigas* | 836033 | 16.14 | 249.24 | 37.78 | 9.66 | 0.29 | 0.01 |
| 251 | *Ostrea edulis* | 140658 | 15.29 | 243.47 | 38.1 | 12.36 | 0.17 | 0.01 |
| 252 | *Pinna nobilis* | 140780 | 15.44 | 241.88 | 38.09 | 12.8 | 0.14 | 0.01 |
| 253 | *Pinna rudis* | 140781 | 15.2 | 239.61 | 38.05 | 13.04 | 0.16 | 0.01 |
| 254 | *Pteria hirundo* | 140891 | 14.1 | 235.26 | 38.23 | 12.93 | 0.1 | 0.01 |
| 255 | *Anomia ephippium* | 138748 | 15.71 | 241.69 | 37.95 | 12.87 | 0.17 | 0.01 |
| 256 | *Aequipecten opercularis* | 140687 | 15.46 | 242.72 | 37.87 | 12.94 | 0.25 | 0.01 |
| 257 | *Mimachlamys varia* | 236719 | 15.72 | 242.23 | 37.91 | 12.85 | 0.2 | 0.01 |
| 258 | *Pecten jacobaeus* | 394429 | 15.61 | 243.77 | 37.95 | 12.21 | 0.21 | 0.01 |
| 259 | *Spondylus gaederopus* | 141549 | 15.93 | 239.44 | 38.19 | 12.95 | 0.13 | 0.01 |
| 260 | *Mactra stultorum* | 140299 | 16.82 | 241.95 | 37.9 | 12.41 | 0.18 | 0.01 |
| 261 | *Spisula subtruncata* | 140302 | 16.14 | 243.76 | 37.87 | 12.13 | 0.22 | 0.01 |
| 262 | *Donacilla cornea* | 140350 | 16.79 | 241.74 | 37.92 | 12.53 | 0.17 | 0.01 |
| 263 | *Callista chione* | 141906 | 16.08 | 242.48 | 37.93 | 12.49 | 0.17 | 0.01 |
| 264 | *Chamelea gallina* | 141907 | 16.5 | 241.71 | 37.9 | 12.61 | 0.17 | 0.01 |
| 265 | *Dosinia lupinus* | 141912 | 15.87 | 245.51 | 37.9 | 11.35 | 0.21 | 0.01 |
| 266 | *Polititapes aureus* | 246150 | 16.37 | 242.09 | 37.86 | 12.17 | 0.19 | 0.01 |
| 267 | *Ruditapes decussatus* | 231749 | 16.61 | 241.85 | 37.88 | 12.49 | 0.18 | 0.01 |
| 268 | *Venus nux* | 141935 | 14.23 | 229.21 | 38.14 | 13.12 | 0.15 | 0.01 |
| 269 | *Venus verrucosa* | 141936 | 15.94 | 241.59 | 37.97 | 12.82 | 0.16 | 0.01 |
| 270 | *Patella caerulea* | 140677 | 15.97 | 241.01 | 38.09 | 12.89 | 0.14 | 0.01 |
| 271 | *Patella rustica* | 140683 | 15.74 | 241.22 | 38.01 | 12.85 | 0.15 | 0.01 |
| 272 | *Patella ulyssiponensis* | 140684 | 15.94 | 241.68 | 38.03 | 12.9 | 0.16 | 0.01 |
| 273 | *Elysia timida* | 139684 | 15.26 | 241.12 | 38.12 | 12.94 | 0.16 | 0.01 |
| 274 | *Thuridilla hopei* | 139687 | 15.16 | 242.23 | 38.1 | 12.74 | 0.15 | 0.01 |
| 275 | *Aplysia fasciata* | 138755 | 15.61 | 242.86 | 38.09 | 12.67 | 0.18 | 0.01 |
| 276 | *Aplysia punctata* | 138758 | 15.54 | 241.86 | 37.99 | 12.88 | 0.18 | 0.01 |
| 277 | *Bittium reticulatum* | 139054 | 16.05 | 241.71 | 37.92 | 12.79 | 0.19 | 0.01 |
| 278 | *Cerithium vulgatum* | 139066 | 16.03 | 241.67 | 38.08 | 12.8 | 0.15 | 0.01 |
| 279 | *Turritellinella tricarinata* | 1381415 | 15.34 | 244.39 | 37.99 | 11 | 0.21 | 0.01 |
| 280 | *Scaphander lignarius* | 139488 | 13.91 | 230.91 | 38.31 | 12.92 | 0.05 | 0.01 |
| 281 | *Haliotis tuberculata* | 140059 | 15.73 | 241.77 | 38.1 | 12.8 | 0.14 | 0.01 |
| 282 | *Aporrhais pespelecani* | 138760 | 15.5 | 241.4 | 38.05 | 12.56 | 0.17 | 0.01 |
| 283 | *Aporrhais serresiana* | 138761 | 13.22 | 215.14 | 38.51 | 12.98 | 0 | 0.01 |
| 284 | *Calyptraea chinensis* | 138961 | 15.53 | 243.13 | 37.87 | 12.8 | 0.25 | 0.01 |
| 285 | *Galeodea echinophora* | 139023 | 14.08 | 229.44 | 38.56 | 13.07 | 0.01 | 0.01 |
| 286 | *Galeodea rugosa* | 139024 | 13.83 | 217.48 | 38.52 | 13.02 | 0 | 0.01 |
| 287 | *Luria lurida* | 139499 | 15.02 | 239.78 | 38.16 | 12.87 | 0.12 | 0.01 |
| 288 | *Echinolittorina punctata* | 345757 | 16.26 | 238.89 | 37.95 | 13.28 | 0.16 | 0.01 |
| 289 | *Melarhaphe neritoides* | 140266 | 15.93 | 242.34 | 37.98 | 12.62 | 0.16 | 0.01 |
| 290 | *Euspira fusca* | 140529 | 13.25 | 217.06 | 38.49 | 12.96 | 0 | 0.01 |
| 291 | *Naticarius stercusmuscarum* | 720574 | 15.67 | 240.64 | 38.05 | 13.43 | 0.14 | 0.01 |
| 292 | *Neverita josephinia* | 140549 | 17 | 241.74 | 37.97 | 12.41 | 0.15 | 0.01 |
| 293 | *Ranella olearium* | 141115 | 13.28 | 216.17 | 38.52 | 13.04 | 0 | 0.01 |
| 294 | *Conomurex persicus* | 565371 | 16.95 | 227.89 | 39.06 | 14.86 | 0.08 | 0.01 |
| 295 | *Tonna galea* | 141687 | 15.78 | 238.43 | 38.7 | 13.46 | 0.12 | 0.01 |
| 296 | *Thylacodes arenarius* | 709464 | 14.84 | 241.21 | 38.12 | 12.76 | 0.16 | 0.01 |
| 297 | *Xenophora crispa* | 743862 | 13.44 | 217.34 | 38.32 | 13.1 | 0.01 | 0.01 |
| 298 | *Columbella rustica* | 139196 | 15.97 | 240.39 | 38.09 | 12.98 | 0.13 | 0.01 |
| 299 | *Conus ventricosus* | 428401 | 16.31 | 240.22 | 38.13 | 13.03 | 0.13 | 0.01 |
| 300 | *Bolinus brandaris* | 140389 | 15.55 | 241.7 | 38.02 | 12.64 | 0.18 | 0.01 |
| 301 | *Hexaplex trunculus* | 140396 | 16.05 | 241.96 | 38.07 | 12.75 | 0.15 | 0.01 |
| 302 | *Ocenebra erinaceus* | 140405 | 15.76 | 241.62 | 37.83 | 12.44 | 0.22 | 0.01 |
| 303 | *Stramonita haemastoma* | 140417 | 15.96 | 240.54 | 38.04 | 12.95 | 0.15 | 0.01 |
| 304 | *Tritia incrassata* | 876825 | 15.81 | 242.1 | 37.94 | 12.42 | 0.19 | 0.01 |
| 305 | *Tritia mutabilis* | 876840 | 16.55 | 242.05 | 37.9 | 12.25 | 0.19 | 0.01 |
| 306 | *Pisania striata* | 138924 | 15.73 | 241.03 | 38.35 | 13.05 | 0.13 | 0.01 |
| 307 | *Euthria cornea* | 181057 | 15.61 | 241.6 | 38.09 | 12.94 | 0.17 | 0.01 |
| 308 | *Felimare picta* | 597522 | 15.21 | 241.02 | 38.12 | 12.88 | 0.15 | 0.01 |
| 309 | *Felimare tricolor* | 597530 | 14.68 | 240.39 | 38.11 | 12.83 | 0.16 | 0.01 |
| 310 | *Felimare orsinii* | 597533 | 14.2 | 240.9 | 38.14 | 12.79 | 0.16 | 0.01 |
| 311 | *Felimare villafranca* | 597536 | 15.34 | 242.16 | 37.93 | 12.72 | 0.21 | 0.01 |
| 312 | *Peltodoris atromaculata* | 509315 | 15.04 | 240.94 | 38.12 | 12.84 | 0.14 | 0.01 |
| 313 | *Cratena peregrina* | 146862 | 15.19 | 242 | 38.11 | 12.8 | 0.14 | 0.01 |
| 314 | *Edmundsella pedata* | 1047602 | 14.9 | 242.09 | 38.11 | 12.77 | 0.18 | 0.01 |
| 315 | *Flabellina affinis* | 139988 | 15.22 | 240.61 | 38.12 | 12.91 | 0.14 | 0.01 |
| 316 | *Paraflabellina ischitana* | 1048137 | 14.6 | 241.81 | 38.12 | 12.76 | 0.16 | 0.01 |
| 317 | *Tethys fimbria* | 141643 | 14.71 | 241.05 | 38.12 | 12.81 | 0.17 | 0.01 |
| 318 | *Pleurobranchaea meckeli* | 140818 | 14.88 | 240.94 | 38.11 | 13.03 | 0.17 | 0.01 |
| 319 | *Calliostoma granulatum* | 141753 | 14.27 | 237.05 | 38.14 | 12.9 | 0.16 | 0.01 |
| 320 | *Phorcus articulatus* | 689174 | 15.93 | 242.02 | 38.11 | 12.88 | 0.13 | 0.01 |
| 321 | *Phorcus turbinatus* | 689179 | 15.88 | 240.35 | 38.13 | 12.97 | 0.13 | 0.01 |
| 322 | *Bolma rugosa* | 141855 | 15.55 | 241.05 | 37.97 | 12.95 | 0.19 | 0.01 |
| 323 | *Umbraculum umbraculum* | 141879 | 14.87 | 238.41 | 38.09 | 12.97 | 0.15 | 0.01 |
| 324 | *Rhyssoplax olivacea* | 1392276 | 15.76 | 241.57 | 38.09 | 12.87 | 0.14 | 0.01 |
| 325 | *Clathrina clathrus* | 132275 | 14.44 | 240.65 | 38.12 | 12.84 | 0.15 | 0.01 |
| 326 | *Agelas oroides* | 132454 | 14.91 | 240.82 | 38.16 | 12.87 | 0.14 | 0.01 |
| 327 | *Axinella damicornis* | 132472 | 14.9 | 240.24 | 38.06 | 12.93 | 0.19 | 0.01 |
| 328 | *Axinella polypoides* | 132487 | 14.55 | 240.05 | 38.11 | 12.86 | 0.17 | 0.01 |
| 329 | *Axinella verrucosa* | 132499 | 14.41 | 240.2 | 38.14 | 12.86 | 0.15 | 0.01 |
| 330 | *Acanthella acuta* | 132455 | 14.36 | 241.52 | 38.11 | 12.8 | 0.16 | 0.01 |
| 331 | *Chondrilla nucula* | 134110 | 15.54 | 239.71 | 38.49 | 12.98 | 0.12 | 0.01 |
| 332 | *Chondrosia reniformis* | 134112 | 15.21 | 241.18 | 38.11 | 12.84 | 0.14 | 0.01 |
| 333 | *Cliona celata* | 134121 | 15.44 | 241.53 | 37.99 | 12.87 | 0.18 | 0.01 |
| 334 | *Cliona viridis* | 134146 | 15.04 | 241.01 | 38.09 | 12.9 | 0.18 | 0.01 |
| 335 | *Spirastrella cunctatrix* | 134235 | 14.9 | 241.51 | 38.14 | 12.86 | 0.13 | 0.01 |
| 336 | *Dysidea fragilis* | 132324 | 15.2 | 240.22 | 38.08 | 12.9 | 0.17 | 0.01 |
| 337 | *Pleraplysilla spinifera* | 132317 | 14.23 | 240.82 | 38.13 | 12.78 | 0.17 | 0.01 |
| 338 | *Ircinia oros* | 132356 | 14.86 | 241.95 | 38.08 | 12.76 | 0.17 | 0.01 |
| 339 | *Ircinia variabilis* | 132362 | 15.1 | 241.02 | 38.13 | 12.84 | 0.14 | 0.01 |
| 340 | *Sarcotragus fasciculatus* | 165081 | 15.76 | 240.67 | 38.05 | 12.99 | 0.16 | 0.01 |
| 341 | *Sarcotragus spinosulus* | 165086 | 15.81 | 239.97 | 38.14 | 12.96 | 0.13 | 0.01 |
| 343 | *Scalarispongia scalaris* | 165376 | 14.61 | 241.58 | 38.11 | 12.81 | 0.17 | 0.01 |
| 345 | *Crambe crambe* | 133445 | 15.21 | 240.67 | 38.11 | 12.85 | 0.14 | 0.01 |
| 346 | *Hemimycale columella* | 133543 | 14.99 | 240.87 | 38.09 | 12.85 | 0.16 | 0.01 |
| 347 | *Phorbas tenacior* | 133693 | 14.67 | 241.52 | 38.12 | 12.8 | 0.15 | 0.01 |
| 348 | *Suberites domuncula* | 134282 | 14.78 | 240.01 | 38.07 | 12.98 | 0.16 | 0.01 |
| 349 | *Tethya aurantium* | 134311 | 14.95 | 242.04 | 38.01 | 12.92 | 0.19 | 0.01 |
| 350 | *Thenea muricata* | 134106 | 13.74 | 217.35 | 38.53 | 13.05 | 0 | 0.01 |
| 351 | *Aplysina aerophoba* | 133911 | 15.57 | 243.22 | 38.21 | 12.56 | 0.14 | 0.01 |
| 352 | *Aplysina cavernicola* | 133913 | 14.18 | 240.74 | 38.15 | 12.79 | 0.15 | 0.01 |
| 353 | *Oscarella lobularis* | 133928 | 14.86 | 241.95 | 38.1 | 12.77 | 0.17 | 0.01 |

**Table S5** – Changes in alpha diversity (*Δα*) and beta diversity (*β_ratio_*) averaged over the subbasins Values in parenthesis represent the interquartile range. See Fig 1 in the main text for the spatial distribution of the basins. alb: Alborean Sea; nwm: north-west Mediterranean; swm: south-west Mediterranean; tyr: Tyrrhenian sea; ion: Ionian sea; adr: Adriatic sea; aeg: Aegean sea; lev: Levantine sea

| **Metric** | **Subbasin** | **2040-2060;-RCP4.5** | **2080-2100; RCP4.5** | **2040-2060; RCP8.5** | **2080-2100; RCP8.5** |
| --- | --- | --- | --- | --- | --- |
| *Δα* | adr | 27.8 (6.59; 46.86) | 20.05 (4.43; 48.56) | 27.09 (8.05; 56.5) | 6.48 (-9.54; 23.18) |
| *Δα* | aeg | 8.14 (-0.98; 16.03) | 1.15 (-4.54; 5.76) | 2.19 (-3.04; 6.97) | -9.27 (-18.33; -3.27) |
| *Δα* | alb | 6.84 (-0.12; 13.53) | 8.62 (-0.24; 17.3) | 6.67 (-4.6; 13.87) | 6.84 (-3.76; 15.03) |
| *Δα* | ion | 2.1 (-6.46; 7.57) | -3.59 (-13.82; 2.24) | -1.52 (-11.12; 3.13) | -13.63 (-32.21; -3.08) |
| *Δα* | lev | 5.55 (2.31; 9.07) | 2.15 (-1.52; 7.29) | 1.92 (-1.16; 7.35) | -2.23 (-12.12; 5.64) |
| *Δα* | nwm | -2.34 (-15.58; 4.36) | -4.6 (-18.92; 1.55) | -1.98 (-14.34; 4.6) | -20.68 (-46.21; -2.21) |
| *Δα* | swm | -2.04 (-23.83; 4.44) | -4.05 (-27.35; 3.12) | -3.89 (-23.18; 3.03) | -10.23 (-46.81; 1.75) |
| *Δα* | tyr | 0.53 (-8.61; 7.43) | -1.9 (-16.89; 4.67) | -0.9 (-10.85; 4.77) | -6.66 (-38.68; 0.5) |
| *β_ratio_* | adr | 0.81 (0.56; 0.91) | 0.67 (0.43; 0.86) | 0.78 (0.49; 0.91) | 0.45 (0.22; 0.71) |
| *β_ratio_* | aeg | 0.65 (0.47; 0.8) | 0.38 (0.2; 0.56) | 0.38 (0.22; 0.55) | 0.32 (0.14; 0.54) |
| *β_ratio_* | alb | 0.67 (0.53; 0.82) | 0.69 (0.51; 0.81) | 0.67 (0.51; 0.82) | 0.69 (0.5; 0.81) |
| *β_ratio_* | ion | 0.59 (0.33; 0.81) | 0.48 (0.24; 0.79) | 0.47 (0.23; 0.79) | 0.57 (0.21; 0.84) |
| *β_ratio_* | lev | 0.7 (0.41; 1) | 0.47 (0.2; 1) | 0.5 (0.2; 1) | 0.71 (0.36; 1) |
| *β_ratio_* | nwm | 0.42 (0.2; 0.7) | 0.43 (0.2; 0.68) | 0.39 (0.18; 0.68) | 0.53 (0.31; 0.71) |
| *β_ratio_* | swm | 0.6 (0.32; 0.83) | 0.54 (0.27; 0.8) | 0.53 (0.3; 0.78) | 0.64 (0.32; 0.83) |
| *β_ratio_* | tyr | 0.57 (0.29; 0.8) | 0.5 (0.24; 0.78) | 0.5 (0.25; 0.78) | 0.53 (0.23; 0.83) |


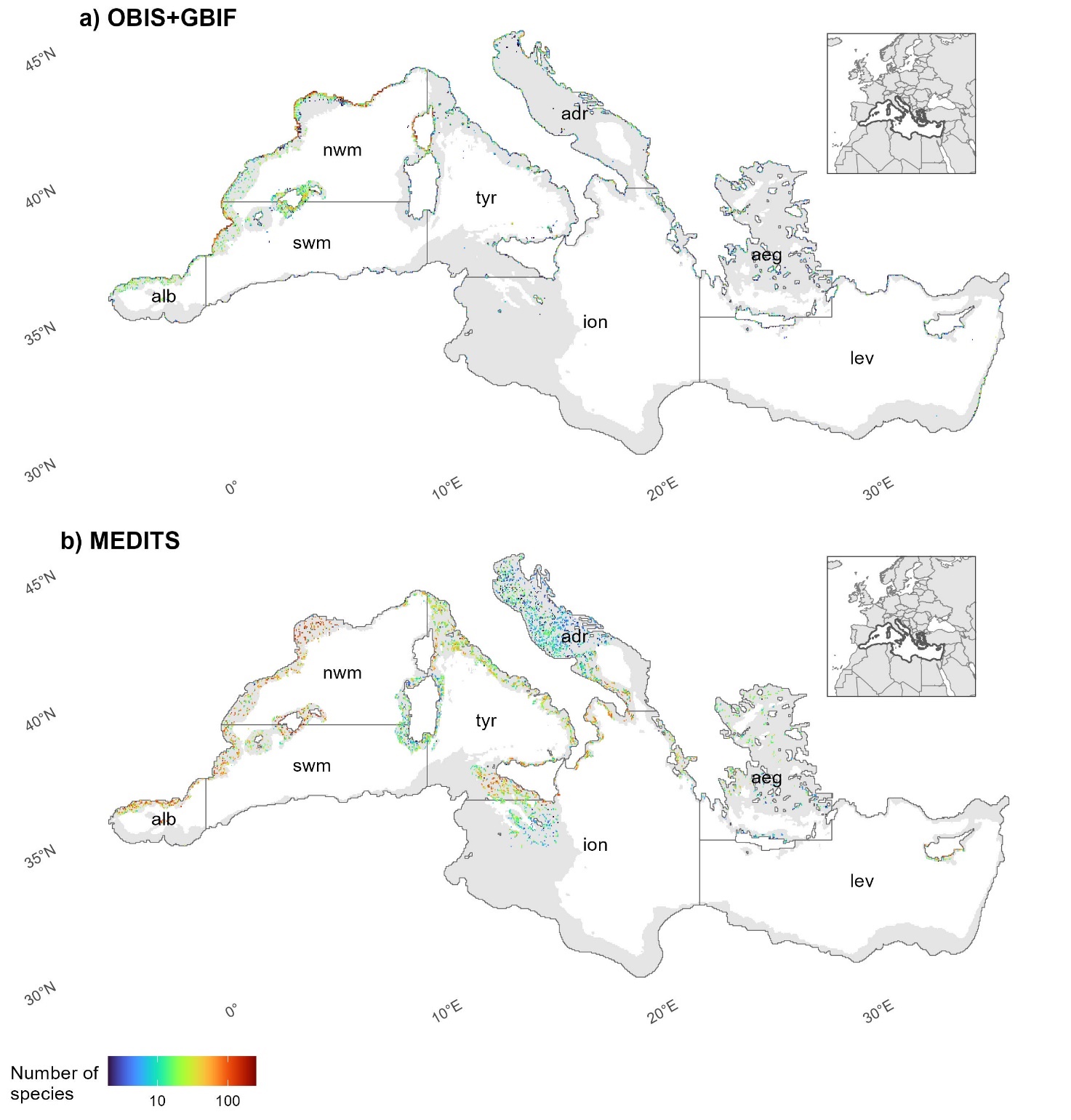


**Figure S1**  – Spatial distribution of points used to fit the models: OBIS and GBIC (top), and MEDITS (bottom). The gray-shaded area represents the fraction of the Mediterranean Sea with depth shallower than 800 m, where models were trained and projected. The insert on the bottom-left corner shows the geographic position of the studied area (borders are highlighted with a thick line). The insert on the top-right corner shows the correlation between the number of species and the depth at which species were recorded. The labels represent the Mediterranean basins used in the paper; alb: Alborean Sea; nwm: north-west Mediterranean; swm: south-west Mediterranean; tyr: Tyrrhenian sea; ion: Ionian sea; adr: Adriatic sea; aeg: Aegean sea; lev: Levantine sea.


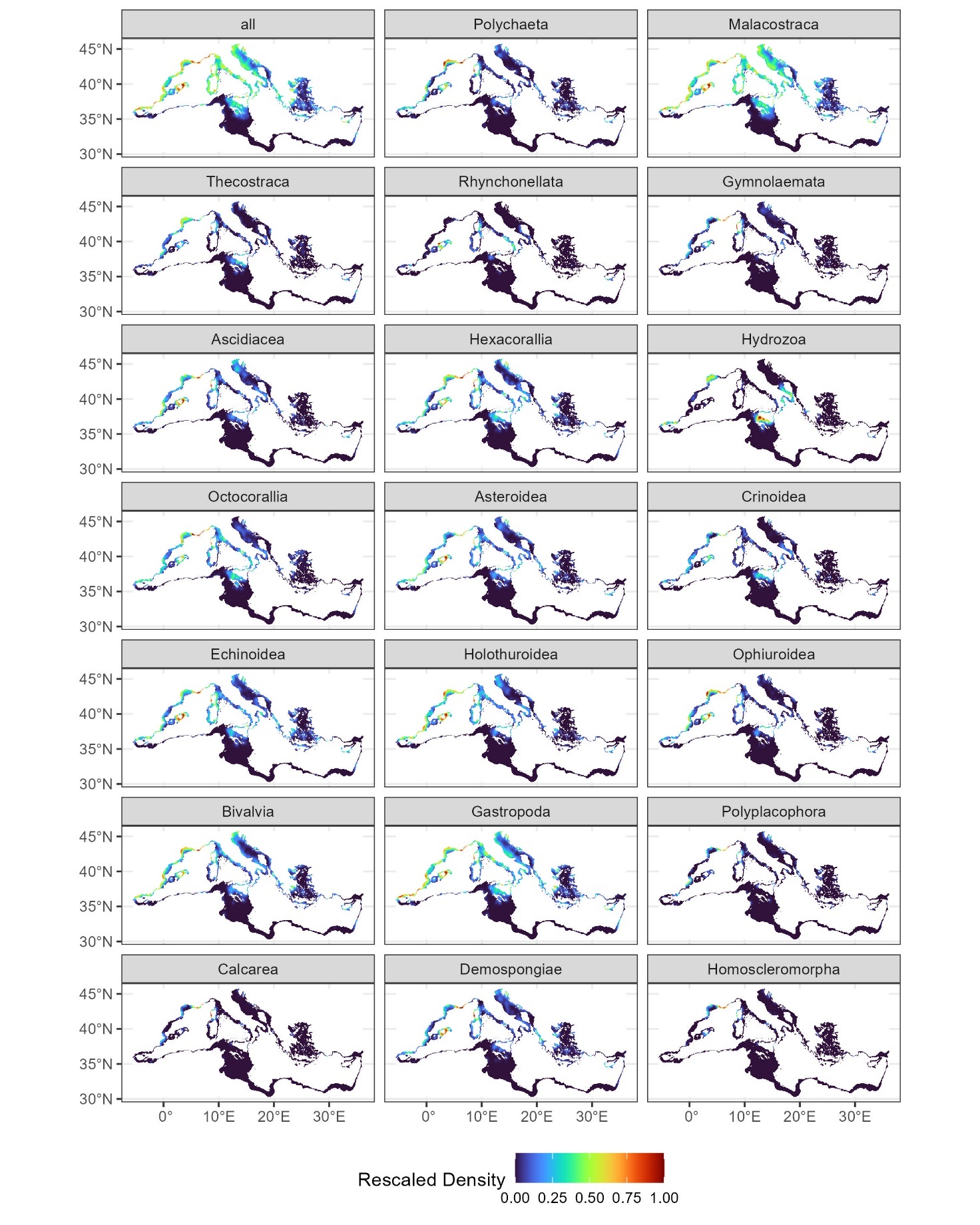


**Figure S2** – Class-specific density calculated based on kernel smoothing of the pooled occurrence points.


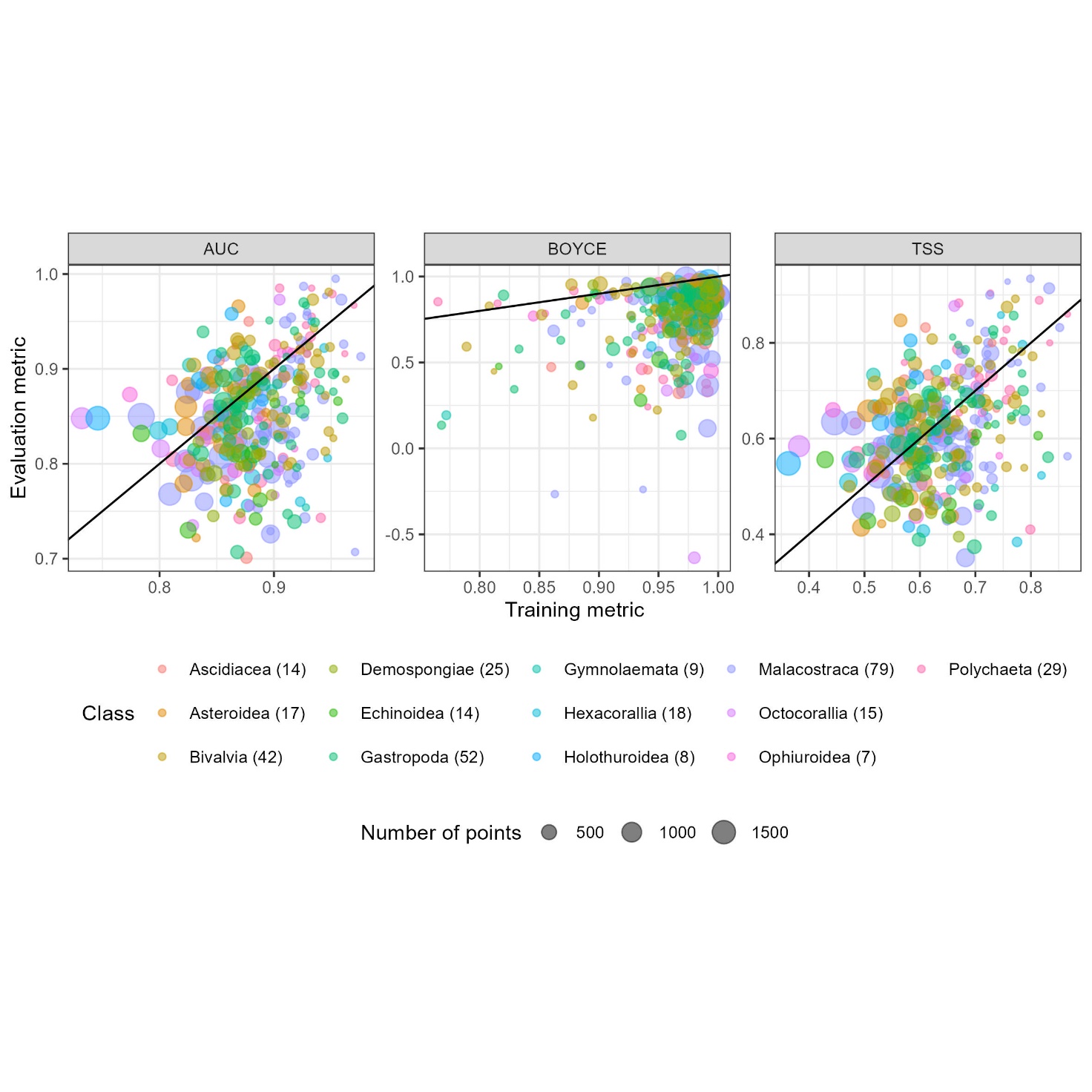


**Figure S3** – Scatterplot of the training vs evaluation metrics. The number of points used to fit the model is proportional to point size.


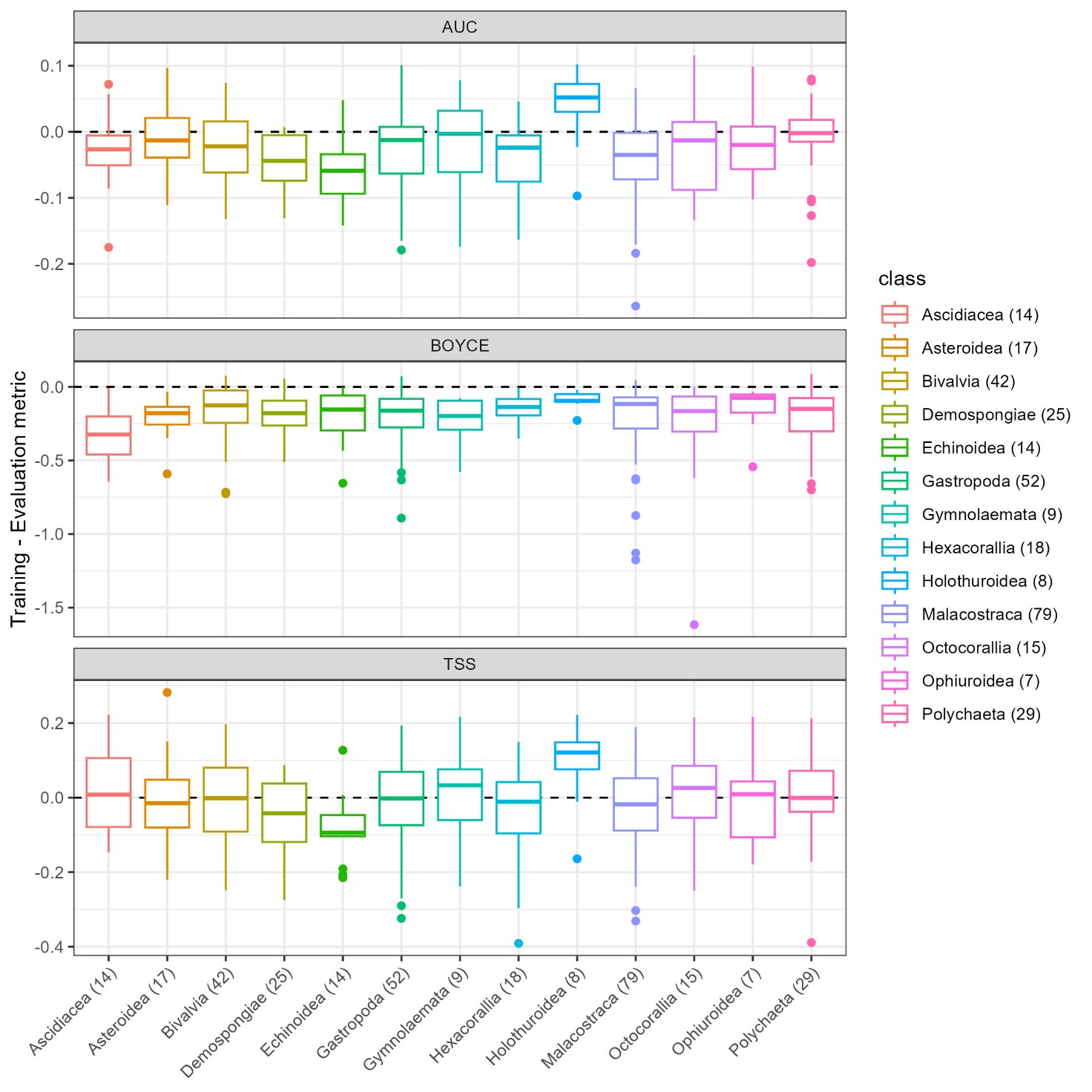


**Figure S4** – Difference between training and evaluation performance metrics, pooled by taxonomic class.


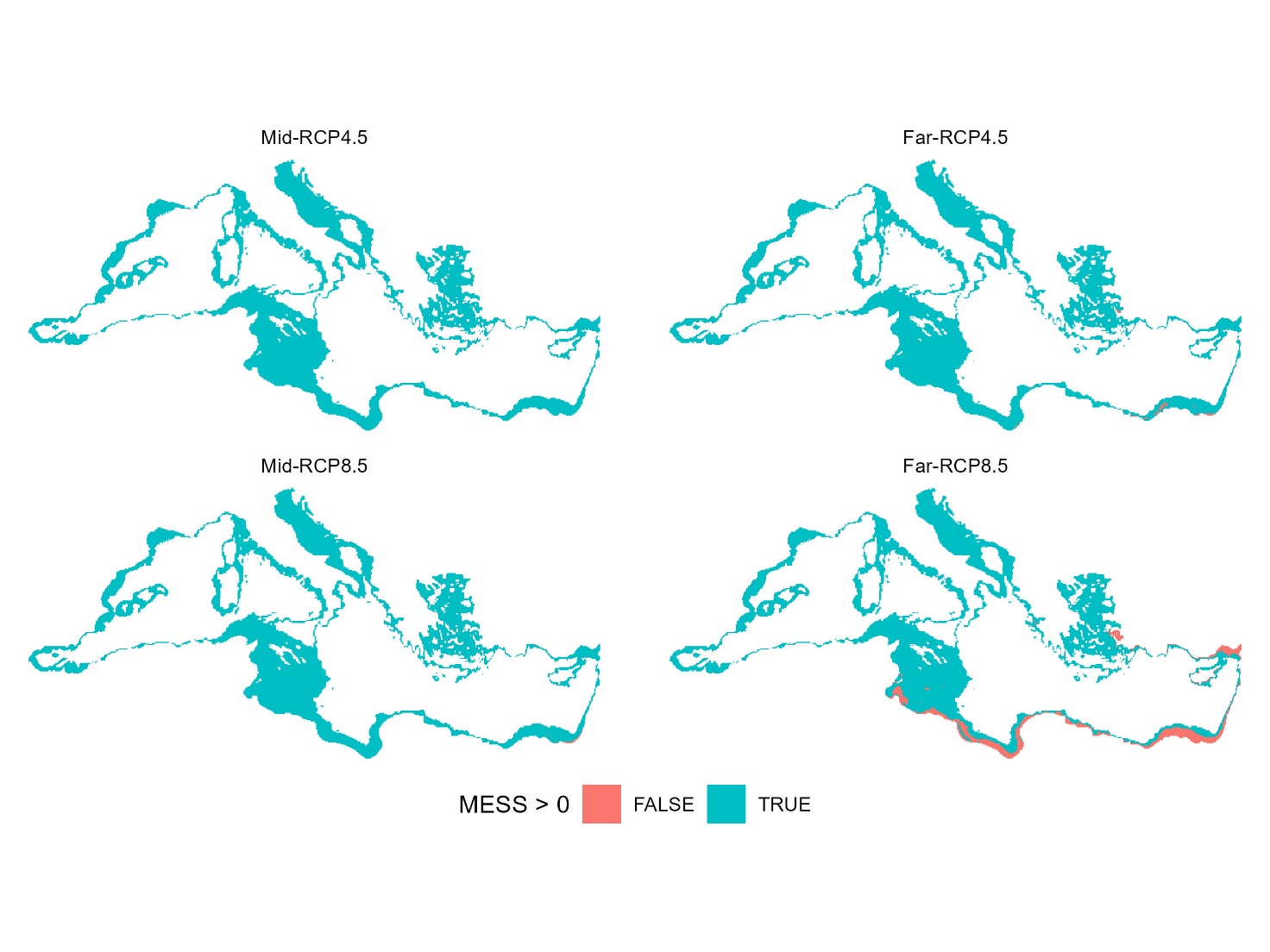


**Figure S5** – Geographic distribution of positive and negative values of the Multivariate Environmental Similarity Surface (MESS). Points where MESS < 0 (bright red in the map) are at high risk of extrapolation.


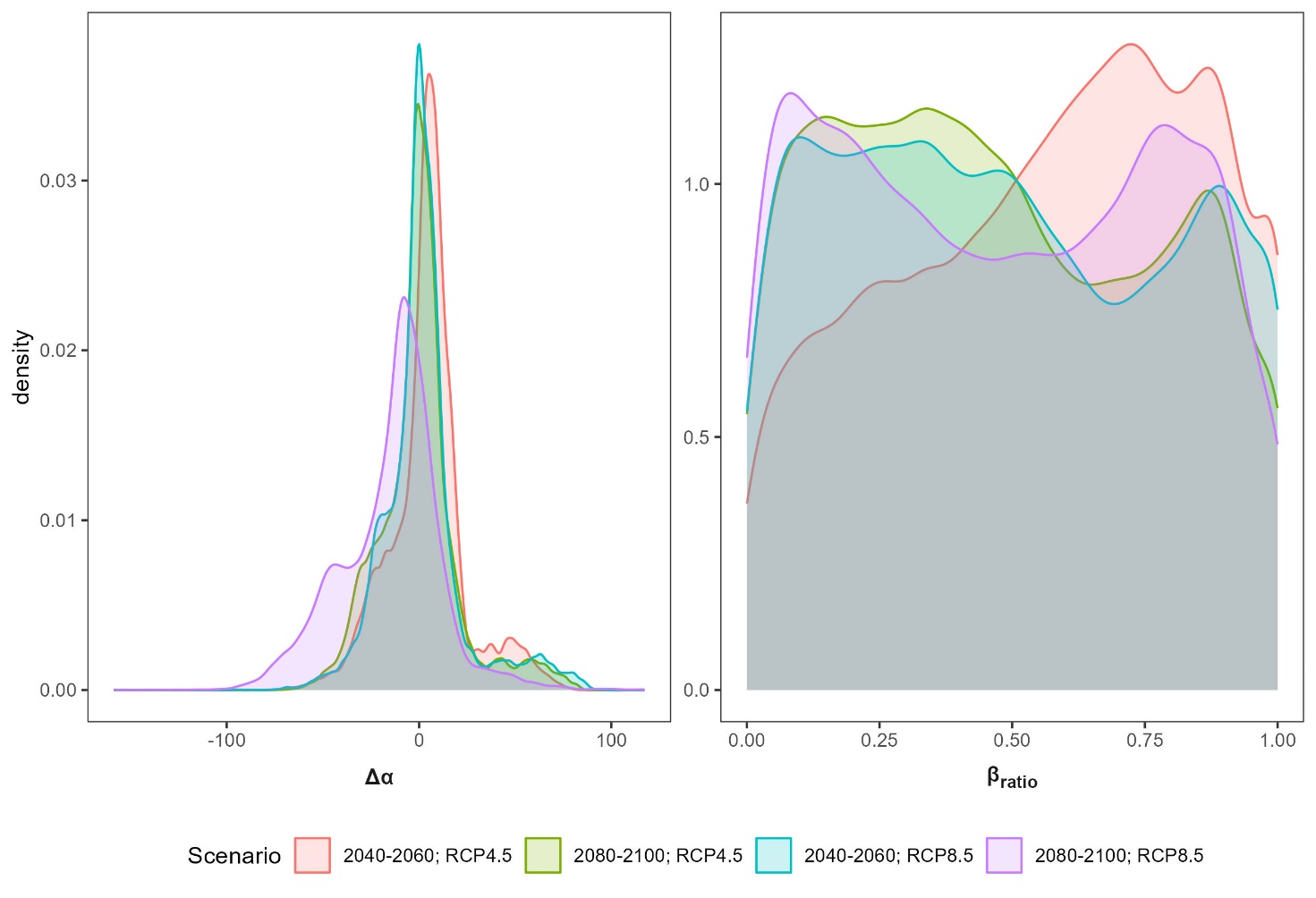


**Figure S6** – Density plots of Δα and β_ratio_ for the analyzed scenarios and time windows.


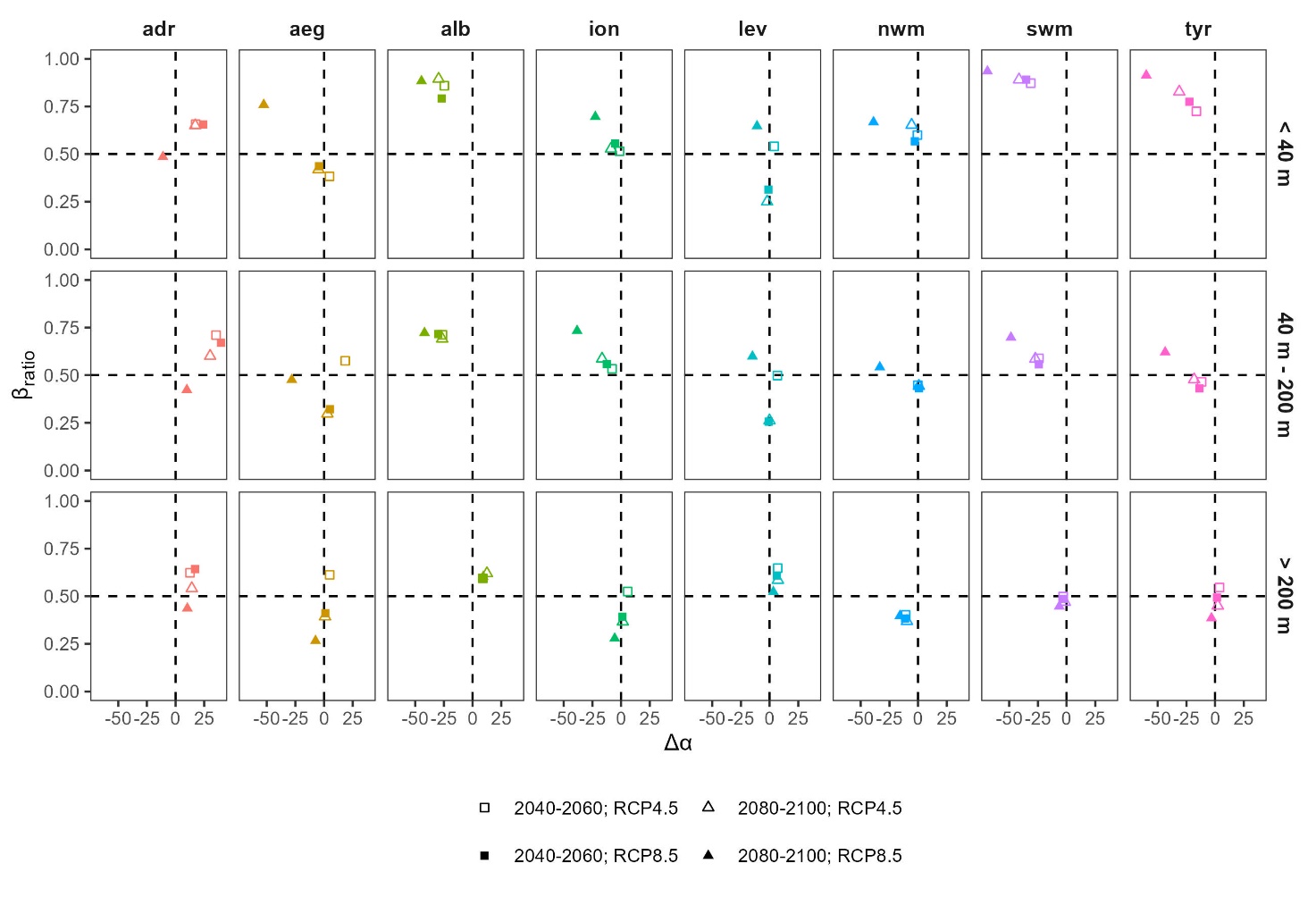


**Fig. S7** –Δα and β_ratio_ averaged over subbasins (see Fig. 1 for the geographic position) and depth zones.


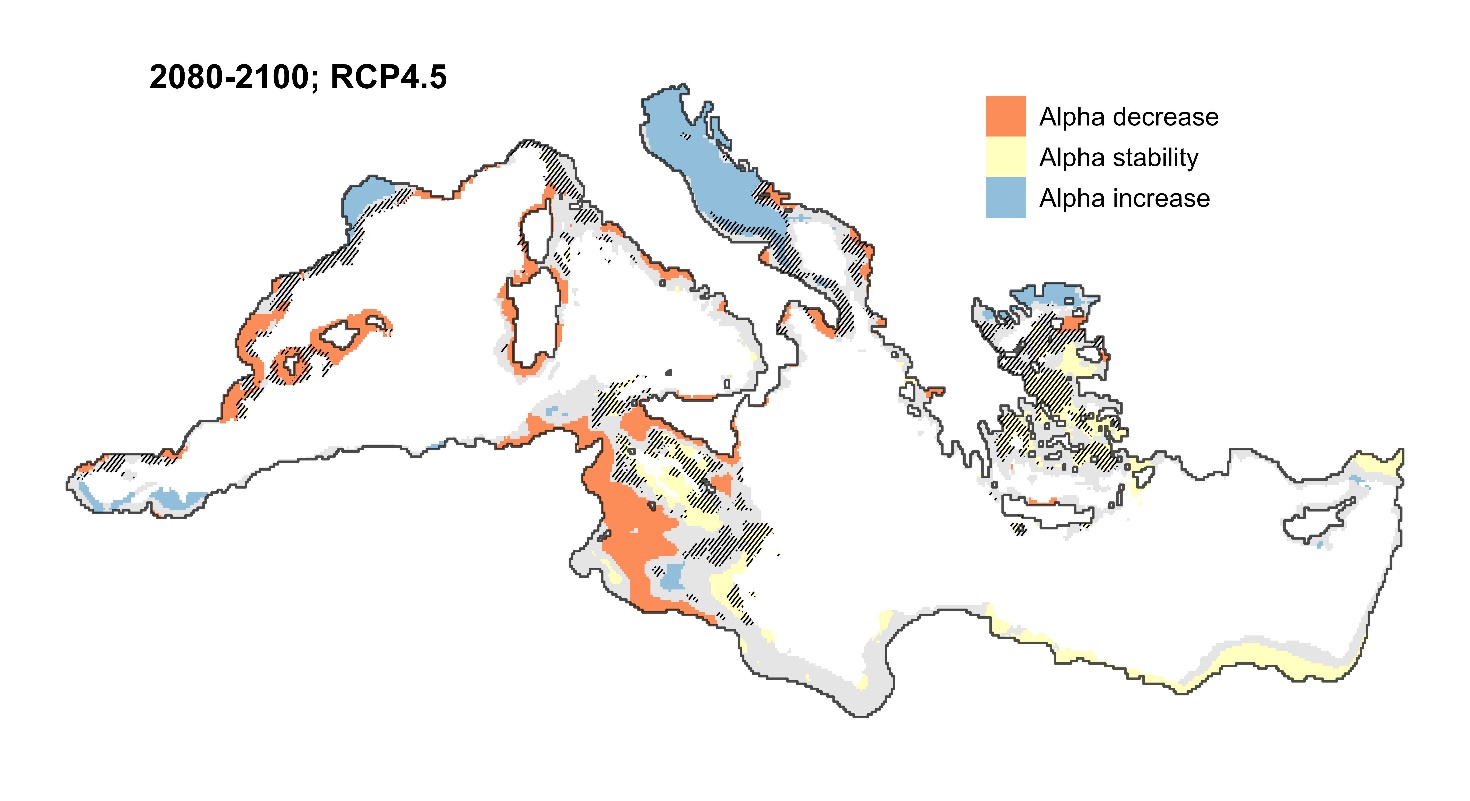


**Figure S8** – Spatial clusters of community change for the scenario RCP 4.5, time window 2080-2100. Clusters of community change were identified based on the Getis index, see Methods section 2.8. Blue, orange and yellow represents hot-, cold- and stability-spots of alpha diversity change, respecticely. Dashed lines represent hotspots of species turnover . Gray-shaded areas represents the fraction of the Mediterranean Sea with depth shallower than 800 m, where species distribution models were trained and projected.


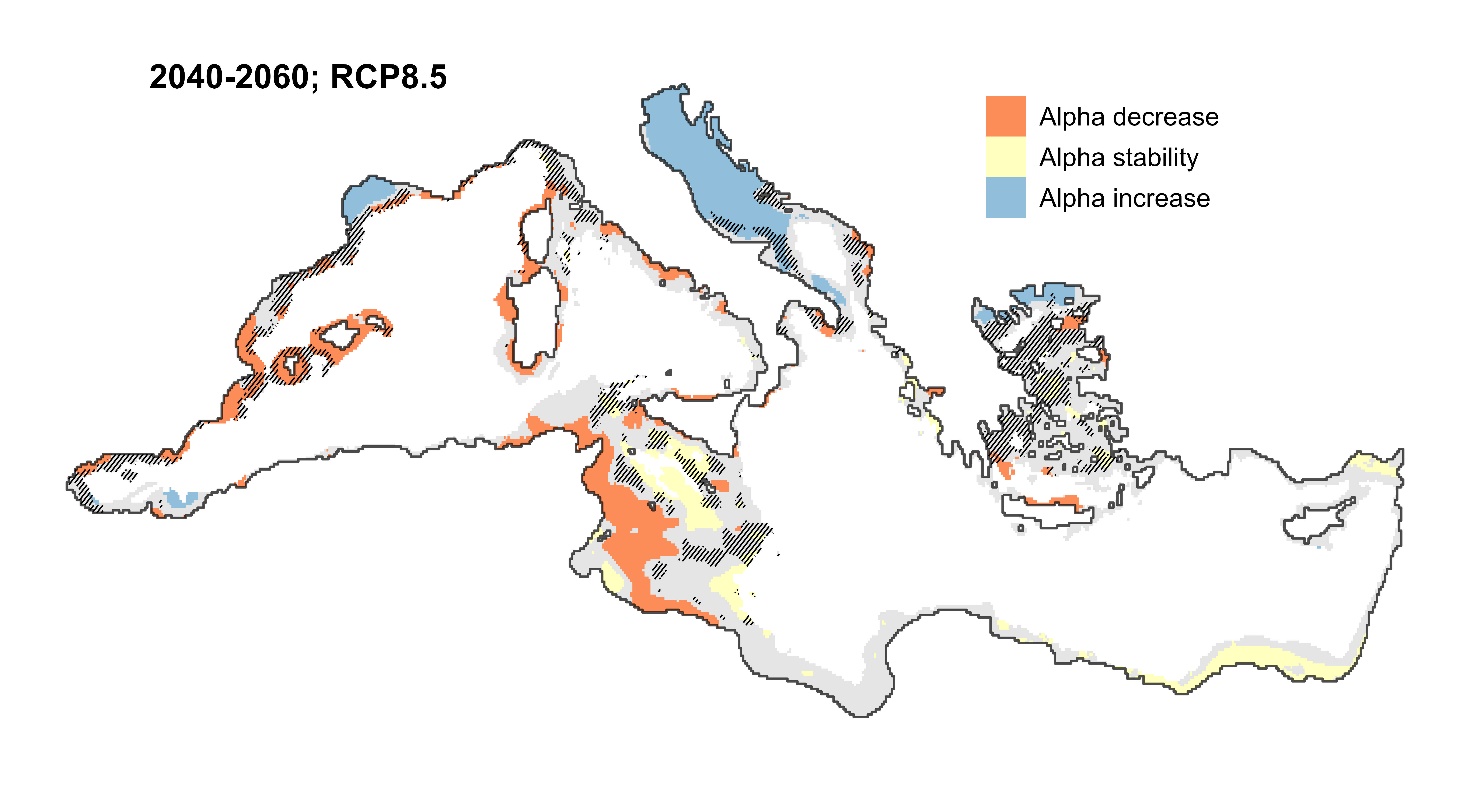


**Figure S9** – Spatial clusters of community change for the scenario RCP 8.5, time window 2040-2060. Clusters of community change were identified based on the Getis index, see Methods section 2.8. Blue, orange and yellow represents hot-, cold- and stability-spots of alpha diversity change, respecticely. Dashed lines represent hotspots of species turnover . Gray-shaded areas represents the fraction of the Mediterranean Sea with depth shallower than 800 m, where species distribution models were trained and projected.


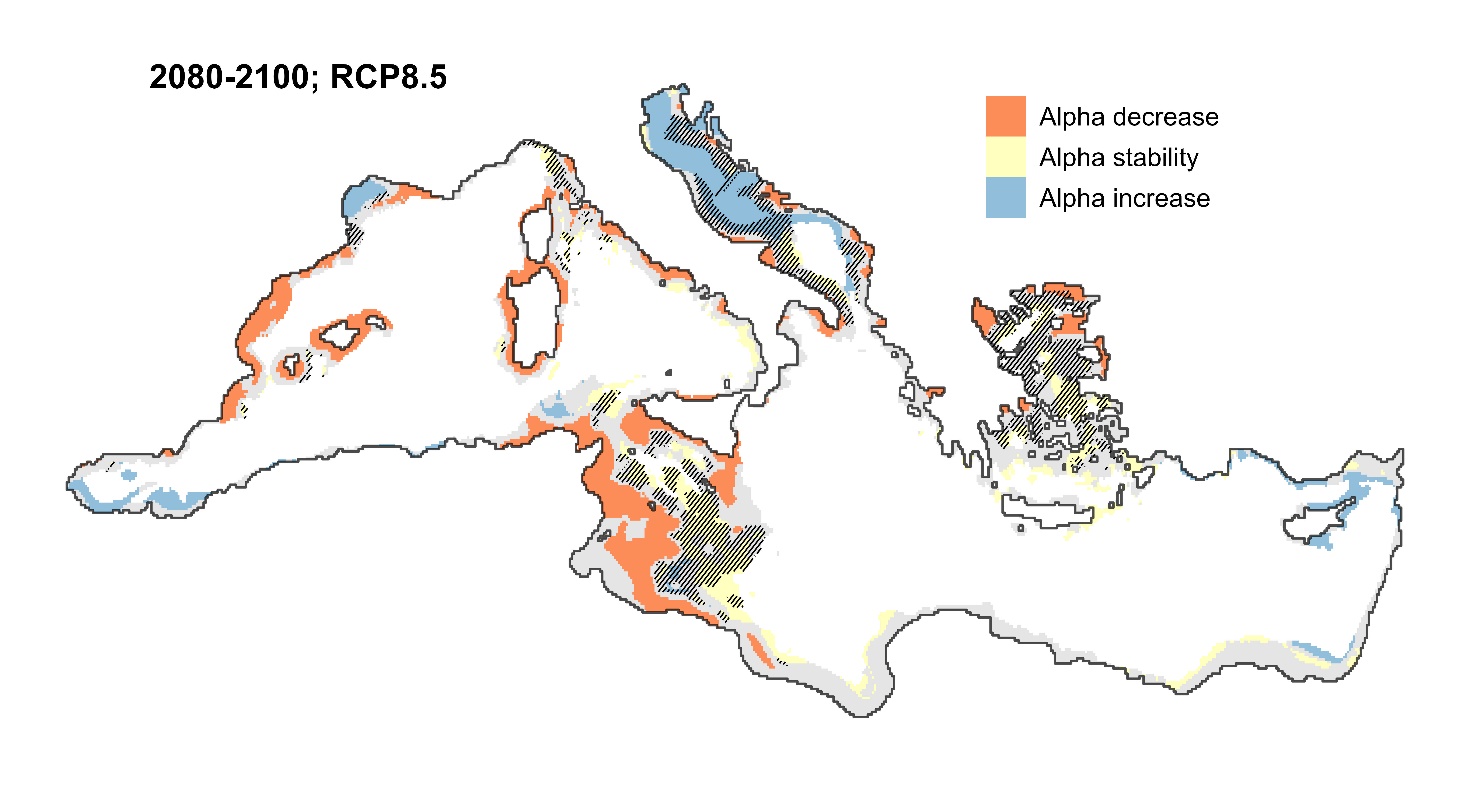


**Figure S10** – Spatial clusters of community change for the scenario RCP 8.5, time window 2080-2100. Clusters of community change were identified based on the Getis index, see Methods section 2.8. Blue, orange and yellow represents hot-, cold- and stability-spots of alpha diversity change, respecticely. Dashed lines represent hotspots of species turnover . Gray-shaded areas represents the fraction of the Mediterranean Sea with depth shallower than 800 m, where species distribution models were trained and projected.
